# T-regulatory cells require Sin3a for stable expression of Foxp3

**DOI:** 10.3389/fimmu.2024.1444937

**Published:** 2024-08-02

**Authors:** Lanette M. Christensen, Tatiana Akimova, Liqing Wang, Rongxiang Han, Arabinda Samanta, Eros Di Giorgio, Wayne W. Hancock

**Affiliations:** ^1^ Division of Transplant Immunology, Department of Pathology and Laboratory Medicine, The Children’s Hospital of Philadelphia, Philadelphia, PA, United States; ^2^ Department of Pathology and Laboratory Medicine, University of Pennsylvania, Philadelphia, PA, United States; ^3^ Department of Medicine, University of Udine, Udine, Italy

**Keywords:** T-regulatory cell, FOXP3+ Treg, SIN3A, transcription regulation, demethylation, autoimmunity

## Abstract

Histone deacetylases 1 and 2 play a major role in the transcriptional regulation of T-regulatory (Treg) cells via interactions with a myriad of coregulatory factors. Sin3a has been well established as a Hdac1/2 cofactor, while its role within Tregs has not been established. In this study, the effects of conditional deletion of Sin3a within Foxp3+ Tregs were evaluated. Developmental deletion of Sin3a from Foxp3+ Tregs resulted in the rapid onset of fatal autoimmunity. Treg numbers were greatly reduced, while residual Tregs had impaired suppressive function. Mice also showed effector T-cell activation, autoantibody production, and widespread tissue injury. Mechanistically, Sin3a deletion resulted in decreased transcription of *Foxp3* with a complete lack of CNS2 CpG demethylation. In addition, Foxp3 protein stability was impaired with an increased ex-Treg population. Thus, Sin3a plays a critical role in the maintenance of Treg identity and function and is essential for the expression and stability of Foxp3.

## Introduction

Fundamental to host immune homeostasis, T-regulatory (Treg) cells constrain immune responses and maintain self-tolerance (1–6). Forkhead box P3 (Foxp3) is the master transcription factor (TF) of Tregs and controls their phenotype and suppressive function (1–6). Loss of Foxp3 function results in severe, often lethal autoimmunity, as seen in Scurfy mice and clinically in patients with IPEX (immune dysregulation, polyendocrinopathy, enteropathy, X-linked) syndrome (5–7). However, while Foxp3 is required, it is not sufficient to regulate the transcriptional profile of Tregs. Other TFs, such as Foxo, Runx1, Ets-1, and Stat5, plus cofactors contribute to the expression of *Foxp3* and regulation of the Treg transcriptome (8), and TFs such as Blimp1, Irf4 (9, 10), and Mef2d (11) act synergistically with Foxp3 within effector Tregs (eTregs).

In addition to TFs and cofactors, nucleotide and chromatin epigenetic modifications contribute greatly to the expression of *Foxp3* and the regulation of the Treg transcriptome. Nucleotide modifications regulate gene transcription through DNA methylation; hypo- or hypermethylated CpG sites of promoter/enhancer regions result in gene expression or silencing, respectively (12). CpG methylation takes place through DNA methyltransferase (Dnmt) activity, while CpG DNA demethylation occurs primarily through the actions of 10–11 translocation (Tet) methylcytosine dioxygenases (12). The extent of CpG methylation is of major importance to Foxp3 expression and the development of the Treg lineage (13–15). Enhancers conserved noncoding sequences (CNS1 and CNS2) of *Foxp3* become completely demethylated upon Treg lineage commitment, supporting Foxp3 expression and stability, and Tet-mediated epigenetic regulation is essential to the maintenance of Foxp3 expression and Treg phenotype (16–19).

Chromatin modifications regulate gene transcription via the alteration of nucleosome-forming histones. Histone modifications include methylation, phosphorylation, ubiquitylation, and various forms of acylation, including acetylation and crotonylation. Typically, histone acetylation occurs on lysine residues of histones and relaxes the chromatin (euchromatin formation), thus allowing TF and polymerase access to the DNA. Histone acetyltransferase (HAT) and histone deacetylase (HDAC) enzymes are responsible for acetyl addition or removal, respectively. Various HATs, including Cbp, p300, Tip60, and Pcaf, contribute to transcriptional regulation within Tregs, including that of *Foxp3* (20–24). Likewise, many HDACs are active within Tregs, with most having a direct effect on Foxp3, though with varying functional outcomes (25). Pharmacologic inhibition of HDAC activity can promote or impair Treg function, depending upon the HDAC target(s) (26, 27). HDACs commonly function as members of large multiprotein complexes. HDAC1 and HDAC2 interact with and function within multiple coregulatory complexes, including CoREST, NuRD, and Sin3a. Mass-spec analysis has shown these coregulatory complexes are present within Tregs and are thereby expected to influence their production and/or function (25). However, the extent to which the Sin3a complex contributes to the regulation of Foxp3 and/or the Treg phenotype has been unknown.

Originally noted as a transcriptional corepressor (28, 29), Sin3a is now considered a transcriptional coregulator (27, 30, 31). The canonical Sin3 coregulatory complex is composed of dimerized switch-independent protein 3a or b (Sin3a/b) scaffolding proteins, along with suppressor of defective silencing 3 (SDS3), sin3a-associated protein p30 (SAP30), FAM60 (32), RBBP4/7, and with enzymatically active components HDAC1/2 (33). Sin3a regulates gene transcription by modification of histone acylation specifically via lysine deacetylation (34) and/or decrotonylation (35, 36). Sin3a is highly conserved throughout mammalian species (37) and is required for mammalian embryogenesis (38–40), progenitor cell differentiation (41), T-cell development (39), and transcriptional responses to hypoxia (42). The use of Sin3-specific peptide inhibitors, in addition to avermectin, impairs tumor growth in models of triple-negative breast cancer (43), demonstrating that the Sin3 complex may be a useful target for therapeutic development.

In this study, we evaluated the role of the scaffolding protein, Sin3a, in Foxp3+ Tregs. We found that Sin3a regulates the transcription of a large and diverse set of genes, including those specific to Tregs. Importantly, Sin3a was found to support the expression and stability of Foxp3, along with the Treg phenotype and suppressive function. Hence, Sin3a plays a vital role within Tregs and could potentially be targeted in certain contexts, such as decreasing Treg function to thereby boost host immune responses in cancer (44).

## Methods

### Mice and cell lines

We purchased Ai9(RCL-tdT) (stock No. 007909) and tamoxifen (TAM)-inducible Foxp3cre (stock No. 016961) mice from The Jackson Laboratory, in addition to previously described Foxp3^YFP-cre^ (45), CD4^cre^ (46), and Sin3a^flox/flox^ (47) mice. Sin3a^−/−^Foxp3^YFPcre^ mice and Foxp3^YFPcre^ controls were used 12–15 days after birth due to the early onset of lethal autoimmunity in mice with conditional deletion of Sin3a in their Tregs, whereas inducible knockout (KO) mice were used at 6–8 weeks. Mice were housed and handled in accordance with the IACUC (protocol No. IAC-22–000561) and The Children’s Hospital of Philadelphia Research Institute policy. The 293T cell line used in this study was obtained from the ATCC repository (CRL-3216).

### Histology

Sections of formalin-fixed, paraffin-embedded tissues were stained with hematoxylin and eosin (H&E) and analyzed in a blinded manner by a pathologist.

### Detection of autoantibodies

Cryosections (4–5 μM) of unfixed normal tissues were incubated for 1 h at room temperature (RT) with Sin3a^−/−^Foxp3^YFPcre^ sera, washed, incubated for 30 min at RT with FITC-labeled goat antimouse secondary Ab, washed, and mounted. Precharacterized sera from mice with known autoantibodies were used as positive controls, while pooled normal sera and secondary Abs alone were used as negative controls.

### Cell dissociation and cryopreservation

Tissues collected from mice were homogenized using a pestle over 0.4 µm filter fabric, dissociated cells collected in cold PBS, erythrocytes lysed using ddH_2_O, and single-cell suspensions used in same-day experiments or cryopreserved (48). Throughout, mammalian cells were centrifuged at 300×*g* for 10 min at 4°C, unless otherwise specified. Cell numbers and viability were determined using a Nexcelom Cellometer Auto 2000 cytometer and slides with AOPI viability dye (Nexcelom Bioscience, Lawrence, MA, USA). For cell enumeration and flow cytometry analysis, eight subcutaneous lymph nodes and four mesenteric lymph nodes (LN), the spleen, and the thymus were collected and processed from each mouse. For experiments that utilized bead-isolated or sorted Tregs, the spleen and around 15 LN (secondary lymphoid organs [SLOs]) from each mouse were dissociated, and when necessary, as for Sin3a^−/−^Foxp3^cre^ mice, were pooled for processing.

### Flow cytometry

Single-cell suspensions from subcutaneous and mesenteric LN, spleens, and thymi were prepared as described above. Single-cell suspensions of fresh or reconstituted cryopreserved cells were stained with fluorophore-conjugated mAbs listed in **Supplementary Table S1**. Cells were stained with fixable viability dye for 15 min at RT, stained for cell-surface antigens for 45 min at 4°C, and, as necessary, fixed and permeabilized (BD, Pharmingen Transcription Factor Buffer Set, BD, Franklin Lakes, NJ, USA) according to the manufacturer’s protocol prior to staining cytosolic and/or intranuclear targets for 1 h at 4°C. When staining for chemokine or cytokine production, cells were incubated with phorbol 12-myristate 13-acetate (PMA), ionomycin, and brefeldin for 3 h prior to fixation. For annexin V staining, cells were incubated in annexin-binding buffer (Invitrogen, Carlsbad, CA, USA) according to the manufacturer’s protocol and stained with annexin V Ab (**Supplementary Table S1**). For fluorescence-activated cell sorting (FACS) staining, cells were centrifuged at 1,300 rpm for 5 min and kept in the dark (or limited light) as described in the current FACS staining guidelines (49). Data from stained cells were acquired on a Cytoflex (Beckman Coulter, Brea, CA, USA) flow cytometer and analyzed using FlowJo v10 software.

### Fate mapping

Foxp3^YFPcre^ mice were crossed with Ai9(RCL-tdT) (JAX stock No. 007909), and progeny were crossed with Sin3a^flox/flox^. Dissociated cells from thymi and peripheral lymphoid organs were tested for tdT+YFP+ Tregs and tdT+YFP− exTregs using flow cytometry (**Supplementary Figure S17A**). Ai9 and Foxp3^YFPcre^Ai9 mice were used as controls for comparison with Sin3a^flox/flox^Foxp3^YFPcre^R26tdT KO mice.

### Treg suppression assay

Sin3a^−/−^CD4^cre^, CD4^cre^ Tregs, and CD4+CD25+ cells were isolated from SLOs using the CD4+CD25+ Treg Isolation Kit (Miltenyi Biotec, Gaithersburg, MD, USA). When using Sin3a^−/−^Foxp3^YFPcre^ or TAM_Sin3a^−/−^Foxp3^YFPcre^ with corresponding controls, Tregs were sorted on Foxp3^YFP+^CD25^PE+^ (BD FACSJazz sorter) from CD4+CD25+ bead-isolated cells. Foxp3 purity of isolated Tregs, determined by FACS, was confirmed to be ≥ 92% for bead-isolated cells and ≥ 97% for sorted cells, while not exceeding 1% difference between comparative groups. SLO responders, labeled with carboxyfluorescein succinimidyl ester (CFSE) (50), were seeded in 96-well round bottom plates (1 × 10^5^ cells/well) with serially diluted Tregs in standard T-cell media (RPMI-1640 with 10% FBS, l-glucosamine, and penicillin/streptomycin) containing sCD3mAb (1 μg/ml). Cells were coincubated at 37°C and 5% CO_2_ for 72 h, and proliferation was determined by flow analysis of CFSE division peaks. Prior to flow cytometry, cells were stained with fixable viability dye (Ghost 510) and then CD4 and CD8a mAbs (**Supplementary Table S1**). Area under curve (AUC) was calculated (48) using percent CFSE+ division of each Treg-to-responder cell ratio (1:1, 1:2, 1:4, 1:8, 1:16, and 0:1) per replicate. AUCs were expressed as ratios to CTRs, and ratios from individual experiments were averaged and tested for significance (one-sample *t*-test with a theoretical mean of 1).

### iTreg conversion assay

CD4+CD25− cells were isolated with a CD4+CD25+ Treg Isolation Kit (Miltenyi Biotec) from Sin3a^−/−^CD4^cre^, Sin3a^−/−^Foxp3^YFPcre^, or TAM_Sin3a^−/−^Foxp3^YFPcre^ with corresponding Cre controls. Percent Foxp3 of isolated CD4+CD25− cells, determined by FACS, was confirmed to not exceed 1% difference between comparative groups prior to treatment. CD4+CD25− cells were seeded in round-bottom 96-well plates (1 × 10^5^ cells/well) containing interleukin (IL)-2 (25 U/ml), tumor growth factor beta (TGF-β; 3 ng/ml), and CD3/CD28 beads at 37°C and 5% CO_2_ for 4 days (51). The percentage of Foxp3+ cells was determined by flow cytometry. Cells were stained using fixable viability dye (Ghost 510), followed by CD45 and CD4 mAbs, fixed, permeabilized, and stained for Foxp3. Cells from Foxp3^YFPcre^ mice were not fixed or permeabilized for Foxp3 staining, as they have YFP-tagged Foxp3. Results were recorded on a Cytoflex flow cytometer and analyzed with FlowJo 10.1 software.

### DNA methylation detection

Cells dissociated from SLOs of Sin3a^−/−^Foxp3^YFPcre^ and Foxp3^YFPcre^ controls were CD4+ and CD25+ enriched using magnetic bead isolation, then Foxp3^YFP^+CD25^PE^+ Tregs were collected by FACS sorting. DNA was isolated from Foxp3+CD25+ Tregs using the Qiagen DNA Kit as per the manufacturer’s protocol. Bisulfite conversion and desulfination of DNA (~ 300 ng/sample) was done using the Methyl Detection Kit by Active Motif. The CNS2 promoter region (aka TSDR) of *foxp3* was amplified using nested primer sets (outer set: “Foxp3_TSDR outer F” and “Foxp3_TSDR outer R” and inner set: “Foxp3_TSDR inner F” and “Foxp3_TSDR inner R”) detailed in **Supplementary Table S2** and described previously (52). The CNS2 PCR product was verified by and purified from agarose gel (1% agarose, 1× Tris-acetate-EDTA (TAE), 1 k, and 100 bp ladder, stained with Ethidium Bromide (EtBr)), using the QIAquick Gel Extraction Kit as per the manufacturer’s protocol. The converted CNS2 region was inserted into a TOPO2.1 TA vector using T4 DNA Ligase (Invitrogen) and transformed into TOP10 chemically competent *Escherichia coli* (Invitrogen) according to the manufacturer’s protocol. Transformed *E. coli* were selected from LB agar plates containing ampicillin (100 μg/ml) and galactose (40 μm/ml). Plasmids were isolated (Invitrogen PureLink Quick Plasmid Miniprep Kit, following the manufacturer’s protocol) from overnight cultures, and the presence of CNS2 inserts was confirmed via restriction enzyme digestion with EcoRI (NEB) followed by gel electrophoresis (1% agarose gel, 1× TAE, 1 k, and 100 bp ladder, stained with EtBr). The plasmids (meeting quality control measures of OD260/280 ≥ 1.8 and OD260/230 ≥ 2.0) were then sequenced (Sanger) using both forward (M13 Forward −21) and reverse (M13 Reverse −26) priming per sample (**Supplementary Table S2**) by the DNA Sequencing Facility at the University of Pennsylvania. Sequencing results were analyzed using methylKit packages in RStudio. Throughout these procedures, DNA concentration and quality were measured with a Nanodrop 2000 (Thermo Fisher Scientific, Waltham, MA, USA), and as necessary, Zymo Research DNA Clean and Concentrator Kit was utilized according to the manufacturer’s protocol.

### Coimmunoprecipitation

293T cells were cotransfected with Sin3a and either Foxp3, Hdac1, or Hdac2 plasmids using Lipofectamin 3000, according to manufacturer’s protocol. After 48 h, cells were washed with cold PBS and lysed on ice with lysis buffer containing 20 mM Tris-Cl at pH 7.5, 1% Triton-X100, 150 mM NaCl supplemented with protease and phosphatase inhibitor cocktails in addition to 1 mM DTT, 1 mM EDTA, 1 mM PMSF (just before lysates preparation). Cells were sonicated for 5 min in an ice bath (50% efficiency), rotated at 4°C for 30 min, and centrifuged at 12,000 rpm for 12 min. Proteins were estimated from clear supernatants using BCA reagents, using BSA as a standard. Equal amounts of protein lysates were precleaned with 2 µg of nIgG/tube (mouse or rabbit, depending on the IP antibody) for 1 h. Prewashed 30 µl protein A/G magnetic beads were added to each tube and rotated for 30 min at 4°C. Using a magnetic separator, precleaned lysates were collected, and equal amounts of lysates containing ~ 300 µg total protein immunoprecipitated with 5 µg each of nIgG, Sin3a, Hdac1, Hdac2, or other antibodies for 2 h with rotation at 4°C. Next, 30 µl prewashed magnetic beads were added to each tube and rotated for 1 h at 4°C. The IP beads were separated in the magnetic separator and washed four times with 500 µl of lysis buffer each time. Finally, IP beads were heated at 100°C in 1× sample lysis buffer for 10 min. Immunoprecipitated proteins were separated by 4%–15% SDS-PAGE. Western blots were developed with primary antibody for 2 h, washed four to five times, and then light chain-specific second antibody-conjugated horseradish peroxidase (HRP) was used for development with ECL reagents. In some experiments, antirabbit secondary antibody-HRP conjugates were also used.

### RT-qPCR and RNAseq

RNA was isolated from bead-isolated CD4+CD25+ cells of the CD4^cre^ background or from sorted CD25^PE^+Foxp3^YFP^+ Tregs of the Foxp3^YFPcre^ background using TRIZOL reagent (Ambion, Waltham, MA, United States) according to the manufacturer’s protocol. Sin3a^−/−^Foxp3^YFPcre^ and Foxp3^YFPcre^ control Foxp3^YFP^+CD25^PE^+ Tregs were sorted (FACS) following CD4+CD25+ enrichment by magnetic bead isolation (Miltenyi). For qPCR, mRNA was converted to cDNA using Reverse Transcriptase (Applied Biosystems, Carlsbad, CA, USA) with a BioRad C1000 thermocycler. TaqMan Gene Expression Master Mix (Applied Biosystems) was used with TaqMan primer sets listed in **Supplementary Table S2** as per manufacturer recommendations. qPCR target amplification was detected using a StepOnePlus Real-Time PCR System by Applied Biosystems. Genes commonly used as experimental controls for transcription, such as HPRT and GAPDH, were differentially expressed in Sin3a^−/−^ Tregs (**Supplementary Figure S16B**); therefore, 18S was utilized. Fold change was determined using the formula 2^−ΔΔCt^, where data were normalized to endogenous 18S and control target expression. Concentrations and quality of mRNA and cDNA were determined using a ThermoScientific Nanodrop 2000, and when necessary, RNA was cleaned and/or concentrated using the Zymo Research RNA Clean and Concentrator Kit according to the manufacturer’s protocol.

For RNAseq analysis, RNA was isolated from CD25+FoxP3+ Tregs that were sorted from CD4+CD25+ magnetic bead-enriched cells isolated from Sin3a^−/−^Foxp3^YFPcre^ or Foxp3^YFPcre^ controls. RNA samples met quality control measures of OD260/280 ≥ 2.0, OD260/230 ≥ 2.0, and integrity values ≥ 8.0 with a flat baseline (Agilent 5400). mRNA library preparation and sequencing (NovaSeq6000 PE150) were performed by Novogene Corporation Inc., Davis, CA, USA and data were deposited at the NCBI GEO site (accession No. GSE263830). Reads were mapped to the *Mus musculus* GRCm38/mm10 reference genome using Hisat2 v2.0.5. Read numbers mapped to each gene were quantified using featureCounts v1.5.0-p3, and FPKM was calculated. Differential expression analysis was performed using the DESeq2 R package 1.20.0, and the resulting *p*-values were adjusted using Benjamini and Hochberg’s approach for controlling the false-discovery rate. Differentially expressed genes (DEGs) were considered significant when the *p*-adjusted (*p*adj) value was ≤ 0.05 and the log_2_ fold change was ≥ 1 or ≤ −1. Data analysis, enrichment, and visualization were performed using Microsoft Excel, SnapGene, RStudio (53), GSEA_4.3.0 (54), and/or Cytoscape (55).

### Statistics

Statistical analysis and figure creation were done with Excel, RStudio, and/or GraphPad Prism software, unless otherwise stated. Experiments were performed with a minimum of three biological replicates in technical triplicates, unless otherwise specified. Parametric tests were applied if data were normally distributed, and nonparametric tests if not. Data are shown as the mean SEM. A two-tailed *p*-value of 0.05 or less was considered to be significant: *p ≤ 0.05; **p ≤ 0.01; ***p ≤ 0.001; and ****p ≤ 0.0001.

## Results

### Conditional deletion of Sin3a in Foxp3+ Treg results in rapid development of lethal autoimmunity

**Figure 1:**
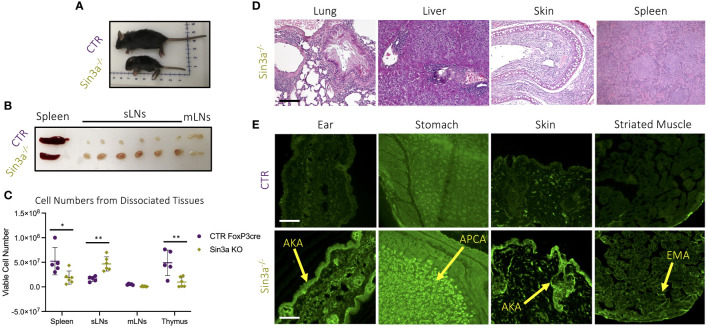
Gross pathology **(A)** of 12-day-old Sin3a^−/−^Foxp3^YFPcre^ (Sin3a^−/−^) and Foxp3^YFPcre^ (CTR) mice and secondary lymphoid organs **(B)**. Number of viable cells in single-cell suspensions of dissociated organs from either Sin3a^−/−^ or CTR mice with four to six biological replicates per group, consisting of two to three males and females **(C)**. Histopathology (×100) of formalin-fixed H&E-stained tissues from Sin3a^−/−^ mice **(D)** with CTR tissues included in **Supplementary Figure S1A**. Indirect immunofluorescent screening for autoantibodies in sera samples from Sin3a^−/−^ and CTR mice **(E)**. Autoantibodies: antikeratin Abs (AKA); antiparietal Abs (APCA); and antiendomysial Abs (EMA). Tissue section scale bars represent 100 μ. Symbols representing statistical significance are as follows: *p ≤ 0.05; **p ≤ 0.01; ***p ≤ 0.001; and ****p ≤ 0.0001.

To study the role of Sin3a in Treg cells, Sin3a^fl/fl^ mice were crossed with Foxp3^YFPcre^. Mice with homozygous deletion of *Sin3a* in Foxp3+ Tregs developed severe autoimmunity and died within 15–22 days of birth. These mice were smaller than normal littermates (**Figure 1A**), with ridged, dry skin and mucous membranes, and underdeveloped ears. Sin3a^−/−^Foxp3^YFPcre^ mice had markedly enlarged subcutaneous LNs (sLNs) and smaller spleens than littermate controls, while mesenteric LN (mLNs) were of normal size (**Figure 1B**); these differences were reflected in the number of cells recovered from dissociated organs (**Figure 1C**). Sin3a deletion within Tregs led to abundant tissue damage (**Figure 1D**; **Supplementary Figure S1A**). Histopathology of the lungs and livers showed loss of tissue structure, fibrosis, and edema, with immune cell infiltrates. Spleens displayed fibrosis and immune infiltrates, and skin showed tissue thickening, fibrosis, and immune infiltrates. Mice lacking Sin3a in Tregs produced abundant organ-specific autoantibodies including antikeratin Abs (AKA), antiparietal cells Abs (APCA), and antiendomysial Abs (EMA) (**Figure 1E**), and had heavy proteinuria (**Supplementary Figure S1B**). Together, these data showing extensive tissue injury and rapid death indicate that Sin3a perform a vital role in Treg cells, with consequences for prevention of autoimmunity.

### Mice with conditional Sin3a deletion in Foxp3+ cells have fewer Tregs and more effector T cells

**Figure 2:**
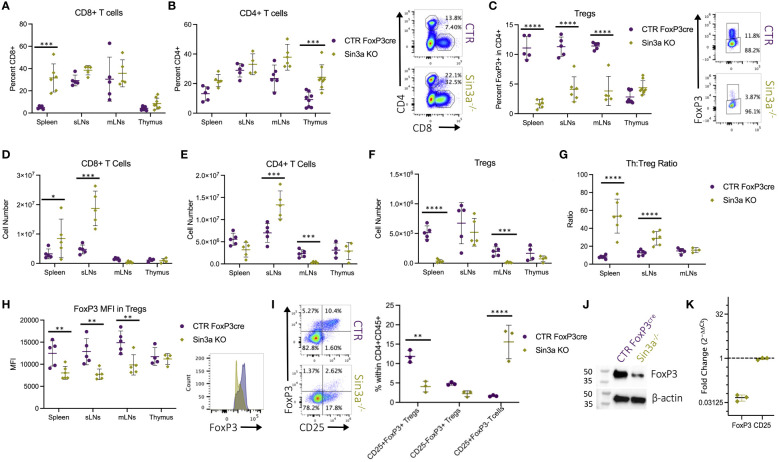
Percent of CD8+CD4− **(A)** and CD4+CD8−Foxp3− **(B)** within viable CD45+ cells and FoxP3+ Tregs **(C)** within viable CD45+CD4+ populations were determined by flow cytometry. Representative FCS plots from spleens included to the right, and FCS plots of sLN, mLN, and thymus data are included in **Supplementary Figure S3A**. Cell numbers of CD8+CD4− **(D)**, CD4+CD8−Foxp3− **(E)**, and Foxp3+ Tregs **(F)** calculated using FACS population percentages of cell numbers from corresponding dissociated organs (**Figure 1C**). The ratios of CD4+CD8−FoxP3− T cell (Th) to Foxp3+ Treg percentages in secondary lymphoid organs determined by flow cytometry **(G)**. Foxp3 MFI detected by flow cytometry with representative histograms of Sin3a^−/−^ (green) and Foxp3^YFPcre^ control (purple) from sLNs **(H)**. Representative FCS plots of CD25 and FoxP3 within viable CD45+CD4+ splenocytes with population percentages graphed on the right **(I)**. Western blot analysis of protein isolated from CD25+Foxp3+ FACS-sorted Tregs from pooled SLOs **(J)**. mRNA expression of Sin3a^−/−^ CD4+CD25+ bead-isolated cells from pooled SLOs was determined by RT-qPCR and normalized against 18S and control target expression **(K)**. FACS cell population analysis was performed using four to six biological replicates per group, consisting of two to three males and females. Symbols representing statistical significance are as follows: *p ≤ 0.05; **p ≤ 0.01; ***p ≤ 0.001; and ****p ≤ 0.0001.

Flow cytometric evaluation of T-cell populations in lymphoid organs shows that, compared to WT controls, Sin3a^−/−^ mice had more CD8+ T cells in their spleens (**Figure 2A**), more CD4+ T cells in their thymi (**Figure 2B**), and a marked reduction of Foxp3+ Tregs in SLOs (**Figure 2C**). Relations between control and Sin3a^−/−^ T-cell numbers differed from FACS percentages in some cases, e.g., subcutaneous LN from Sin3a^−/−^ mice were much larger (**Figure 1B**) and had more cells (**Figure 1C**) than controls. Within the massively enlarged sLN of Sin3a^−/−^ mice, the actual number of Tregs did not differ from controls (**Figure 2F**), while the numbers of CD8+ (**Figure 2D**) and CD4+ (**Figure 2E**) T cells increased greatly. FoxP3+ Tregs were nearly depleted in the spleens and mLN of Sin3a^−/−^ mice (**Figure 2F**), and CD4+ T cells were also reduced to near depletion in mLN (**Figure 2E**). In addition, within spleens and sLN, the ratios of CD4+ T cells to Tregs were much higher in Sin3a^−/−^ mice (**Figure 2G**), illustrating a cellular imbalance toward an effector phenotype. T cells from the SLOs of Sin3a^−/−^ mice were further examined using phenotypic markers for memory, naïve, and effector subpopulations (CD44 and CD62L). In the spleens and sLNs, but not mLNs, of Sin3a^−/−^ mice, the CD4+ and CD8+ T-cell populations were composed of more effector (CD62Llo) and fewer naïve (CD44+CD62L++) cells as compared to controls (**Supplementary Figures S2C–E**). This phenotypic skewing of CD4+ and CD8+ T cells from naïve toward effector in SLOs indicated a shift toward an activated effector cell environment.

### Conditional deletion of Sin3a in CD4+ T cells

The very low Treg numbers seen in Sin3a^−/−^Foxp3^cre^ mice markedly constrained *ex vivo* studies. We therefore deleted Sin3a in CD4+ cells, seeking to generate mice in which we could isolate Sin3a-deficient Tregs in sufficient numbers for further studies. The reasoning behind this was that Sin3a deletion from both suppressive and effector CD4+ T lineages would rescue the severe early-onset autoimmunity and fatality of Sin3a^−/−^FoxP3^cre^ deletion. Indeed, Sin3a^−/−^CD4^cre^ mice did not develop fatal autoimmunity. Rather, both CD4+ and CD8+ T-cell populations were drastically decreased, by percentage and cell numbers, in the SLOs of Sin3a^−/−^CD4^cre^ mice (**Supplementary Figures S4A, G**). Within SLOs, proportions of Foxp3+ cells were markedly increased compared to CD4^cre^ controls (**Supplementary Figure S4A**). While incongruent with the percentages of FoxP3+ cells within mice with conditional Sin3a deletion from Foxp3+ Tregs, these results are consistent with a recently published analysis of Sin3a^−/−^CD4^cre^ mice (56). However, when considering cell numbers within these organs, Foxp3+ Tregs were actually reduced in the spleens and sLNs (**Supplementary Figure S4G**). Tregs and CD4+ cells had reduced naïve and elevated effector subpopulations in the spleens, subcutaneous and mesenteric LNs (**Supplementary Figure S4D**). Naïve CD8+ T cells were also reduced in the SLOs (**Supplementary Figure S4D**). Memory CD4+ and CD8+ T cells were drastically increased in the SLOs, while the Treg memory population increased only in the sLNs (**Supplementary Figure S4D**). Hence, as in the Foxp3^cre^ background, Sin3a^−/−^CD4^cre^ mice displayed a cellular imbalance toward an activated immune environment.

### Inducible Sin3a deletion in existing Foxp3+ Treg cells

In further efforts to obtain larger numbers of *ex vivo* Foxp3+ Tregs lacking Sin3a, we utilized a tamoxifen-inducible Sin3a^−/−^Foxp3^cre^ (Sin3a^−/−^TAM) model. This allowed mice to develop normally prior to Sin3a deletion from Foxp3+ Tregs. Following tamoxifen treatment (**Supplementary Figure S5**), mice did not succumb to fatal autoimmunity, but like Sin3a^−/−^Foxp3^cre^ mice, they had massive sLNs (**Supplementary Figure S6A**) with pronounced follicles and germinal centers and developed PMN infiltrates and airway cuffing in the lungs (**Supplementary Figure S6B**). T-cell populations were assessed by FACS and had no significant differences in percentages of CD8+, CD4+, or Foxp3+ T cells (**Supplementary Figure S7A**). However, there was a significant downward shift in Foxp3 fluorescent intensity in Sin3a^−/−^TAM Tregs (**Supplementary Figure S7A**), like those from CD4^cre^ (**Supplementary Figure S4**) and Foxp3^cre^ (**Figure 2H**) backgrounds. T-cell subpopulations were also similar to those of the other two genetic backgrounds studied, whereby CD4+ cells and Foxp3+ Tregs had increased effector and decreased naïve types, especially in sLNs (**Supplementary Figure S7E**).

While there were differences in the percentages of Foxp3+ Tregs among Sin3a^−/−^ mice of Foxp3^cre^, TAM-induced Foxp3^cre^, and CD4^cre^ backgrounds, in each case Foxp3 was reduced compared to controls. The CD25/Foxp3 FACS profiles illustrate the depletion of Foxp3 and the shift from CD25+Foxp3+ to CD25+Foxp3− in Sin3a^−/−^Foxp3^cre^ mice (**Figure 2H**). The great loss of Foxp3 protein within Sin3a^−/−^ Tregs as determined by fluorescent intensity was substantiated by Western blot (**Figure 2J**). In addition, a massive reduction in transcriptional expression of *Foxp3* was detected by RT-qPCR with both CD4+CD25+ bead-isolated Tregs of the CD4^cre^ background (**Figure 2K**) and CD25+FoxP3+ sorted Tregs of the Foxp3^cre^ background (**Supplementary Figure S14B**). Hence, deletion of Sin3a from Tregs resulted in the reduction of Foxp3 protein and altered immune cell populations, leading to varying degrees of immune homeostasis disruption.

### Sin3a is important for Foxp3+ follicular Treg development

**Figure 3:**
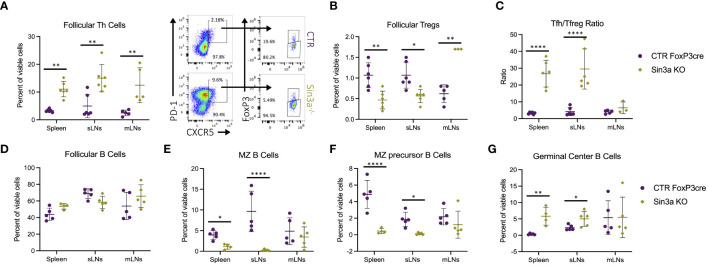
Follicular T-helper cell (CD45+CD4+PD1+CXCR5+FoxP3−) **(A)** and follicular T-regulatory cell (CD45+CD4+PD1+CXCR5+FoxP3+) **(B)** populations were determined using flow cytometry (representative FCS plots from the sLNs included in between) and then used to calculate the ratio of Tfh to Tfreg cells **(C)**. The percent of follicular B cells (CD19+B220+CD93−CD21loCD23hi) **(D)**, marginal zone (MZ) B cells (CD19+B220+CD93−CD21hiCD23lo) **(E)**, precursors to MZ B cells CD19+B220+CD93−CD21hiCD23hi) **(F)**, and germinal center B cells (CD19+B220+GL7+FAS+) **(G)** were determined by flow cytometry; representative FCS plots included in **Supplementary Figure S4**. Further details concerning gating strategies and representative FCS plots for follicular T- and B-cell subsets are included in **Supplementary Figures S8A, B**, **S9A–F**, respectively. FACS cell population analysis was performed using four to six biological replicates per group, consisting of two to three males and females. Symbols representing statistical significance are as follows: *p ≤ 0.05; **p ≤ 0.01; ***p ≤ 0.001; and ****p ≤ 0.0001.

Given enlarged lymphoid follicles and germinal centers in sLNs Sin3a^−/−^ mice (**Supplementary Figure S6B**), follicular immune cell populations were evaluated by flow cytometry. There were more follicular T helper (Tfh) cells in the SLOs of Sin3a^−/−^ mice (**Figure 3A**), whereas follicular T-regulatory (Tfreg) cells were decreased in spleens and sLNs (**Figure 3B**). The ratios of Tfh to Tfreg cells were much higher in the spleens and sLNs of Sin3a^−/−^ mice (**Figure 3C**). Follicular B cells were unchanged (**Figure 3D**), while marginal zone (MZ) B cells (**Figure 3E**) and MZ precursor B cells (**Figure 3F**) were reduced in Sin3a^−/−^ spleens and sLNs. In contrast, germinal center (GC) B cells were increased in Sin3a^−/−^ spleens and sLNs (**Figure 3G**). While not exclusive to lymphoid follicles, B-regulatory cells were reduced in the sLNs of Sin3a^−/−^ mice (**Supplementary Figure S10**). A few other differences were observed in B-cell subtypes (**Supplementary Figure S9**). These results show that Sin3a has a profound effect on the cellular composition of the germinal follicles of SLOs.

### Sin3a is key to the transcriptional regulation of Foxp3+ Treg cells

**Figure 4:**
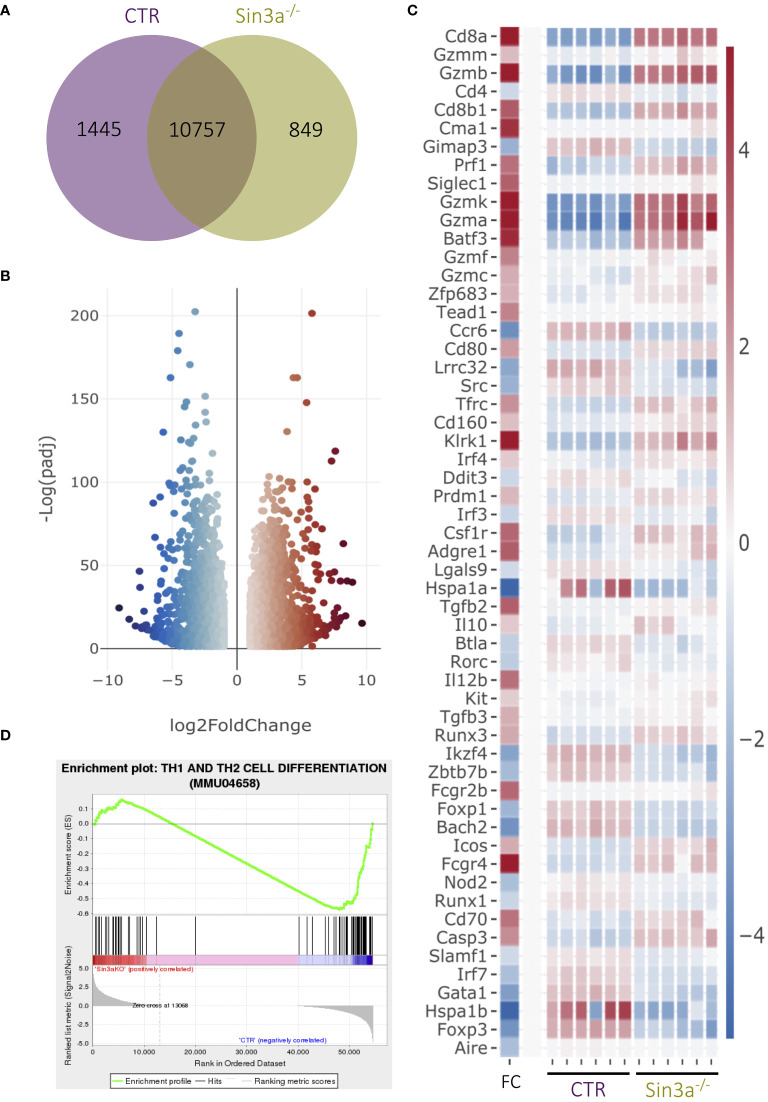
RNAseq analysis of sorted CD25+Foxp3+ Tregs from Sin3a^−/−^Foxp3^YFPcre^ or Foxp3^YFPcre^ control mice. Gene coexpression between groups is depicted in a Venn diagram **(A)**. DEGs of Sin3a^−/−^ vs. CTR Tregs with |log_2_ fold change|≥ 1 and *p*adj ≤ 0.05 in a volcano plot **(B)**, and selected DEGs depicted by heatmap with log_2_ fold change (FC) in the left column with the color scale to the right **(C)**. GSEA output using DEGs of Sin3a^−/−^ versus CTR Tregs (NES: − 1.338; *p*-value: 0.006; FDR: 0.156) **(D)**. RNAseq 3D PC plot (**Supplementary Figure S15A**), fpmk distribution violin plot (**Supplementary Figure S15B**), and total DEG heatmap (**Supplementary Figure S16A**) are included in the **Supplementary Materials**. RNAseq was performed using six pooled biological replicates per group, three males and three females.

We used RNAseq to better understand the extent to which Sin3a contributes to transcriptional regulation in Foxp3+ Tregs (**Figure 4**; **Supplementary Figures S14–S16**). There were hundreds of uniquely expressed genes in Tregs with and without Sin3a (**Figure 4A**). Among the genes differentially expressed between Sin3a^−/−^ and control Tregs, thousands of genes had enhanced or reduced expression (**Figures 4B, C**; **Supplementary Figure S16**), showing that Sin3a contributes to both positive and negative transcriptional regulation within Tregs, i.e., Sin3a acts as a coregulator in Treg cells and is not simply a corepressor.

Transcriptional expression of *Foxp3* was reduced in Sin3a^−/−^ Tregs (**Figure 4C**; **Supplementary Figure S16B**), consistent with reduced Foxp3 protein in Tregs as detected by flow cytometry (**Figures 2C, H, I**; **Supplementary Figures S4H**, **S7A**) and Western blot (**Figure 2J**). RNAseq also showed Sin3a^−/−^ Tregs had reduced expression of multiple TFs that contribute to the induction and stability of Foxp3 expression, including Foxo1, Fos, Jun, Ets-1, Rel, Runx1, Helios (Ikzf2), Eos (Ikzf4), Bcl-11b, and Bach2 (**Figure 4C**; **Supplementary Figure S16B**), as well as CD4, Tnfrsf18 (GITR), and Cxcr5 (**Figure 4C**; **Supplementary Figure S16B**). In contrast, factors produced by effector Tregs such as perforin, granzyme B, and IL-10 had greatly increased transcript levels (**Figure 4C**).

While transcriptional expression of Foxp3 and other Treg-specific factors was reduced in the absence of Sin3a, markers of other lineages and immune cell subtypes were enhanced, including T-bet, Blimp1, IRF4, CD63, CD8a, and CD8b1 (**Figure 4C**; **Supplementary Figure S16**). CD8a was the most significantly altered DEG (ranked by *p*adj value), and this was confirmed by increased CD8a protein in Foxp3+ Tregs via FACS (**Supplementary Figure S2F**). Other CD8+ T-cell-associated factors such as CD160, EOMES, and Batf3 were also increased in transcriptional expression (**Figure 4C**; **Supplementary Figure S16B**). Gene Set Enrichment Analysis (GSEA) of DEGs revealed that CD4 and CD8 T cell differentiation (**Figure 4D**) and Th17 cell differentiation (**Supplementary Figure S15M**) were impacted by the absence of Sin3a. Myeloid lineage and inflammatory mediators were also increased in Tregs lacking Sin3a, including GM-CSF (Csf2), Batf2, NFAT5, CD14, CD33, and CD36. Conversely, the majority of DEGs of B-cell-associated factors such as CD5, CD37, CD38, and CD79a/b (B-cell receptor [BCR]) were reduced (**Supplementary Figure S16B**). Together, these results display a markedly dysregulated transcriptional profile of Foxp3+ Treg cells, specifically favoring proinflammatory effector mechanisms.

Enrichment analysis of DEGs identified immune processes and hematopoiesis to be significantly altered by the Sin3a deletion (**Supplementary Figure S15D**). Likewise, GSEA revealed many signaling pathways involved in immune processes, such as NOD-like, NOTCH, MAPK, tumor necrosis factor (TNF), RIG I-like, and Toll-like signaling (**Supplementary Figures 15E–K**), were impacted. DEGs that were reduced or enhanced in Sin3a^−/−^ Tregs were cross-referenced with ATAC-seq data from an open-access repository, and the top four results from each query are included in **Table 1**. DEGs with reduced expression in Sin3a^−/−^ Tregs best-overlapped chromatin availability of Tregs. DEGs with enhanced expression in Sin3a^−/−^ Tregs best-overlapped chromatin availability of dendritic cells (DC), followed by monocytes. These results show that Sin3a supports transcriptional activation of Treg-specific factors and repression of alternative hematopoietic cell types. Hence, the Sin3a scaffolding protein plays a pivotal role in the control of the Treg transcriptional profile.

**Table 1:** Cooccurrence enrichment analysis of Sin3a^−/−^ Treg DEGs and chromatin availability in immune cells.

Data input (RNAseq)	ChIP-Atlas data type	Cell type	ChIP-Atlas experiment ID	Overlaps/DEGall	Overlaps/control	log *p*-value	Fold enrichment	ChIP-Atlas query ID
Sin3a^−/−^ Treg DEGs down	ATAC-seq	Tregs	DRX266827	1,954/2,321	10,643/17,403	− 115.8	1.38	wabi_chipatlas_2023-0712-1140–30-435–364336
		Tregs	DRX266831	1,958/2,321	10,702/17,403	− 114.8	1.37	
		Thymic cells	SRX12710507	2,137/2,321	12,575/17,403	− 114.2	1.27	
		CD4+ T cells	SRX18827479	1,985/2,321	11,020/17,403	− 112.0	1.35	
Sin3a^−/−^ Treg DEGs up	ATAC-seq	DCs	SRX2523844	2,429/2,711	11,295/17,013	− 153.9	1.35	wabi_chipatlas_2023-0728-0313–52-983–274731
		Monocytes	SRX7894782	2,436/2,711	11,410/17,013	− 150.8	1.34	
		Pre Gran/Mono	SRX9621071	2,420/2,711	11,266/17,013	− 150.8	1.35	
		CD8+ T cells	SRX14333165	2,295/2,711	10,208/17,013	−150.6	1.35	

### Sin3a is essential for Treg cell suppressive function

**Figure 5:**
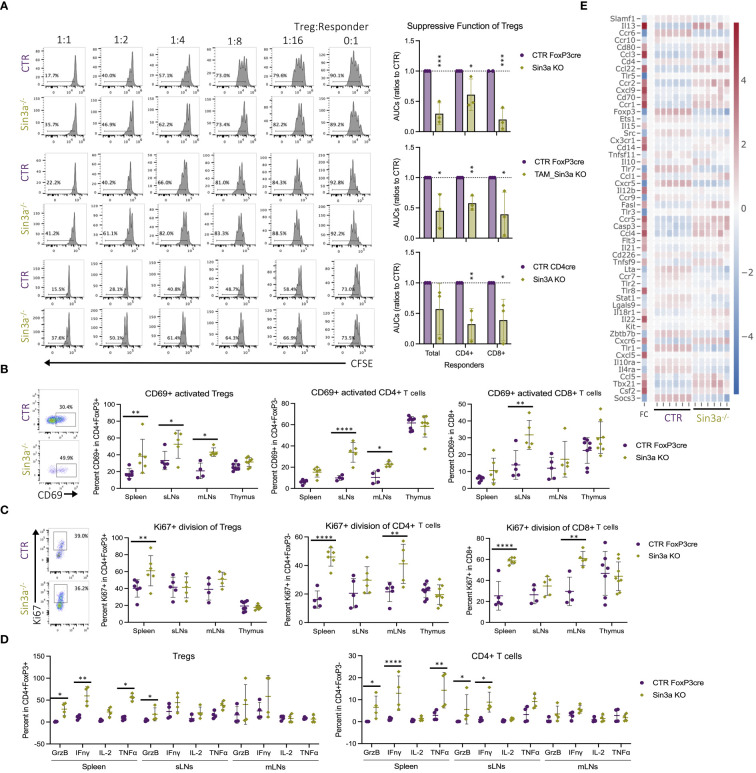
Foxp3+ Treg dysfunction in the absence of Sin3a. Treg suppression assays performed with Sin3a^−/−^ Foxp3^YFPcre^ (**A**, top), TAM-induced Sin3a^−/−^Foxp3^YFPcre^ CD25+FoxP3+ FACS-sorted Tregs (**A**, middle), or Sin3a^−/−^CD4^cre^ CD4+CD25+ bead-isolated Tregs (**A**, bottom) with control splenic responders. Suppression assay representative CFSE histograms of CD4+ responder division in ratios with serially diluted Tregs are on the left and the area under the curve (AUC) is calculated from the percent CFSE division on the right **(A)**. Percent CD69+ activation **(B)** and Ki-67+ division **(C)** of CD4+Foxp3+ Tregs (center-left), CD4+CD8−Foxp3− T cells (center-right), and CD8+CD4− T cells (right) with representative FCS plots from sLNs included on the left. Plots (FCS) that represent spleen, mLN, and thymus data are included in **Supplementary Figures S3B–D**. Cells from spleens, sLNs, or mLNs of Sin3a^−/−^Foxp3^YFPcre^ mice or controls were treated with PMA, ionomycin, and Brefeldin A for 3 h *in vitro*, then CD45+CD4+FoxP3+ (**D**, left) Tregs and CD45+CD4+Foxp3− T cells (**D**, right) were evaluated for cytokine production by flow cytometry. Representative cytokine FCS plots from Tregs (**Supplementary Figure S11A**) and CD4+ T cells (**Supplementary Figure S11B**) are included in the **Supplementary Materials**. Cytokine and chemokine receptors differentially expressed by Sin3a^−/−^ versus Foxp3^YFPcre^ control CD25+Foxp3+-sorted Tregs by RNAseq, depicted by heatmap with log_2_ fold change (FC) in the left column with the color scale on the right **(E)**. Symbols representing statistical significance are as follows: *p ≤ 0.05; **p ≤ 0.01; ***p ≤ 0.001; and ****p ≤ 0.0001.

Sin3a deletion in Tregs resulted in the development of autoimmunity. In addition to the marked reduction in FoxP3 and peripheral Treg numbers, loss of function was also evaluated. Tregs that lack Sin3a were therefore tested for their ability to suppress the proliferation of lymphoid responder cells *ex vivo*. Sin3a^−/−^ Tregs from Foxp3^cre^, TAM-induced Foxp3^cre^, and CD4^cre^ backgrounds were all severely impaired in their abilities to suppress the proliferation of CD4+, CD8+, and total SLO responder cells (**Figure 5A**). Treatment with the Sin3a inhibitor, Selamectin, also reduced CTR Treg suppressive function in a dose-dependent manner (**Supplementary Figure S13**). In addition, Sin3a^−/−^ Tregs from peripheral lymphoid sights had higher CD69 activation in FoxP3cre (**Figure 5B**), CD4^cre^ (**Supplementary Figure S4B**), and TAM-induced FoxP3cre (**Supplementary Figure S7B**). Likewise, a marker of cell division, Ki-67, was increased in splenic Tregs of Sin3a^−/−^Foxp3^cre^ (**Figure 5C**) and SLOs of Sin3a^−/−^TAM (**Supplementary Figure S7B**). In addition, transcription of the Ki-67 gene, *mki67*, was increased in peripheral Tregs lacking Sin3a (**Supplementary Figure S16B**). These data show that without Sin3a, Tregs are activated but lack suppressive function.

In the near absence of suppressive Tregs within Sin3a^−/−^Foxp3^cre^ mice, CD4+ T cells in peripheral lymphoid tissues had higher CD69+ activation than those in controls (**Figure 5B**). This was also true of CD4+ cells within the CD4^cre^ (**Supplementary Figure S4C**) and TAM-induced Foxp3^cre^ (**Supplementary Figure S7C**) backgrounds. Peripheral CD4+ and CD8+ cells in Sin3a^−/−^FoxP3^cre^ displayed more Ki-67 in the spleens and mLNs (**Figure 5C**). These results reveal that activated peripheral Tregs lacking Sin3a were unable to suppress effector T lymphocyte activation and proliferation.

To further examine the functional properties of Tregs lacking Sin3a, cytokine production was evaluated. Compared to controls, splenic Sin3a^−/−^ Tregs produced more granzyme B, interferon gamma (IFN-γ), and TNF-γ (**Figure 5D**), and transcription of *ifng* and *grzb* was increased, as determined by RNAseq (**Figure 5E**) and RT-qPCR (**Supplementary Figure S14A**). Many other cytokines were also increased in Sin3a^−/−^ Tregs (**Figure 5E**; **Supplementary Figure S16**), including interleukins 3, 4, 5, 7, 10, 13, 15, 21, and 22 (**Figure 5E**; **Supplementary Figure S16B**), as well as expression of perforin and GrzA (**Figure 4C**). Sin3a^−/−^ Tregs from the spleens and sLNs had increased CCR5, CCR6, and CCR7, while mLN Tregs had increased CCR2, CCR5, and CCR7 (**Supplementary Figure S7**). Levels of chemokine receptor transcripts in Foxp3+ Tregs varied, as CCR1, CCR2, and CCR5 had increased expression in Sin3a^−/−^ Tregs, while expression of CCR6, CCR7, CCR9, and CCR10 was reduced (**Figure 5E**; **Supplementary Figure S16B**). The observed differences in chemokine receptor expression and increases in cytokine production, especially that of IFN-γ, support that residual peripheral Sin3a^−/−^ Tregs display hyperactivated, proinflammatory features.

Along with increased cytokine production in Tregs, CD4+ (**Figure 5D**) and CD8+ (**Supplementary Figure S11C**) T cells from the same tissues produced higher cytokine levels. CD4+ T cells from spleens and sLNs of Sin3a^−/−^ mice produced more GrzB and IFN-γ, and the spleens also produced more TNF-γ (**Figure 5D**). CD8+ T cells from SLOs of Sin3a^−/−^ mice produced more GrzB and IFN-γ, while CD8+ T cells from spleens and sLNs produced more TNF-γ (**Supplementary Figure S11C**). These results demonstrate an environment where activated and dividing peripheral T effectors are not being suppressed by hyperactivated and dysfunctional Sin3a^−/−^ Tregs.

### Sin3a helps maintain Foxp3 protein stability in Treg cells

**Figure 6:**
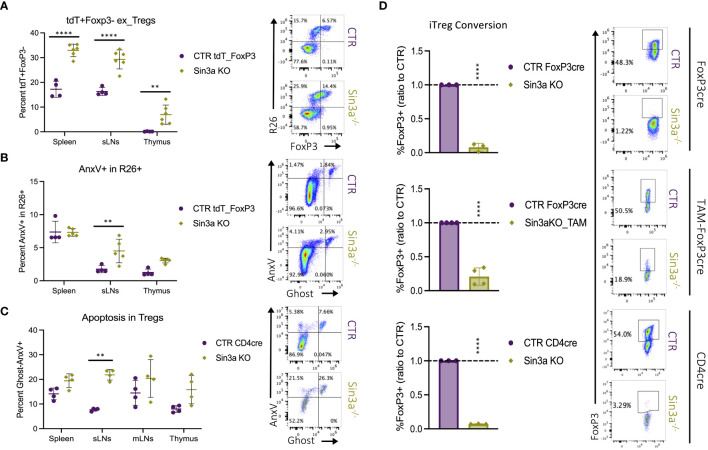
The fate of peripheral Tregs lacking Sin3a. The percentage of R26tdT+Foxp3− (exTregs) within CD45+CD4+ cells in Sin3a^−/−^ or control mice **(A)** with representative.fcs plots of the sLNs included to the right. Diagram of the genetics and flow-cytometry analysis of Treg fate mapping using the R26^STOP^tdTomatoFoxp3*^YFPcre^* model (**Supplementary Figure S17A**). The same samples were assessed for apoptotic cell death via flow cytometry staining for annexin V (AnxV) and Ghost-viability dye within CD45+CD4+R26+ cells **(B)** with representative FCS plots of sLNs to the right. The percent AnxV+Ghost− apoptotic CD4+CD25+ cells from Sin3a^−/−^CD4^cre^ or control lymphoid organs determined by FACS **(C)** with representative FCS plots from sLNs to the right. FCS plots which represent spleen, mLNs, and thymus data are included in **Supplementary Figures S17B**, **C**. The ratio of percent Foxp3+ cells of KO to CTR following iTreg conversion **(D)** with Sin3a^−/−^Foxp3^cre^ (top), TAM-induced Sin3a^−/−^Foxp3^cre^ (middle), or Sin3a^−/−^CD4^cre^ (bottom) with corresponding controls, representative FCS plots included to the right. Symbols representing statistical significance are as follows: *p ≤ 0.05; **p ≤ 0.01; ***p ≤ 0.001; and ****p ≤ 0.0001.

Sin3a^−/−^Foxp3^cre^ mice had drastically depleted Foxp3+ Treg populations in their SLOs, while thymic populations remained similar or even higher than those in controls. To assess whether the low numbers of peripheral Foxp3+ Tregs were caused by the loss of Foxp3, resulting in the generation of exTregs, we utilized a R26_tdT lineage tracing model, illustrated in **Supplementary Figure S17A**. When Foxp3^YFP-cre^R26tdT mice were compared to those with Sin3a deletion (Sin3a^flox/flox^Foxp3^YFP-cre^R26tdT), mice with Sin3a deletion had higher percentages of R26+Foxp3− exTregs in the SLOs and thymi (**Figure 6A**). These data show that the reduction of peripheral Foxp3+ Tregs in Sin3a^−/−^FoxP3^cre^ mice was at least partially due to the loss of Foxp3, resulting in an expanded exTreg population.

Apoptotic cell death was also assessed as a potential contributor to the depletion of peripheral Foxp3+ Tregs in Sin3a^−/−^ mice by FACS staining with annexin V (AnxV). Sin3a^−/−^ Tregs from the sLNs displayed increased levels of AnxV binding in both Sin3a^−/−^tdTR26Foxp3YFP^cre^ (**Figure 6B**) and Sin3a^−/−^CD4^cre^ (**Figure 6C**) mice. RNAseq analysis of peripheral Sin3a^−/−^ Tregs revealed many differentially expressed genes associated with the regulation of apoptosis. Proapoptotic factors such as caspases 3 and 12, Bak1, FasL, Apaf1, and cytochrome *c* were increased in transcript expression, while antiapoptotic factors like Bcl2, Noxa1, and Mcl1 were decreased (**Supplementary Figure S14**). There were, however, some pro-apoptotic factors with deceased expression, one of which was Bcl2l11 (aka Bim), as verified by RT-qPCR (**Supplementary Figure S14A**). This is of interest because Mcl1 and Bim have been described as the dominant anti- and proapoptotic factors (respectively) in Tregs (57, 58). Overall, proapoptotic factors with increased expression outnumbered those with decreased expression, and vice versa. GESA-enriched DEG data illustrated a significant skew of expression variation in the p53 pathway (**Supplementary Figure S15P**) and ferroptosis (**Supplementary Figure S15O**). Thus, it is likely that one or more forms of programmed cell death contribute to peripheral depletion of Tregs, particularly in the sLNs of Sin3a^−/−^Foxp3^cre^ mice, in addition to exTreg formation.

In addition to exploring Foxp3 loss and apoptosis as reasons for the lack of peripheral Tregs in the absence of Sin3a, peripheral CD4+CD25− cells were tested for the ability to induce Foxp3 production following IL-2, TGF-β, and CD3/28 stimulation. Deletion of Sin3a from the Foxp3^cre^, TAM-induced Foxp3^cre^, and CD4^cre^ backgrounds rendered CD4+CD25− cells practically incapable of Foxp3 induction (**Figure 6D**; **Supplementary Figure S18A**). Furthermore, selamectin treatment of CTR CD4+CD25− cells reduced Foxp3 induction in a dose-dependent manner (**Supplementary Figure S18B**). Together, these data suggest that peripheral reduction of Foxp3+ Tregs in Sin3a^−/−^ mice is due to the combinatorial effects of programmed cell death, Foxp3 instability, and impaired *Foxp3* induction.

### Sin3a supports Foxp3 expression within Treg cells

**Figure 7:**
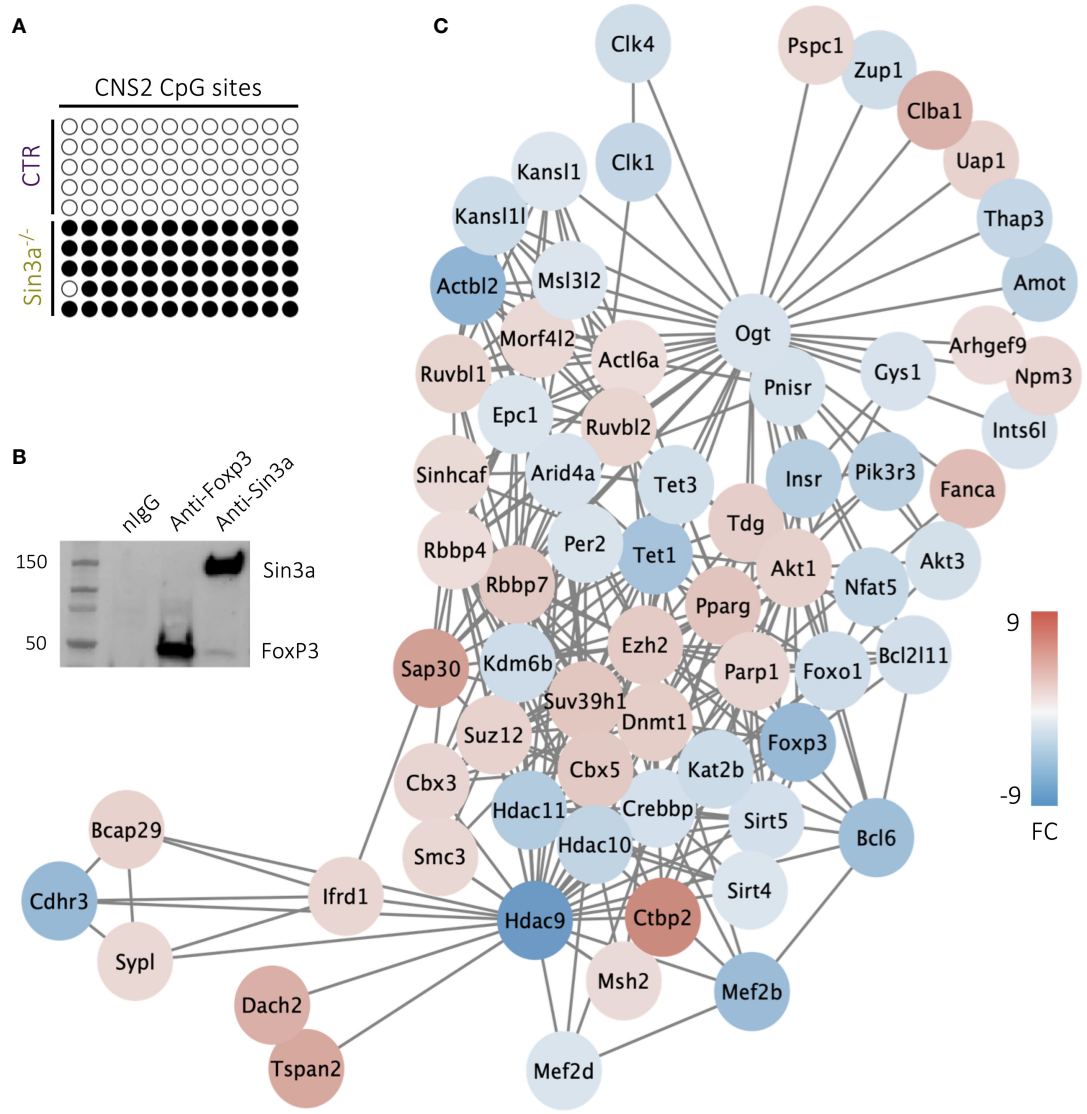
CpG site methylation of the CNS2 promoter region of the *foxp3* gene in CD25+Foxp3+ Tregs **(A)**, where empty circles represent nonmethylated CpG and filled circles represent methylated CpG,and each row depicts an individual sample. Individual samples represent pooled DNA isolated from sorted CD25+Foxp3+ Tregs from SLOs of Sin3a^−/−^ or Foxp3^YFPcre^ mice. Co-IP of Sin3a and FoxP3 in transfected 293T cells **(B)**. DEGs determined by RNAseq of Sin3a^−/−^ versus Foxp3^cre^ control CD25+Foxp3+ Tregs that are associated with Tet1 within the STRING network are displayed as circular nodes colored on a scale according to log_2_ fold change (FC) and organized using Cytoscape MSC clustering **(C)**. RNAseq was performed using six biological replicates per group, three males and three females.

Among the four *Foxp3* regulatory regions, CNS2 (aka TSDR) has been specifically associated with the stability of *Foxp3* expression (59). We questioned whether CNS2 accessibility due to CpG methylation was altered in Tregs lacking Sin3a, thereby destabilizing *Foxp3* expression. Bisulfite conversion and sequencing of the CNS2 region of Foxp3+ Tregs revealed that in the absence of Sin3a, CNS2 remained nearly completely methylated while all CpG sites of the control CNS2 were demethylated (**Figure 7A**). The stark difference in CpG methylation of CNS2 between Sin3a^−/−^ and control Foxp3+ Tregs suggests a massive alteration in methyltransferase activity. Tet enzymes (Tet1, Tet2, and Tet3) are DNA methyltransferases that contribute to the demethylation of *Foxp3* regulatory regions (15, 60). RNAseq analysis showed that Tet1 and Tet3, but not Tet2, had reduced expression in Sin3a^−/−^ Tregs (**Figure 7C**), which was confirmed by RT-qPCR (**Supplementary Figure 14B**). The expression of Dmnt1, a methyltransferase known to methylate CNS2 (15), was increased in Sin3a^−/−^ Tregs (**Figure 7C**).

ChIP-seq analysis (ChIP-Atlas) showed colocalization of FoxP3 with Sin3a in Tregs with H-H peak intensities with a STRING binding score of 500 (61), suggesting these molecules may interact as regulatory cofactors. Upon the co-IP pulldown of Sin3a and Foxp3, Sin3a did indeed interact with Foxp3 (**Figure 7B**), though there remains a possibility that other molecules contribute to this interaction, as benzonase treatment was not used during protein preparation. These data suggest Foxp3 and Sin3a may contribute to the regulation of the Treg transcriptional profile via direct or indirect interaction (likely accompanied by other cofactors), consistent with our overall data showing that Sin3a is required for the transcriptional regulation and maintenance of Foxp3 expression and Treg suppressive function.

## Discussion

Foxp3+ Treg cells have an essential role in immune homeostasis as a result of two main functions: restriction of T effector cell activity and maintenance of self-tolerance. Both functions were impaired by Sin3a depletion in Foxp3+ Tregs. The pathology associated with severe and fatal autoimmunity observed in mice with conditional deletion of Sin3a in Foxp3+ Tregs closely resembled that of Scurfy mice, which have dysfunctional Tregs due to a frameshift mutation of Foxp3 (5, 7). Treg suppression of T-cell proliferation *in vitro* was largely abrogated by the Sin3a deletion (**Figure 5A**; **Supplementary Figure S13**). In addition, mice with Sin3a deletion in Foxp3+ Tregs had fewer naïve and more activated (CD69+) effector T lymphocytes (**Figure 5C**; **Supplementary Figure S3E**), suggesting a lack of suppressive constraint by Tregs. Failure of self-tolerance was evident in Sin3a^−/−^Foxp3^cre^ mice by systemic tissue damage (**Figure 1**; **Supplementary Figure S1**) and abundant autoantibody generation (**Figure 1E**). Thus, Sin3a is crucial for Treg immunosuppressive functions.

When Sin3a deletion in Foxp3+ Tregs was induced following normal pre- and postnatal development (**Supplementary Figure S5**), evidence of autoimmunity developed in sLNs and lungs (**Supplementary Figures S6A, B**). Though the percentage of Foxp3+ Tregs was unchanged (**Supplementary Figure S7A**), Foxp3 protein levels decreased (**Supplementary Figure S7A**), as did Treg suppressive function (**Figure 5A**), and sLN of these mice had less naïve and more activated (CD69+) effector T cells (**Supplementary Figures S7C, E**). One explanation for why the induction of Sin3a deletion did not provoke the severity of autoimmunity present in Sin3a^−/−^Foxp3^cre^ is that the role of Sin3a within Foxp3+ Tregs is of vital importance during fetal and postnatal development. It is also likely the case that TAM-induced Sin3a deletion does not achieve full deletion in all Foxp3+ Tregs. Factors that could contribute to this include the variability in tamoxifen uptake and processing among cell and tissue types. Continued thymic production of Foxp3+ Tregs subsequent to tamoxifen treatment may also contribute to a mixture of dysfunctional Sin3a^−/−^ Tregs and functional Sin3a+ Tregs. Consistent with this, female heterozygous Sin3a^−/+^Foxp3^cre^ mice did not develop fatal autoimmunity.

While the vast majority of our data supports that Foxp3 is reduced in the absence of Sin3a, the percentage of Foxp3+ Tregs within the CD4+ population was greatly increased in Sin3a^−/−^CD4^cre^ SLOs (**Supplementary Figure S4A**), as also noted in the recent study by Perucho et al. (56). However, the interpretation of data regarding FoxP3+ Tregs from a CD4^cre^ KO background should consider the impact of KO within the other T lymphocyte lineages. Sin3a deletion from the CD4^cre^ background results in Sin3a deletion from that and all subsequent T lymphocyte lineages, including CD8 T, NK T, Th17, and Treg cells. Sin3a is present within such a T-cell lineage, and thus its absence would likely impact each of these cell types. Hence, it is difficult to draw conclusions regarding Tregs with respect to other CD4-lineage T lymphocyte populations in CD4^cre^ KO mice. Rather, Sin3a^−/−^Foxp3^cre^ mice were utilized for the analysis of cellular populations, where the percentage of Foxp3+ cells (**Figures 2C**, **3B**) and the number of Foxp3+ Tregs (**Figure 2F**) were depleted. Our data are supported by the reduction of Foxp3 protein (**Figures 2H, J**) and transcript expression (**Figures 2K**, **4C**; **Supplementary Figures S14**, **S15B**) and the increase of exTregs (**Figure 6A**) present in Sin3a^−/−^ mice. Together, these provide a consensus that Foxp3 is indeed diminished in Sin3a-deficent Tregs.

Phenotypic changes in the CD4-lineage populations of Sin3a^−/−^CD4^cre^ mice likely explain why they did not develop fatal autoimmunity like Sin3a^−/−^Foxp3^cre^ mice despite having dysfunctional Tregs. Evidence of this within the CD4 and CD8 T-cell populations is included in the **Supplementary Materials**, where the CD4 and CD8 T-cell populations shifted to increased effector and memory phenotypes and reduced naïve T cells (**Supplementary Figure S4D**). The CD4 T cells from Sin3a^−/−^CD4^cre^ mice had higher percentages of Ki-67+ division and CD69+ activation (**Supplementary Figure S4C**) compared to controls. Overall, the CD8 and especially the CD4 T cells in Sin3a^−/−^CD4^cre^ mice mainly showed an activated effector phenotype, similar to Sin3a^−/−^Foxp3^cre^ CD4 T-cell populations and consistent with reduced suppression by dysfunctional Tregs. However, unlike in Sin3a^−/−^FoxP3^cre^, the percentages (**Supplementary Figure S4A**) and numbers (**Supplementary Figure S4G**) of CD4+ and CD8+ T cells in the SLOs of Sin3a^−/−^CD4^cre^ mice were greatly reduced, providing an explanation as to why these mice do not develop fatal autoimmunity.

An inconsistency remains, however, that Sin3a^−/−^CD4^cre^ mice maintained the ability to induce Foxp3 expression peripherally (**Supplementary Figure S4A**) while Sin3a^−/−^Foxp3^cre^ (**Figures 2C, F**) did not. Induction of *Foxp3* expression is facilitated through the CNS1 enhancer, which is dispensable for *Foxp3* expression stability (62). Demethylation of CNS1 can occur via Dnmt3 (Dnmt3a and/or Dnmt3b) activity (13, 63), which has not been linked to Sin3a and is not differentially expressed in Tregs lacking Sin3a (**Supplementary Figure S16A**). Thus, in the absence of Sin3a, CNS2 remains methylated (**Figure 7A**), and Dnmt3 demethylation of CNS1 allows induction but not stability of *Foxp3*. This would presumably occur in Tregs of both CD4^cre^ and Foxp3^cre^ backgrounds, the difference in Sin3a^−/−^CD4^cre^ mice being the reduction and phenotypic variation of T-cell populations. This corresponds with the concept that Sin3a is important to the maintenance of *Foxp3* expression through CNS2 demethylation.

Hypomethylation of the *Foxp3* enhancers CNS1 and CNS2 is required for optimal Foxp3 expression initiation and stability. The role of methyltransferase Dnmt1 in CpG methylation of the *Foxp3* enhancers is well established (13, 52). Previous reports in other cell types have found that Sin3a regulates CpG methylation indirectly by DNMT1 repression and TET1 activation (38, 64). In addition, “genome-wide” Dnmt1-mediated CpG methylation occurred in the absence of Sin3a (38). In Tregs lacking Sin3a, expression of Dnmt1 was dramatically increased, along with increased expression of two Dnmt1 (**Figure 7C**) cofactors, PCNA and UHRF1 (**Supplementary Figure S16B**). While the increase of Dnmt1and its cofactors likely contribute to the hypermethylation of CNS2, its continued state of hypermethylation suggests another layer of dysregulation. During T lymphocyte hematopoiesis the CNS2 region is methylated in CD4 T cells and must undergo CpG demethylation upon Treg lineage commitment (65). Therefore, a failure in *de novo* CpG demethylation may also be involved in Tregs lacking Sin3a.

**Figure 8:**
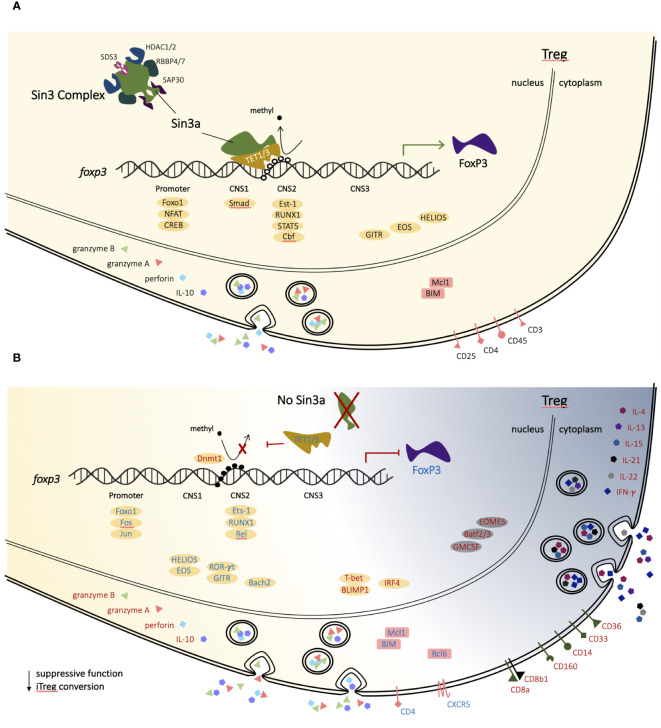
Proposed mechanism of Sin3a in T-regulatory cells. In a normal T-regulatory (Treg) cell, Sin3a facilitates CpG demethylation of CNS2 (TSDR) of the *Foxp3* gene via TET1 and/or TET3, thereby supporting the stability of *FoxP3* expression and lineage-specific factors **(A)**. In T-regulatory cells lacking Sin3a CpG demethylation of the *Foxp3* enhancer region, CNS2 does not occur, resulting in *Foxp3* depletion and Treg dysregulation **(B)**. Treg-associated transcription factors are depicted as yellow ovals and backgrounds, while those associated with alternate lymphoid lineages are represented as grey ovals and backgrounds. Genes with enhanced expression in the absence of Sin3a are represented by red text, while those with reduced expression are represented by blue text.

Demethylation of *Foxp3* enhancers occurs predominantly through the actions of Tet enzymes (15, 16). Tet2 and Tet3 are required for Treg stability and function through CpG demethylation of CNS2 (18, 19, 66, 67). Both Tet1 and Tet3 have a conserved single-helix Sin3a-interacting domain (SID) (68), which interacts with the PAH1 domain of Sin3a (40, 69–72). Tet1 and Tet3 both have DNA-binding ability (73, 74), whereas Tet2 lacks a SID, though there was a recent report of a Sin3a/Tet2 interaction (75). Tet2 was previously implicated in the demethylation of CNS2 while involved with another large protein regulatory complex (76), but the evidence supporting Sin3a/Tet1 and Sin3a/Tet3 outweighs that of Sin3a/Tet2 in contributing to CNS2 demethylation. Hence, we propose a mechanistic model (which needs validation) in which Sin3a-mediated Tet1 and Tet3 activity are critical for sustained *Foxp3* expression by CpG demethylation of CNS2 (**Figure 8A**).

RNAseq analysis of Foxp3+ Tregs (Sin3a^−/−^ vs. Foxp3^cre^ CTR) revealed vast differential expression of genes spanning diverse functional associations. Significantly, the Sin3a deletion affected genes specifically associated with the Treg phenotype. In the absence of Sin3a, expression of markers important to Tregs, including CD4, GITR, Helios, and Foxp3, was reduced, as were transcription factors involved in *Foxp3* activation and phenotype (Runx1, Est-1, Rel, Jun, Fos, Bach2, and Foxo1), whereas factors associated with eTregs, such as Blimp1, IRF4, GrzB, Prf1, and IL-10, had increased expression. In addition, there were many genes typically expressed by other immune cells, lymphoid and myeloid, that were increased in Sin3a-deficient Tregs, including T-bet, CD8a, and IFN-γ (**Figure 8B**). Thus, Sin3a is crucial to the regulation of the transcriptional profile of Foxp3+ Tregs. This conclusion is strengthened by the cooccurrence of DEGs downregulated in Sin3a^−/−^ Tregs and the ATAC-accessible genes of Tregs (**Table 1**). Sin3a likely acts synergistically with Foxp3 to achieve Treg transcriptional identity. Most importantly, Sin3a supports Treg identity and function through *Foxp3* expression stability via CNS2 demethylation.

The coregulator, Sin3a, plays a pivotal role in the regulation of transcriptional expression within Foxp3+ Tregs. This regulation spans a vast and diverse variety of functional networks, including those intimately linked to T-regulatory cell phenotypes. A central component of this was the extreme reduction of Foxp3 in Tregs that lacked Sin3a and the reduction of peripheral Foxp3+Tregs in Sin3s^−/−^Foxp3^cre^ mice. This peripheral depletion of Foxp3+ Tregs was likely caused by the loss of Foxp3 expression and ex-Treg formation due to CNS2 CpG-methylation, along with cell death, as seen particularly in sLNs. Sin3a deletion from Foxp3+ Tregs rendered the cells hyperactivated, proinflammatory, and lacking suppressive function. Hence, Sin3a is crucial for the stability of Foxp3, the control of effector activation, and the maintenance of Treg function.

## Data availability statement

The datasets presented in this study can be found in online repositories. The names of the repository/repositories and accession number(s) can be found below: GSE263830 (GEO).

## Ethics statement

Ethical approval was not required for the studies on humans in accordance with the local legislation and institutional requirements because only commercially available established cell lines were used. The animal study was approved by IACUC Children’s Hospital of Philadelphia. The study was conducted in accordance with the local legislation and institutional requirements.

## Author contributions

LC: Conceptualization, Data curation, Formal analysis, Investigation, Methodology, Visualization, Writing – original draft. TA: Conceptualization, Data curation, Formal analysis, Methodology, Project administration, Writing – review & editing. LW: Data curation, Formal analysis, Writing – review & editing. RH: Data curation, Writing – review & editing. AS: Data curation, Writing – review & editing. ED: Formal analysis, Writing – review & editing. WH: Conceptualization, Data curation, Formal analysis, Resources, Supervision, Writing – review & editing.
