# Supplementary material for: T-regulatory cells require Sin3a for stable expression of Foxp3
Source: Front Immunol. 2024 Aug 2;15:1444937. doi: 10.3389/fimmu.2024.1444937 (PMC11327135; doi:10.3389/fimmu.2024.1444937)
Supplement: Supplementary file 1 [file DataSheet_1.pdf]

Supplementary Data
Christensen *et al* 2024

Fig. 1

a

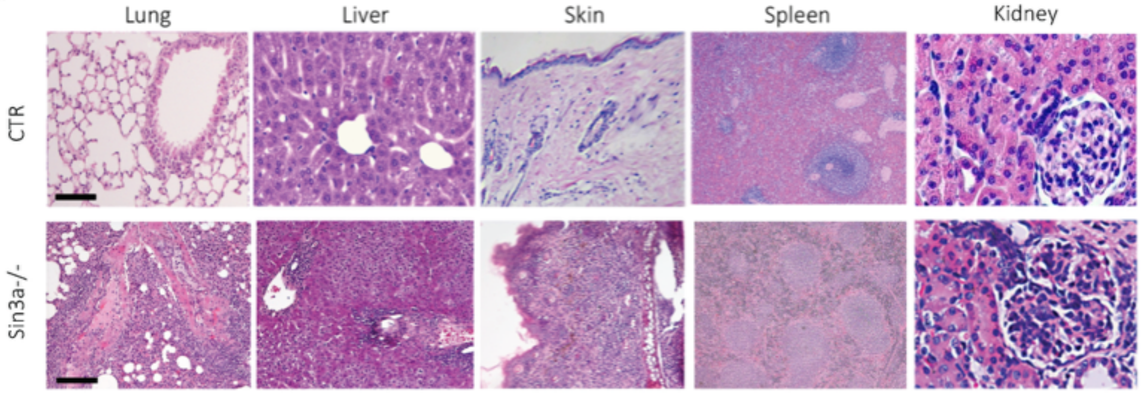

b

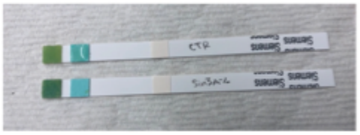

Mouse	Protein (mg/dL)	Glucose
CTR_FoxP3cre_u1	Trace	Trace
CTR_FoxP3cre_u3	Trace	Trace
Sin3aKO_u2	300+++	Trace
Sin3aKO_u4	300+++	Trace
Sin3aKO_u5	300+++	Trace

Supplementary Figure 1. (a) H&E-stained sections (100x) of lung, liver, skin, spleen, and kidney from Foxp3^{cre} CTR or Sin3a$^{-/-}$FoxP3^{Cre} mice. (b). Tissue section scale bars represent 100 µ. Levels of of protein and glucose in the urine of Sin3a$^{-/-}$FoxP3^{Cre} vs. control mice, with a representative image of the test strips used.

Fig. 2

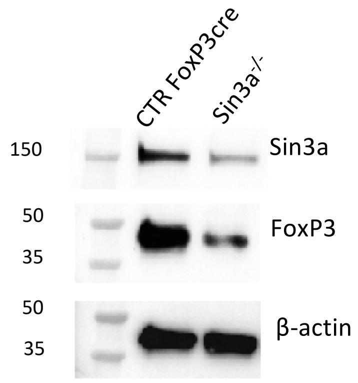

Supplementary Figure 2. Western blot of CD4+CD25+FoxP3+ sorted Tregs isolated from Sin3a-/-FoxP3cre or control FoxP3cre mice.

Fig. 3

a

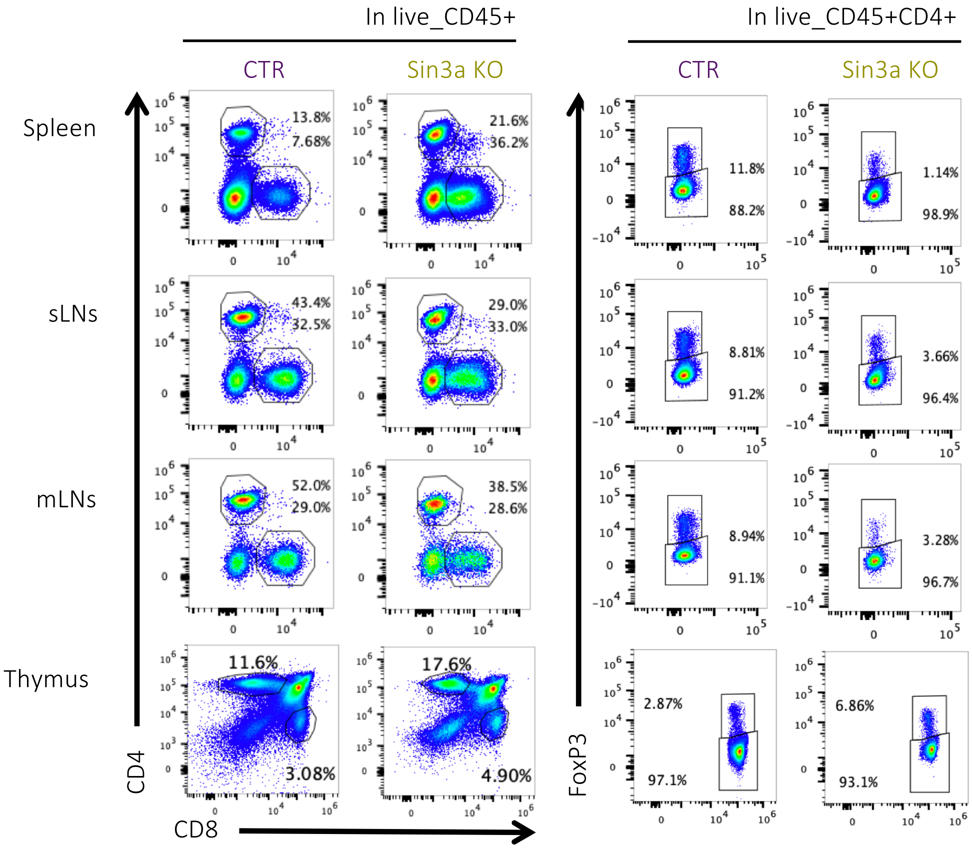

In live_CD45+
In live_CD45+CD4+
CTR
Sin3a KO
Spleen
sLNs
mLNs
Thymus
13.8%
7.68%
21.6%
36.2%
11.8%
88.2%
1.14%
98.9%
43.4%
32.5%
29.0%
33.0%
8.81%
91.2%
3.66%
96.4%
52.0%
29.0%
38.5%
28.6%
8.94%
91.1%
3.28%
96.7%
11.6%
3.08%
17.6%
4.90%
2.87%
97.1%
6.86%
93.1%
CD4
CD8
FoxP3

b

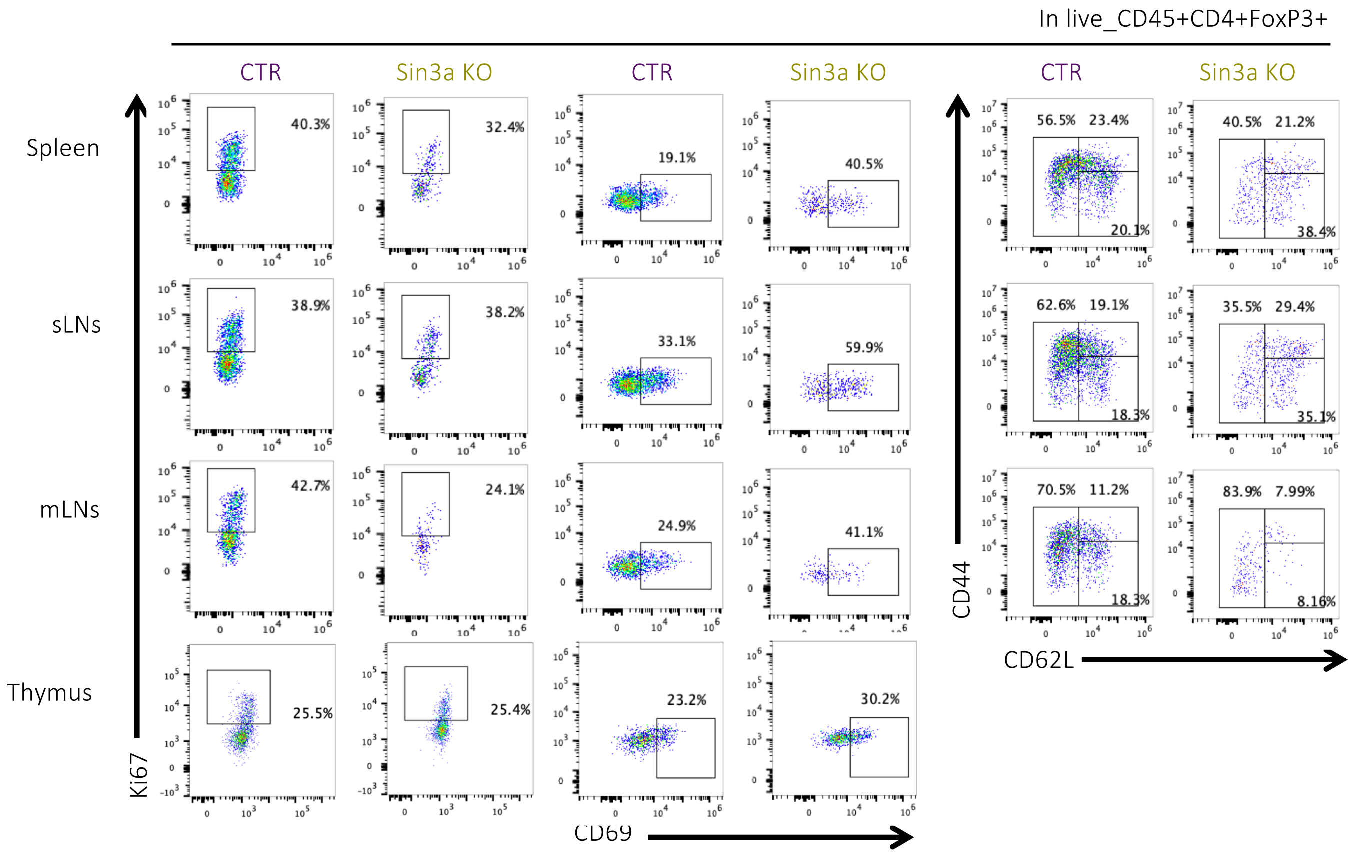

In live_CD45+CD4+FoxP3+
CTR
Sin3a KO
Spleen
sLNs
mLNs
Thymus
40.3%
32.4%
19.1%
40.5%
56.5%
23.4%
20.1%
40.5%
21.2%
38.4%
38.9%
38.2%
33.1%
59.9%
62.6%
19.1%
18.3%
35.5%
29.4%
35.1%
42.7%
24.1%
24.9%
41.1%
70.5%
11.2%
18.3%
83.9%
7.99%
8.16%
25.5%
25.4%
23.2%
30.2%
Ki67
CD69
CD44
CD62L

Fig. 3

c

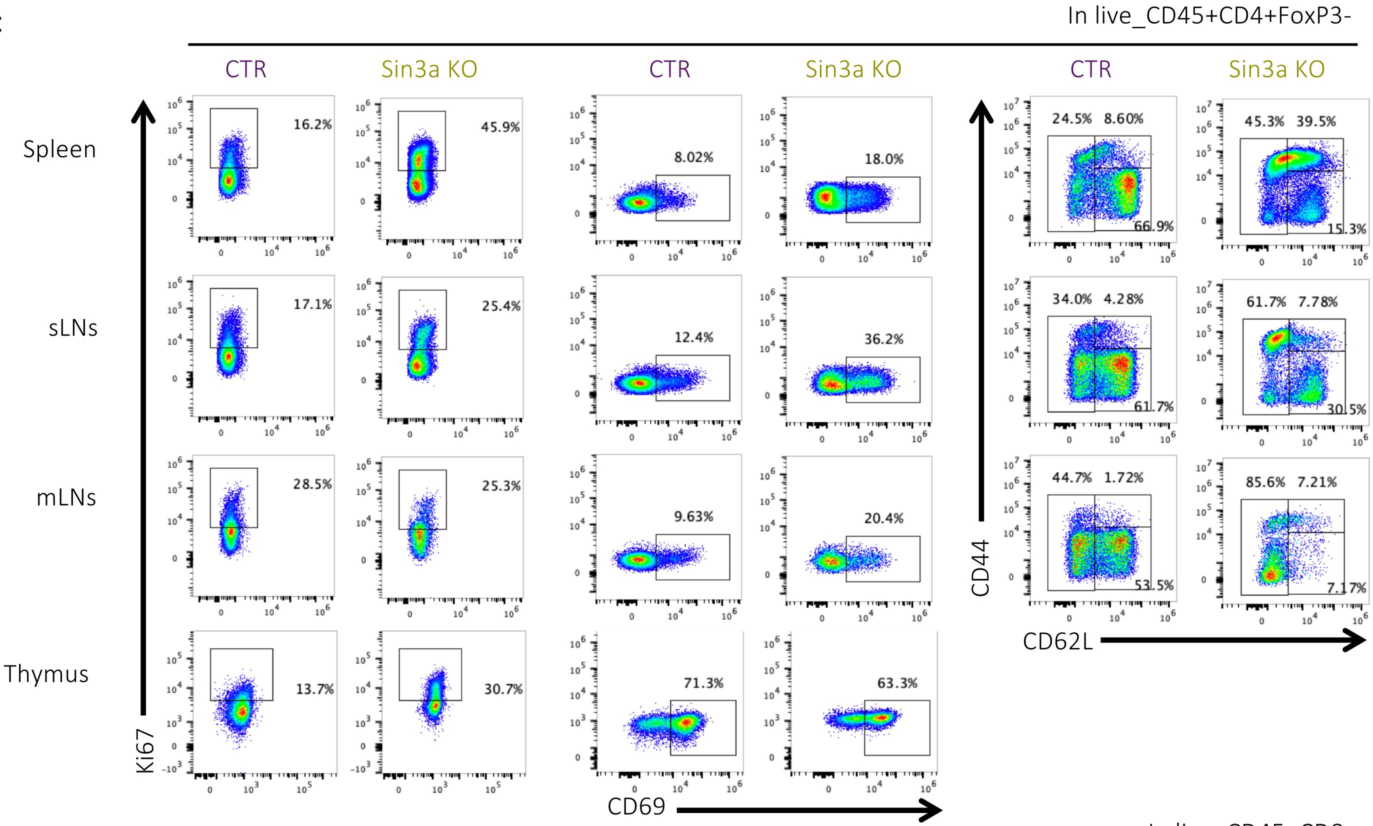

In live_CD45+CD4+FoxP3-
CTR
Sin3a KO
Spleen
sLNs
mLNs
Thymus
Ki67
CD69
CD44
CD62L
16.2%
45.9%
8.02%
18.0%
24.5% 8.60%
66.9%
45.3% 39.5%
15.3%
17.1%
25.4%
12.4%
36.2%
34.0% 4.28%
61.7%
61.7% 7.78%
30.5%
28.5%
25.3%
9.63%
20.4%
44.7% 1.72%
53.5%
85.6% 7.21%
7.17%
13.7%
30.7%
71.3%
63.3%

d

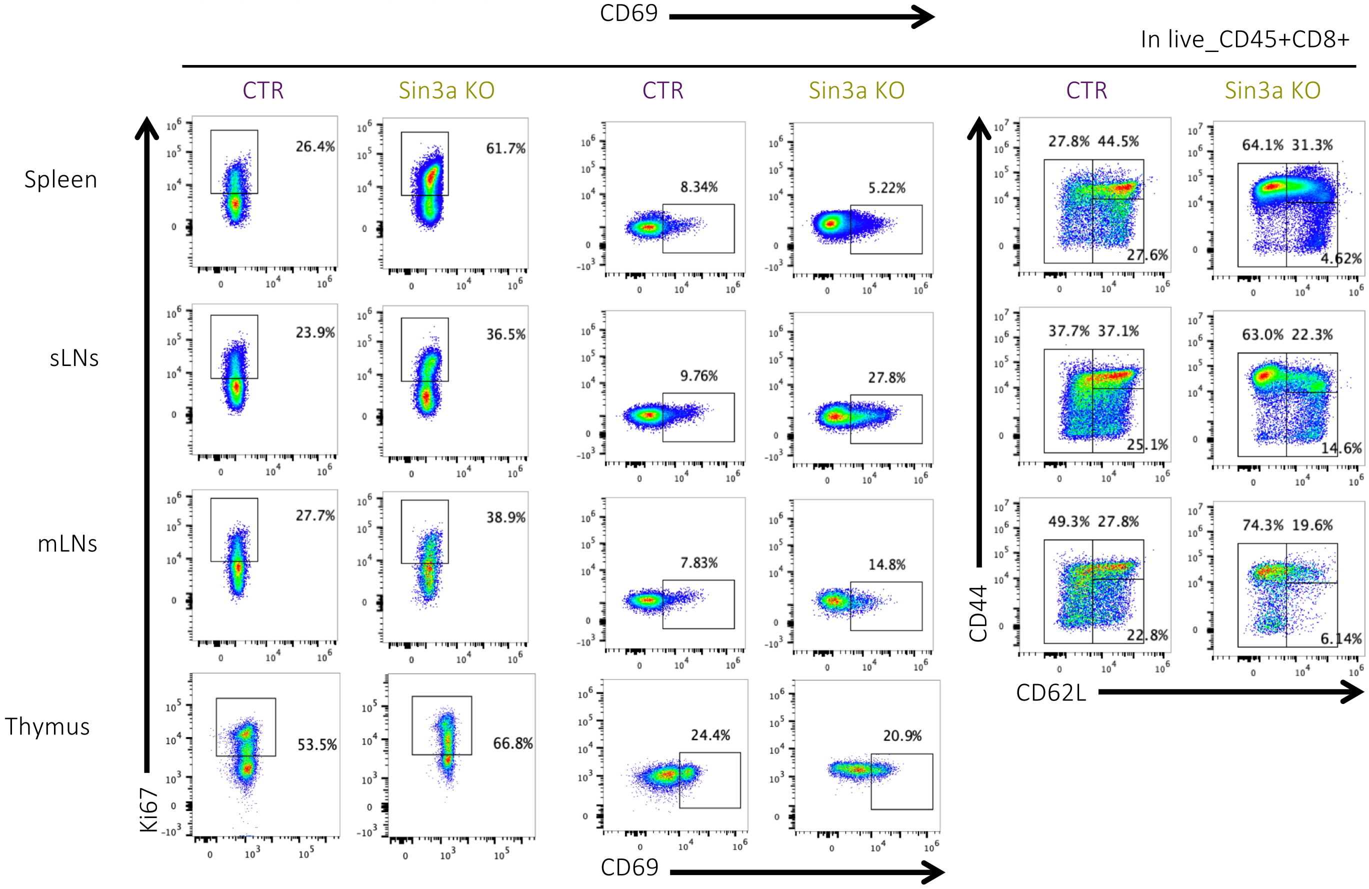

In live_CD45+CD8+
CTR
Sin3a KO
Spleen
sLNs
mLNs
Thymus
Ki67
CD69
CD44
CD62L
26.4%
61.7%
8.34%
5.22%
27.8% 44.5%
27.6%
64.1% 31.3%
4.62%
23.9%
36.5%
9.76%
27.8%
37.7% 37.1%
25.1%
63.0% 22.3%
14.6%
27.7%
38.9%
7.83%
14.8%
49.3% 27.8%
22.8%
74.3% 19.6%
6.14%
53.5%
66.8%
24.4%
20.9%

Fig. 3

e

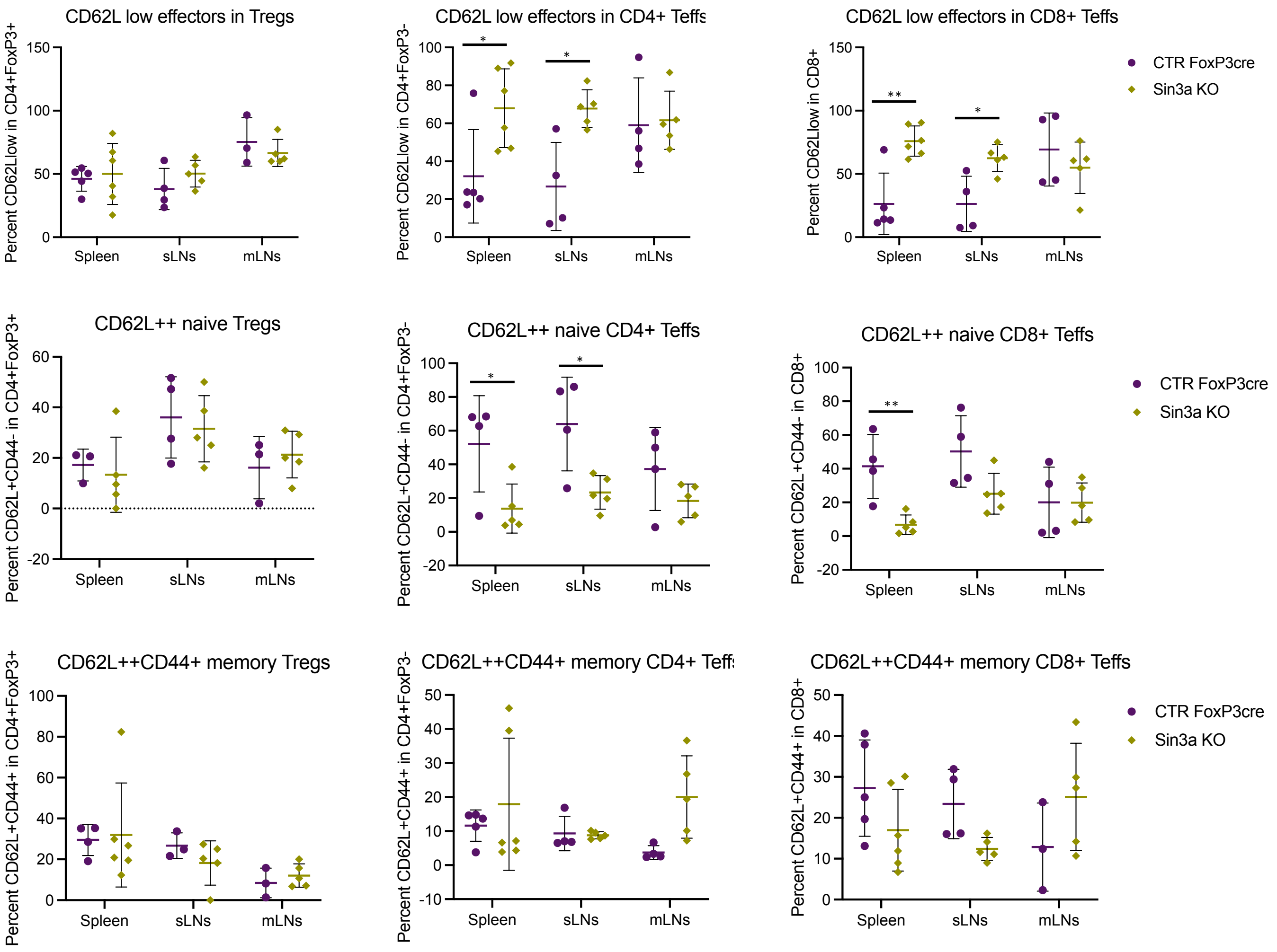

f

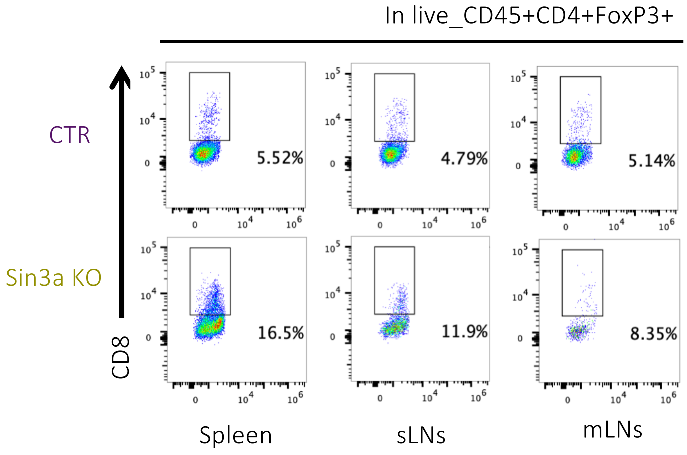

Supplementary Figure 3. The spleens, sLNs, MLNs, and thymi of Sin3a$^{-/-}$FoxP3^{Cre} mice compared to FoxP3^{cre} controls by flow cytometry. Representative .fcs plots for CD8+ T effector cells (live_CD45+CD8+), CD4+ T effector cells (live_CD45+CD4+FoxP3-), and Tregs (live_CD45+CD4+FoxP3+) within live_CD45+ (left) and live_CD45+CD4+ (right) populations (a). Images of representative .fcs plots for Ki67 (left), CD69 (center), and CD44_CD62L within Tregs (b), CD4+ Teffs (c), and CD8+ Teffs (d).

Fig. 4

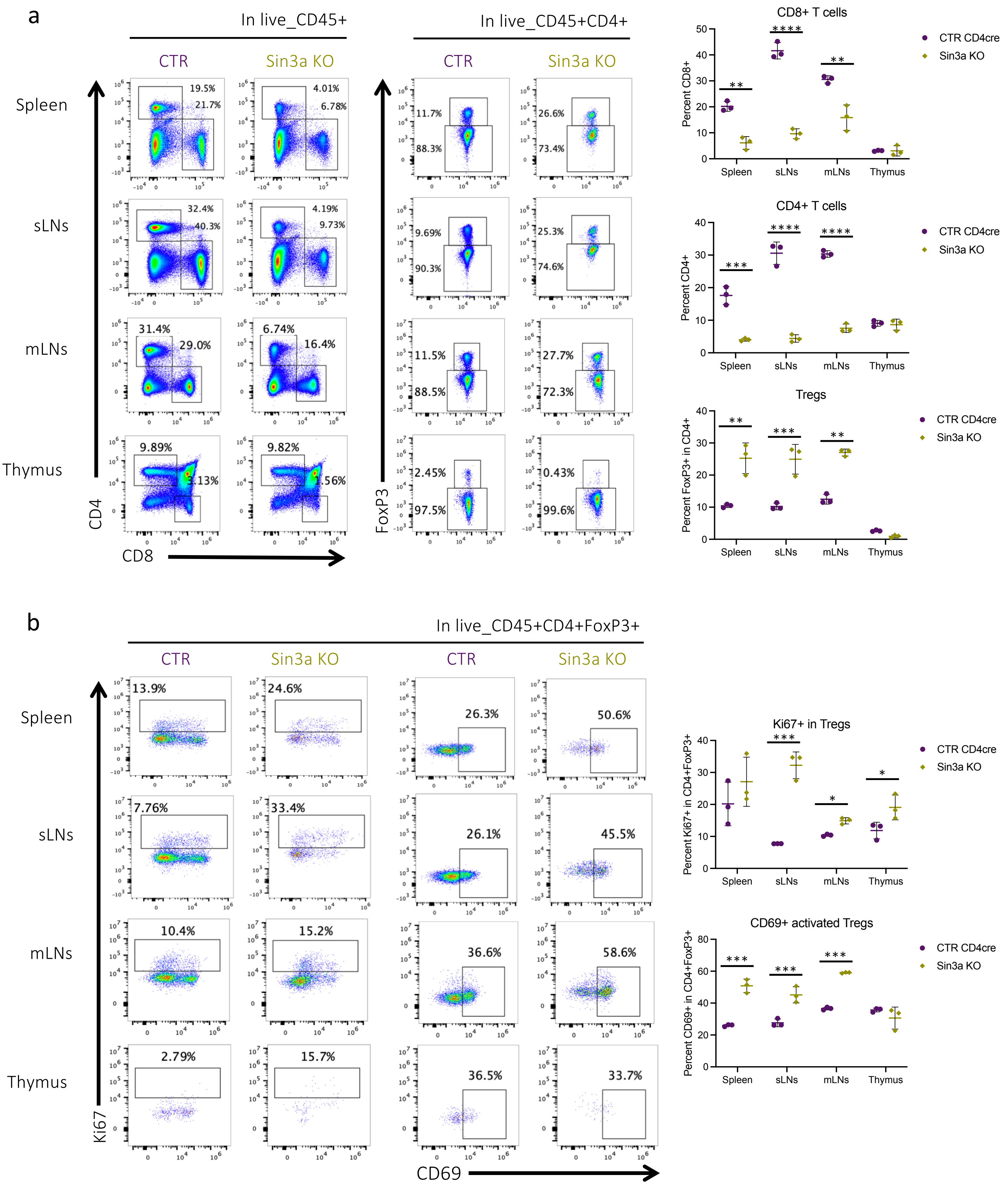

a
In live_CD45+
In live_CD45+CD4+
CTR
Sin3a KO
Spleen
sLNs
mLNs
Thymus
CD4
CD8
FoxP3
19.5%
21.7%
4.01%
6.78%
32.4%
40.3%
4.19%
9.73%
31.4%
29.0%
6.74%
16.4%
9.89%
3.13%
9.82%
1.56%
11.7%
88.3%
26.6%
73.4%
9.69%
90.3%
25.3%
74.6%
11.5%
88.5%
27.7%
72.3%
2.45%
97.5%
0.43%
99.6%
CD8+ T cells
Percent CD8+
CD4+ T cells
Percent CD4+
Tregs
Percent FoxP3+ in CD4+
CTR CD4cre
Sin3a KO
b
In live_CD45+CD4+FoxP3+
Ki67
CD69
13.9%
24.6%
26.3%
50.6%
7.76%
33.4%
26.1%
45.5%
10.4%
15.2%
36.6%
58.6%
2.79%
15.7%
36.5%
33.7%
Ki67+ in Tregs
Percent Ki67+ in CD4+FoxP3+
CD69+ activated Tregs
Percent CD69+ in CD4+FoxP3+

Fig. 4

c

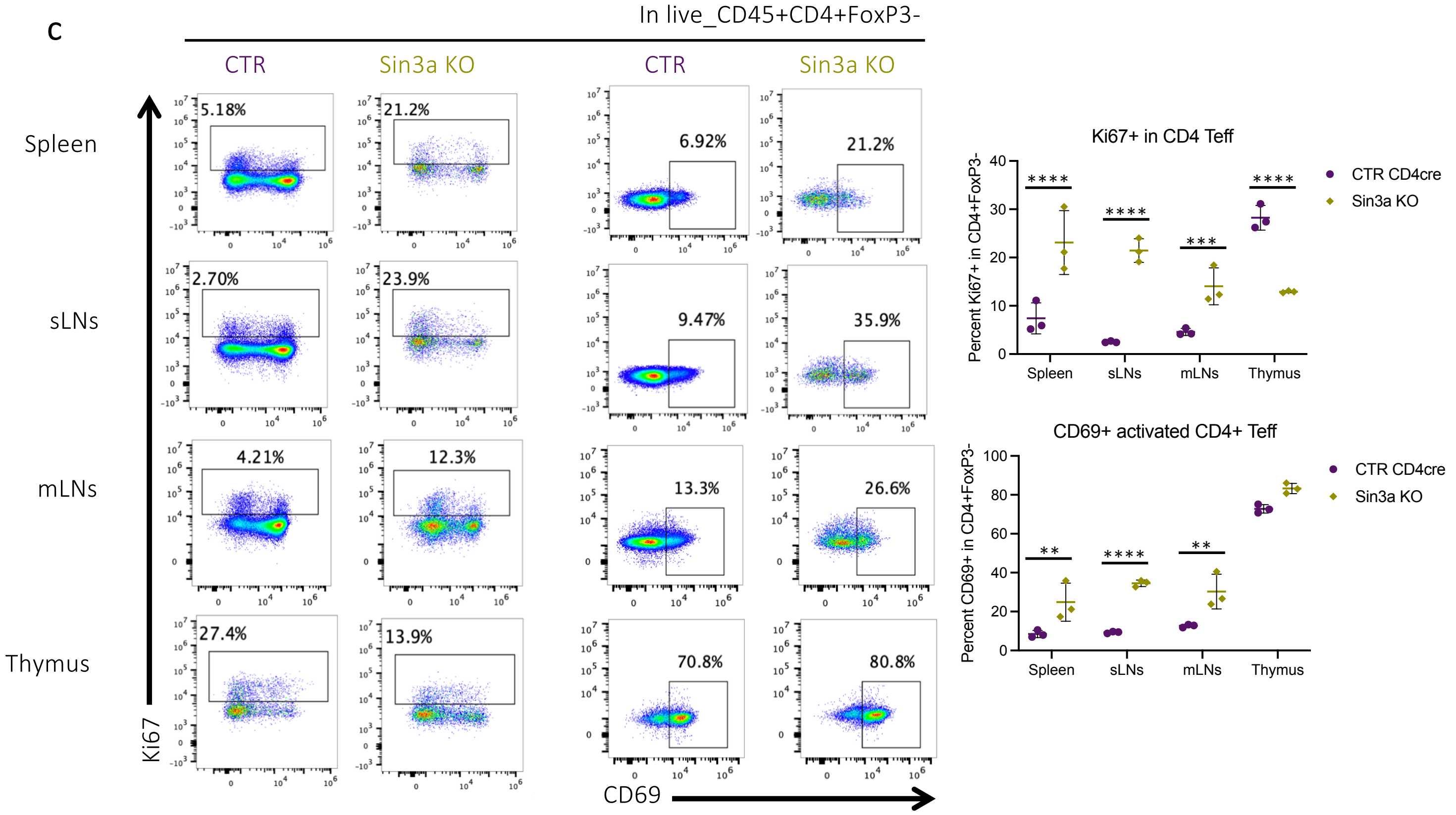
In live_CD45+CD4+FoxP3-
CTR
Sin3a KO
CTR
Sin3a KO
Spleen
sLNs
mLNs
Thymus
5.18%
21.2%
6.92%
21.2%
2.70%
23.9%
9.47%
35.9%
4.21%
12.3%
13.3%
26.6%
27.4%
13.9%
70.8%
80.8%
Ki67
CD69
Ki67+ in CD4 Teff
Percent Ki67+ in CD4+FoxP3-
CTR CD4cre
Sin3a KO
Spleen
sLNs
mLNs
Thymus
CD69+ activated CD4+ Teff
Percent CD69+ in CD4+FoxP3-

d

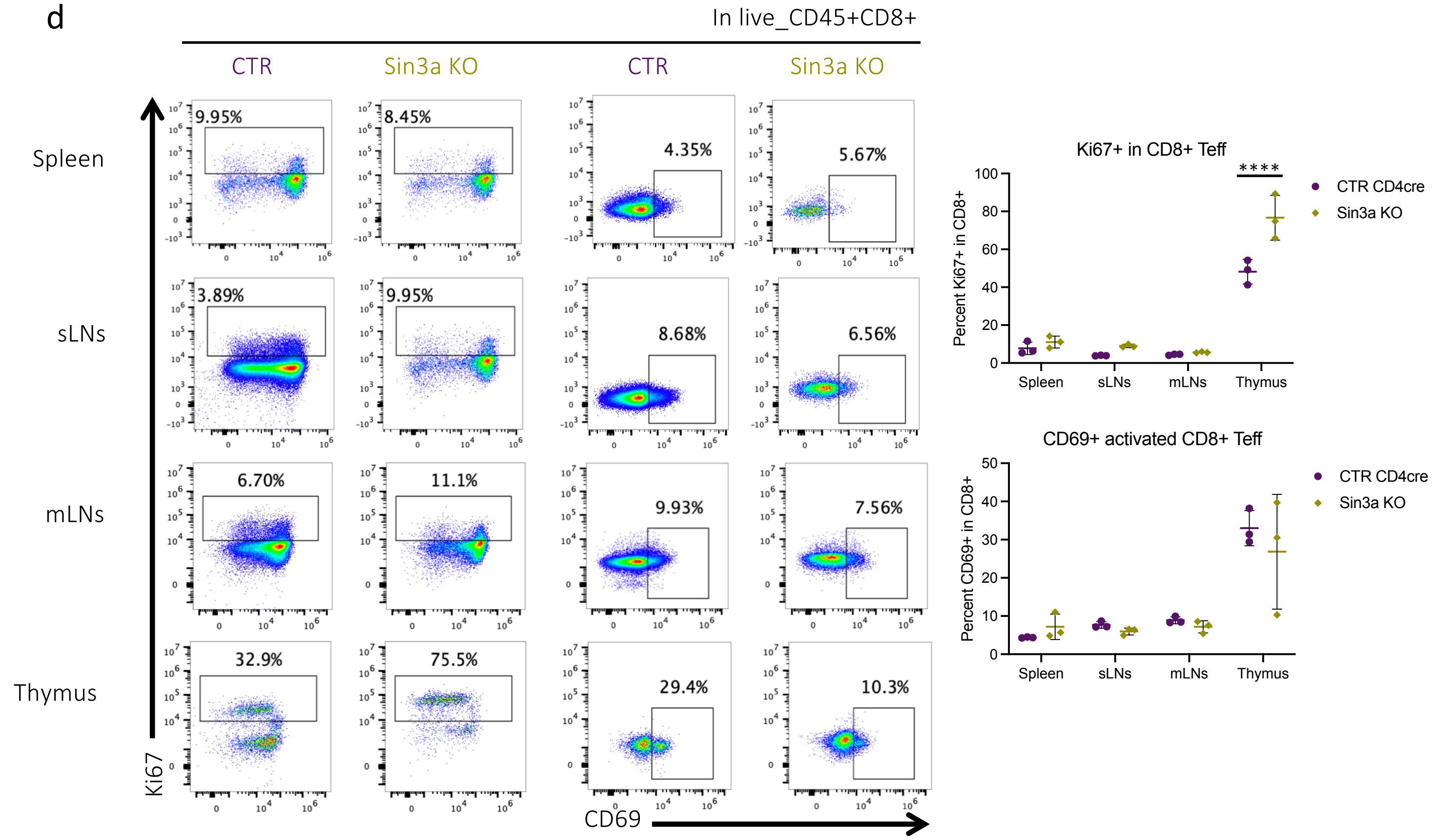
In live_CD45+CD8+
CTR
Sin3a KO
CTR
Sin3a KO
Spleen
sLNs
mLNs
Thymus
9.95%
8.45%
4.35%
5.67%
3.89%
9.95%
8.68%
6.56%
6.70%
11.1%
9.93%
7.56%
32.9%
75.5%
29.4%
10.3%
Ki67
CD69
Ki67+ in CD8+ Teff
Percent Ki67+ in CD8+
CTR CD4cre
Sin3a KO
Spleen
sLNs
mLNs
Thymus
CD69+ activated CD8+ Teff
Percent CD69+ in CD8+

Fig. 4

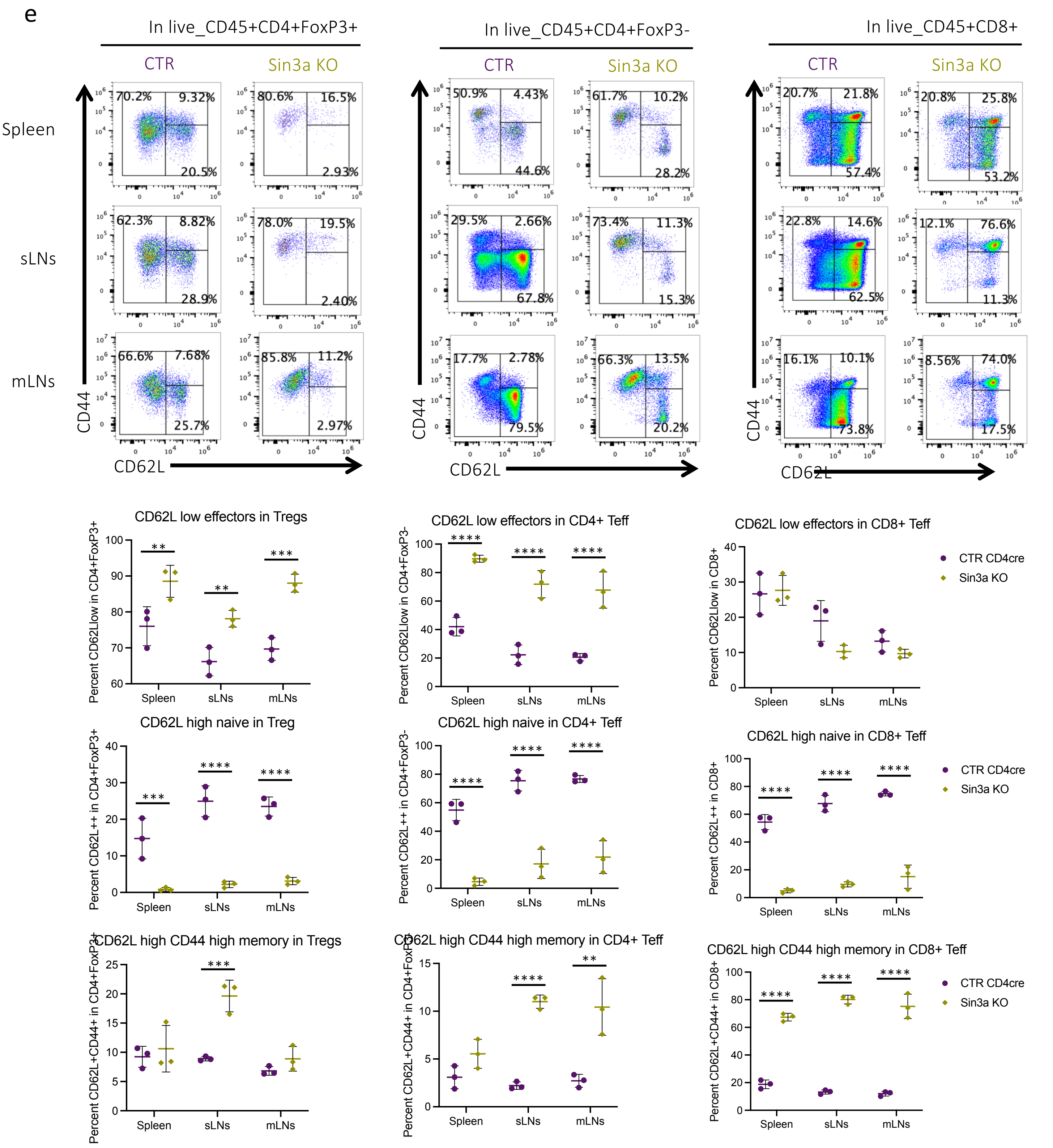
e
In live_CD45+CD4+FoxP3+
In live_CD45+CD4+FoxP3-
In live_CD45+CD8+
CTR
Sin3a KO
Spleen
sLNs
mLNs
CD44
CD62L
70.2%
9.32%
20.5%
80.6%
16.5%
2.93%
62.3%
8.82%
28.9%
78.0%
19.5%
2.40%
66.6%
7.68%
25.7%
85.8%
11.2%
2.97%
50.9%
4.43%
44.6%
61.7%
10.2%
28.2%
29.5%
2.66%
67.8%
73.4%
11.3%
15.3%
17.7%
2.78%
79.5%
66.3%
13.5%
20.2%
20.7%
21.8%
57.4%
20.8%
25.8%
53.2%
22.8%
14.6%
62.5%
12.1%
76.6%
11.3%
16.1%
10.1%
73.8%
8.56%
74.0%
17.5%
CD62L low effectors in Tregs
Percent CD62Llow in CD4+FoxP3+
CD62L low effectors in CD4+ Teff
Percent CD62Llow in CD4+FoxP3-
CD62L low effectors in CD8+ Teff
Percent CD62Llow in CD8+
CTR CD4cre
Sin3a KO
CD62L high naive in Treg
Percent CD62L++ in CD4+FoxP3+
CD62L high naive in CD4+ Teff
Percent CD62L++ in CD4+FoxP3-
CD62L high naive in CD8+ Teff
Percent CD62L++ in CD8+
CD62L high CD44 high memory in Tregs
Percent CD62L+CD44+ in CD4+FoxP3+
CD62L high CD44 high memory in CD4+ Teff
Percent CD62L+CD44+ in CD4+FoxP3-
CD62L high CD44 high memory in CD8+ Teff
Percent CD62L+CD44+ in CD8+
Spleen
sLNs
mLNs

Fig. 4

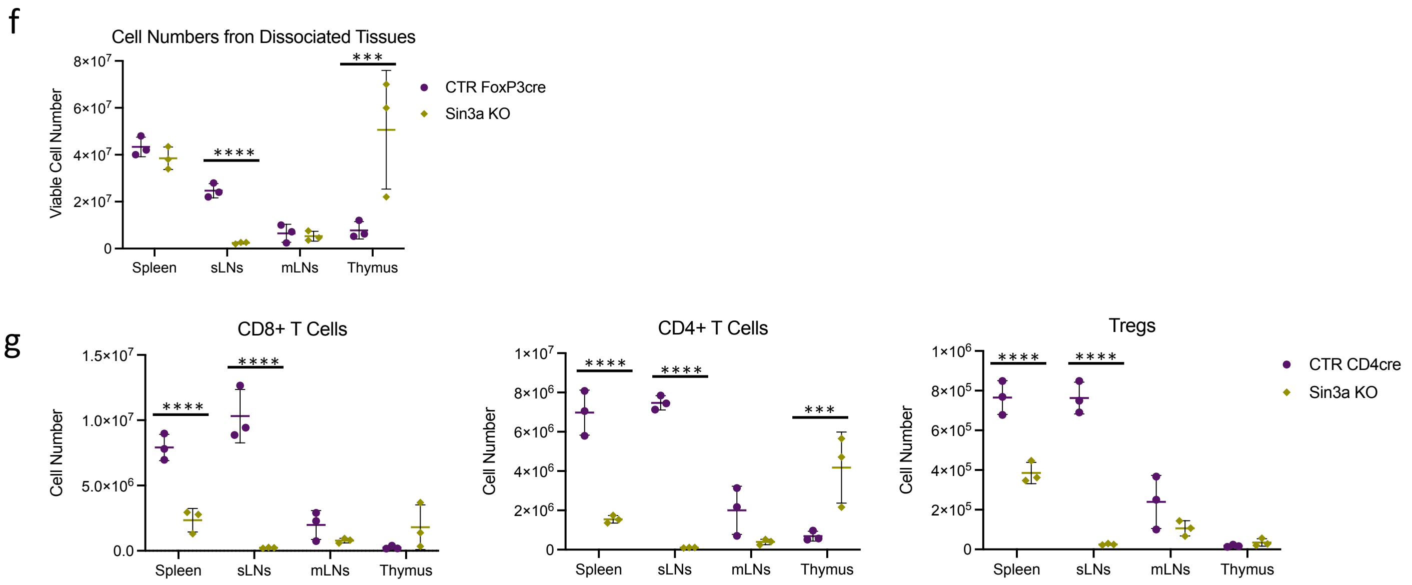

Supplementary Figure 4. The spleens, sLNs, MLNs, and thymi of Sin3a$^{-/-}$CD4^{Cre} mice compared to CD4^{cre} controls by flow cytometry. The percent of CD8+ T effector cells (live_CD45+CD8+), CD4+ T effector cells (live_CD45+CD4+FoxP3-), and Tregs (live_CD45+CD4+FoxP3+) are included in graphs (right) and representative .fcs plots within live_CD45+ (left) and live_CD45+CD4+ (right) populations in the center and left columns (a). Percentages of CD69+ and Ki67+ Tregs (b), CD4+ Teffs (c), and CD8+ Teffs (d) are graphed on the right and representative .fcs plots included on the left (% Ki67) and the center (% CD69). The effector (CD62Llo), naïve (CD62L++), and memory (CD62L++CD44+) subpopulations of Treg (left), CD4+ Teff (center), and CD8+ Teff (right) cell populations are graphed (below) and rep. images included (above) (e). The number of viable cells following tissue dissociation and single cell suspension, determined via hemocytometer (f), were then used together with population percentages (depicted in S2a) to determine the actual cell numbers of Tregs, CD4+ Teff, and CD8+ Teffs (g). This experiment was performed with 3 (male) biological replicates per group.

Fig. 5

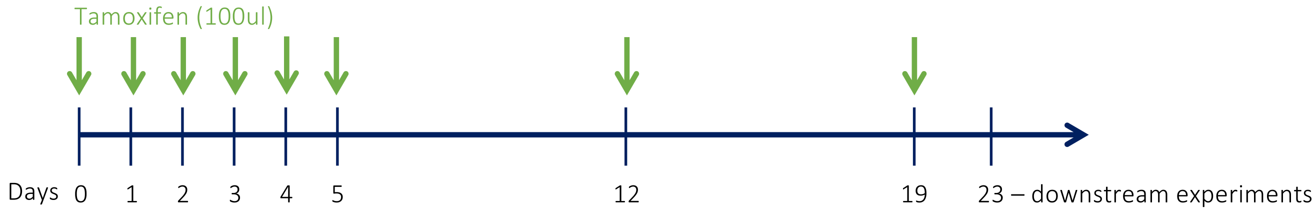

Supplementary Figure 5. Diagram of the treatment plan for tamoxifen-inducible Cre knockout mice

Fig. 6

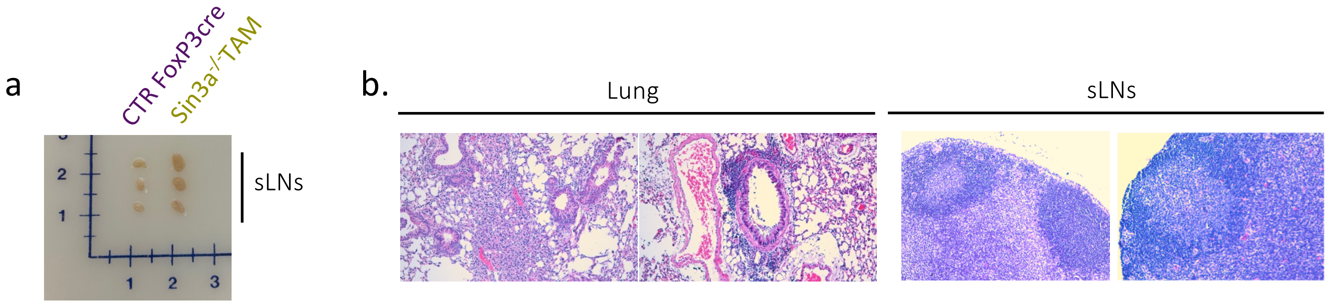

Supplementary Figure 6. Subcutaneous lymph nodes from Sin3a-/-FoxP3cre or FoxP3cre controls treated with tamoxifen as described in Figure S8 (a). H&E-stained histopathology slides (100x) from the lungs and sLNs of TAM-Sin3a$^{-/-}$FoxP3^{Cre} mice (b).

Fig. 7

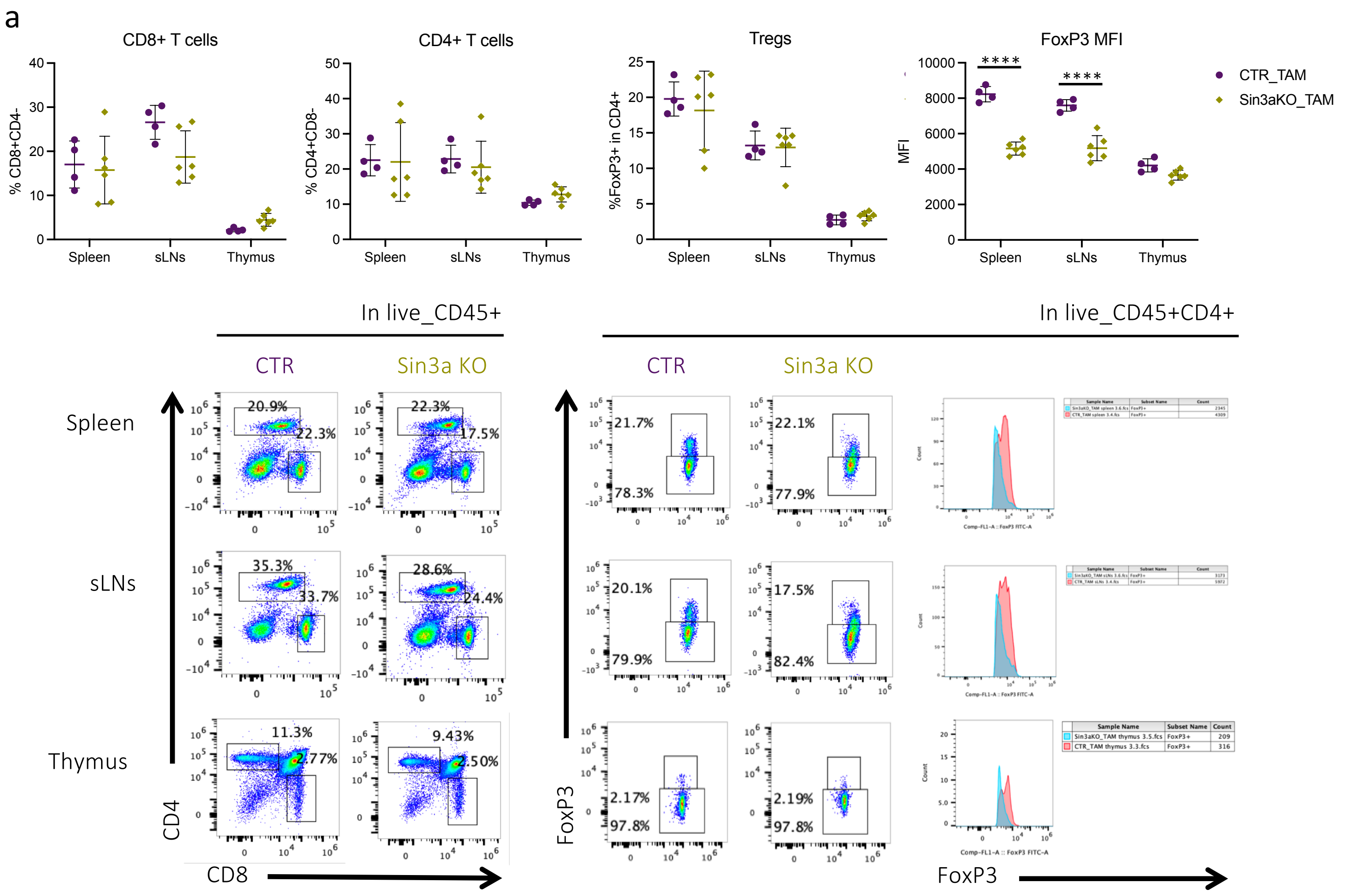

a
CD8+ T cells
CD4+ T cells
Tregs
FoxP3 MFI
% CD8+CD4-
% CD4+CD8-
%FoxP3+ in CD4+
MFI
Spleen
sLNs
Thymus
CTR_TAM
Sin3aKO_TAM
****
In live_CD45+
In live_CD45+CD4+
CTR
Sin3a KO
CD4
CD8
FoxP3
20.9%
22.3%
22.3%
17.5%
35.3%
33.7%
28.6%
24.4%
11.3%
2.77%
9.43%
2.50%
21.7%
78.3%
22.1%
77.9%
20.1%
79.9%
17.5%
82.4%
2.17%
97.8%
2.19%
97.8%

Fig. 7

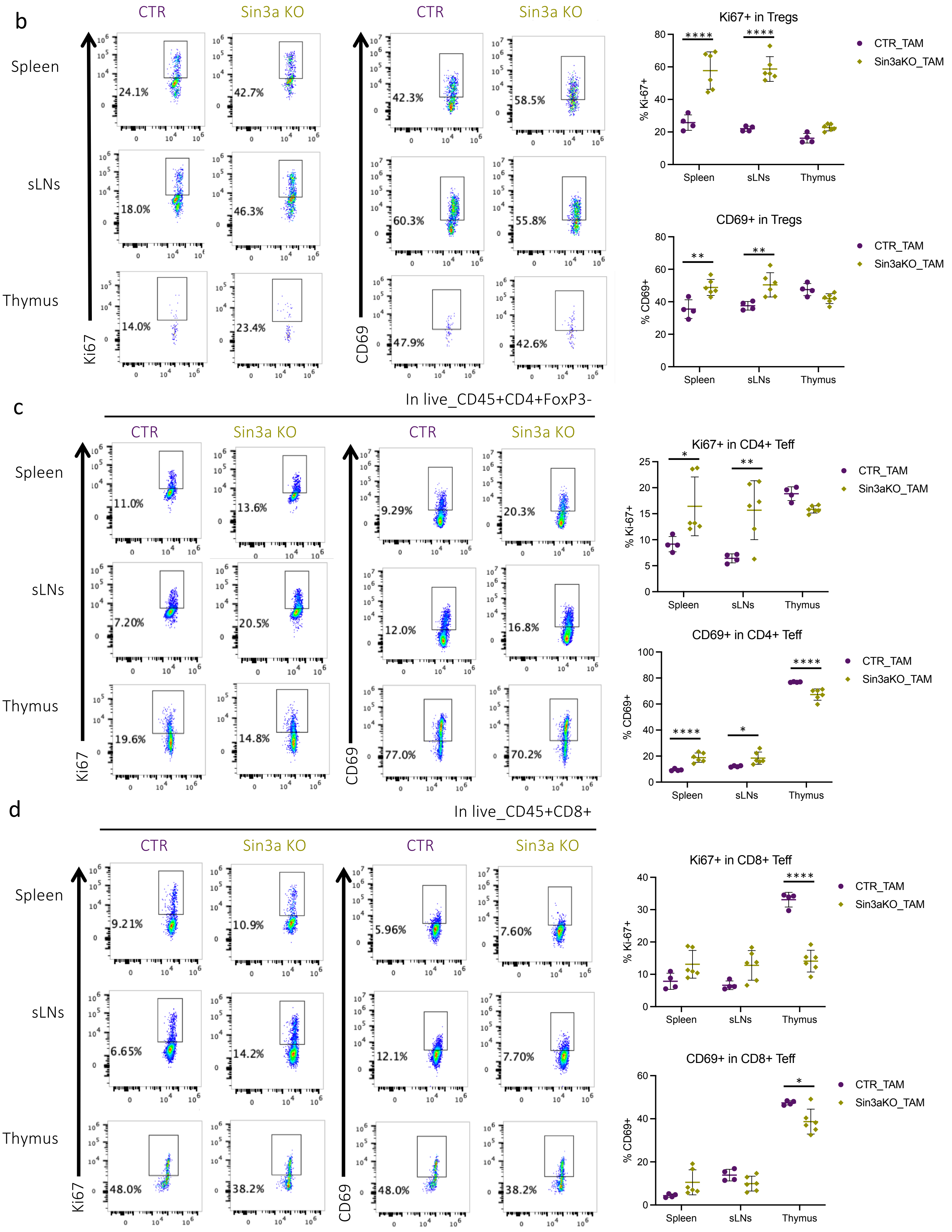
b
In live_CD45+CD4+FoxP3+
CTR
Sin3a KO
Spleen
sLNs
Thymus
Ki67
CD69
24.1%
42.7%
42.3%
58.5%
18.0%
46.3%
60.3%
55.8%
14.0%
23.4%
47.9%
42.6%
Ki67+ in Tregs
% Ki-67+
CD69+ in Tregs
% CD69+
CTR_TAM
Sin3aKO_TAM
c
In live_CD45+CD4+FoxP3-
11.0%
13.6%
9.29%
20.3%
7.20%
20.5%
12.0%
16.8%
19.6%
14.8%
77.0%
70.2%
Ki67+ in CD4+ Teff
CD69+ in CD4+ Teff
d
In live_CD45+CD8+
9.21%
10.9%
5.96%
7.60%
6.65%
14.2%
12.1%
7.70%
48.0%
38.2%
48.0%
38.2%
Ki67+ in CD8+ Teff
CD69+ in CD8+ Teff

Fig. 7

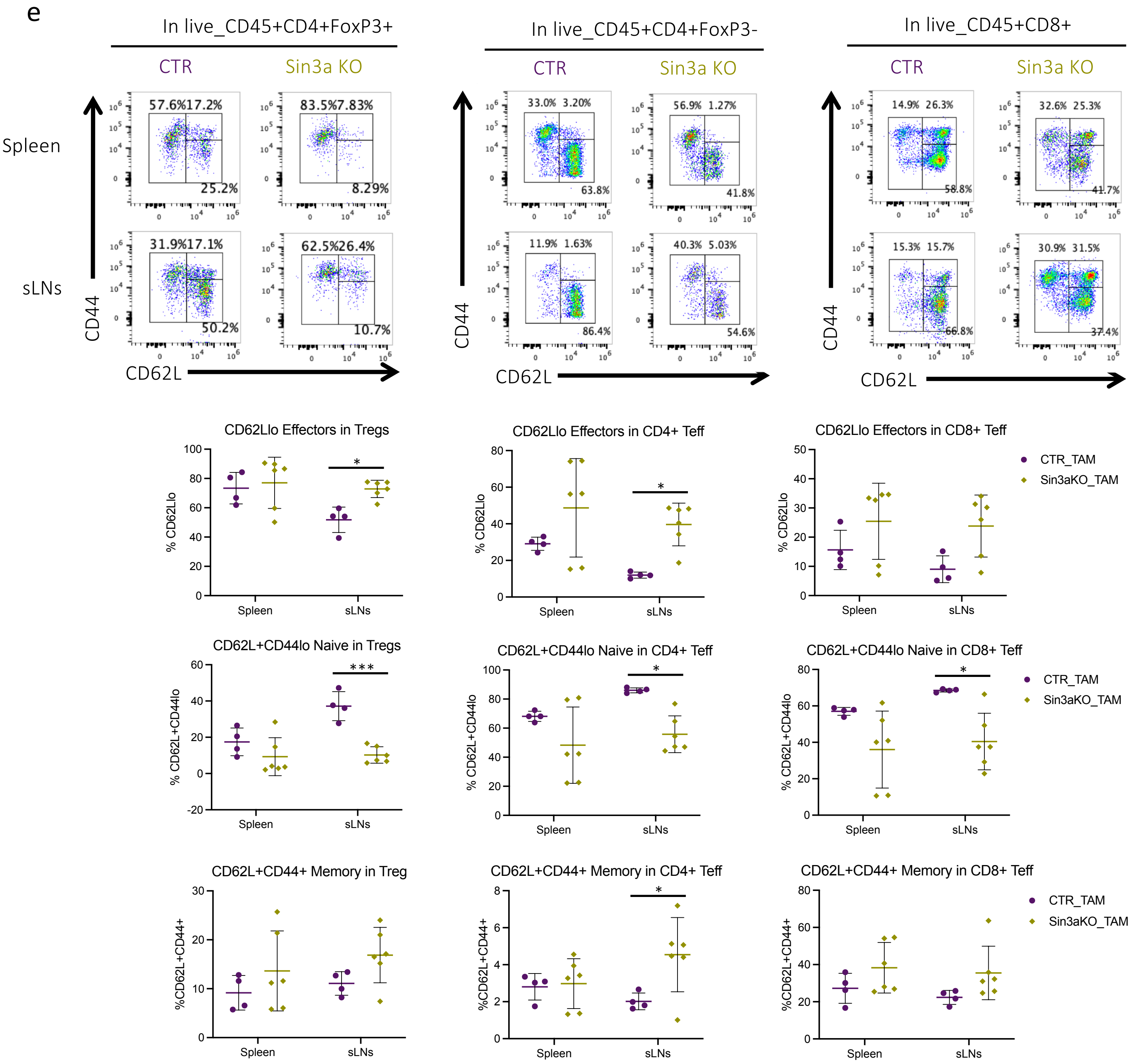

Supplementary Figure 7. The spleens, sLNs, and thymi of Sin3a$^{-/-}$FoxP3^{TAM_Cre} mice compared to Foxp3^{cre} controls following tamoxifen treatment by flow cytometry. The percent of CD8+ T effector cells (live_CD45+CD8+), CD4+ T effector cells (live_CD45+CD4+FoxP3-), and Tregs (live_CD45+CD4+FoxP3+) and the median florescent intensity of FoxP3 with in Tregs are included in graphs (top) and representative .fcs plots within live_CD45+ (left) and live_CD45+CD4+ (right) populations in the center and left columns (a). Percentages of CD69+ and Ki67+ Tregs (b), CD4+ Teffs (c), and CD8+ Teffs (d) are graphed on the right and representative .fcs plots included on the left (% Ki67) and the center (% CD69). The effector (CD62Llow), naïve (CD62L++), and memory (CD62L++CD44+) subpopulations of Treg (left), CD4+ Teff (center), and CD8+ Teff (right) cell populations are graphed (below) and rep. images included (above) (e). The number of viable cells following tissue dissociation and single cell suspension, determined via hemocytometer (f), were then used together with population percentages (depicted in S2a) to determine the actual cell numbers of Tregs, CD4+ Teff, and CD8+ Teffs (g). This experiment was performed with 6 Sin3a$^{-/-}$FoxP3^{TAM_Cre} and 4 control biological replicates (male).

Fig. 8

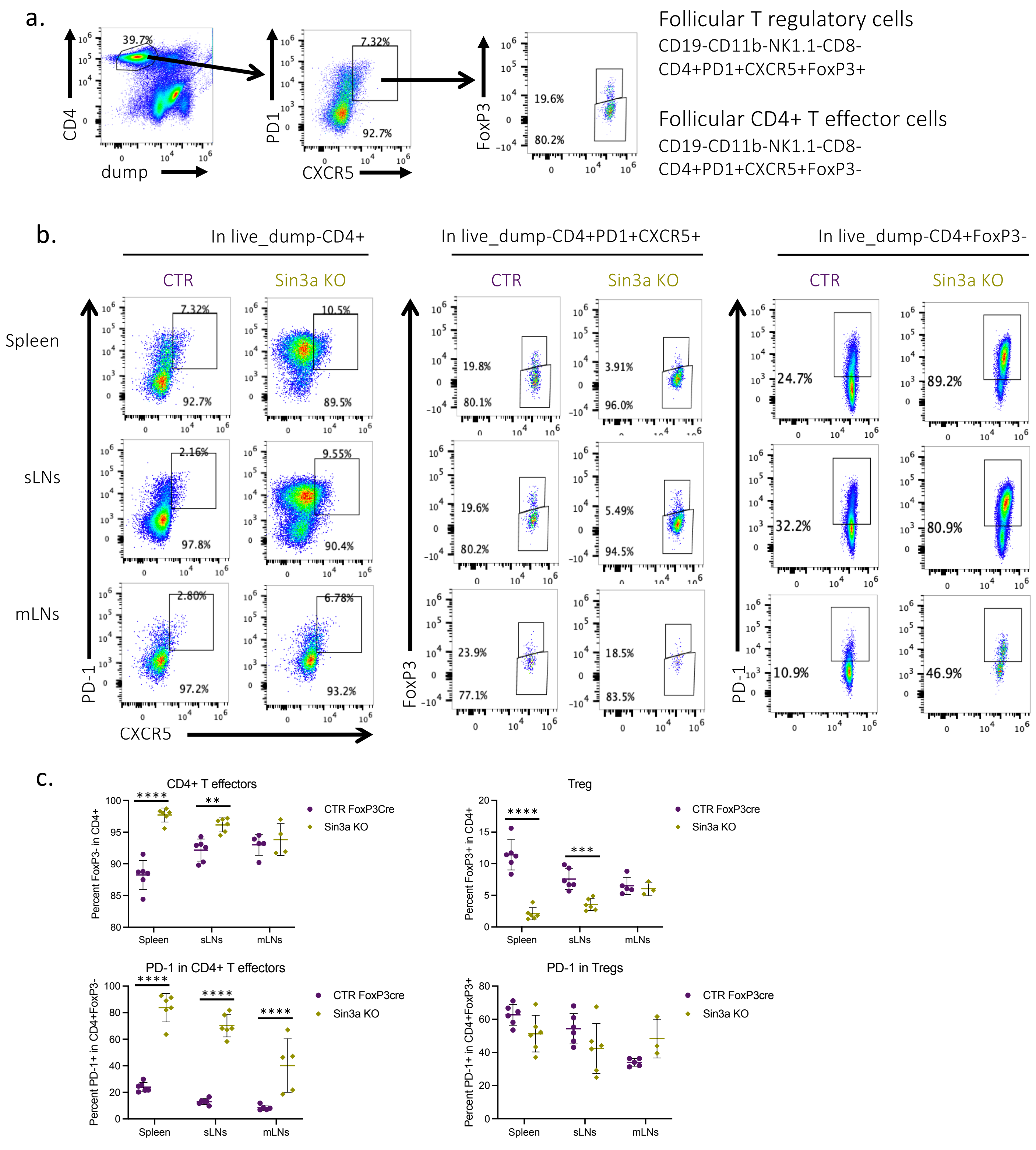

Supplementary Figure 8. The spleens, sLNs, and mLNs of Sin3a$^{-/-}$FoxP3^{Cre} mice were compared to FoxP3^{cre} controls by flow cytometry. The gating strategy used to identify follicular T cell subsets (a). All samples were first gated for fcs_ssc, singlets, and viable cells, then gated against a dump channel made up of CD19, CD8, NK1.1, and CD11b. Representative .fcs plots for PD1+CXCR5+ follicular cells (left), FoxP3+ Tfregs (center), and PD1+ in CD4+FoxP3- Teff cells (right) (b) from Sin3a-/-FoxP3cre mice and FoxP3cre controls. The percentage of PD1+ cells within CD4+ Teff (left) and Tregs (right). For this experiment 6 (3 male and 3 female) biological replicates per group were used.

Fig. 9

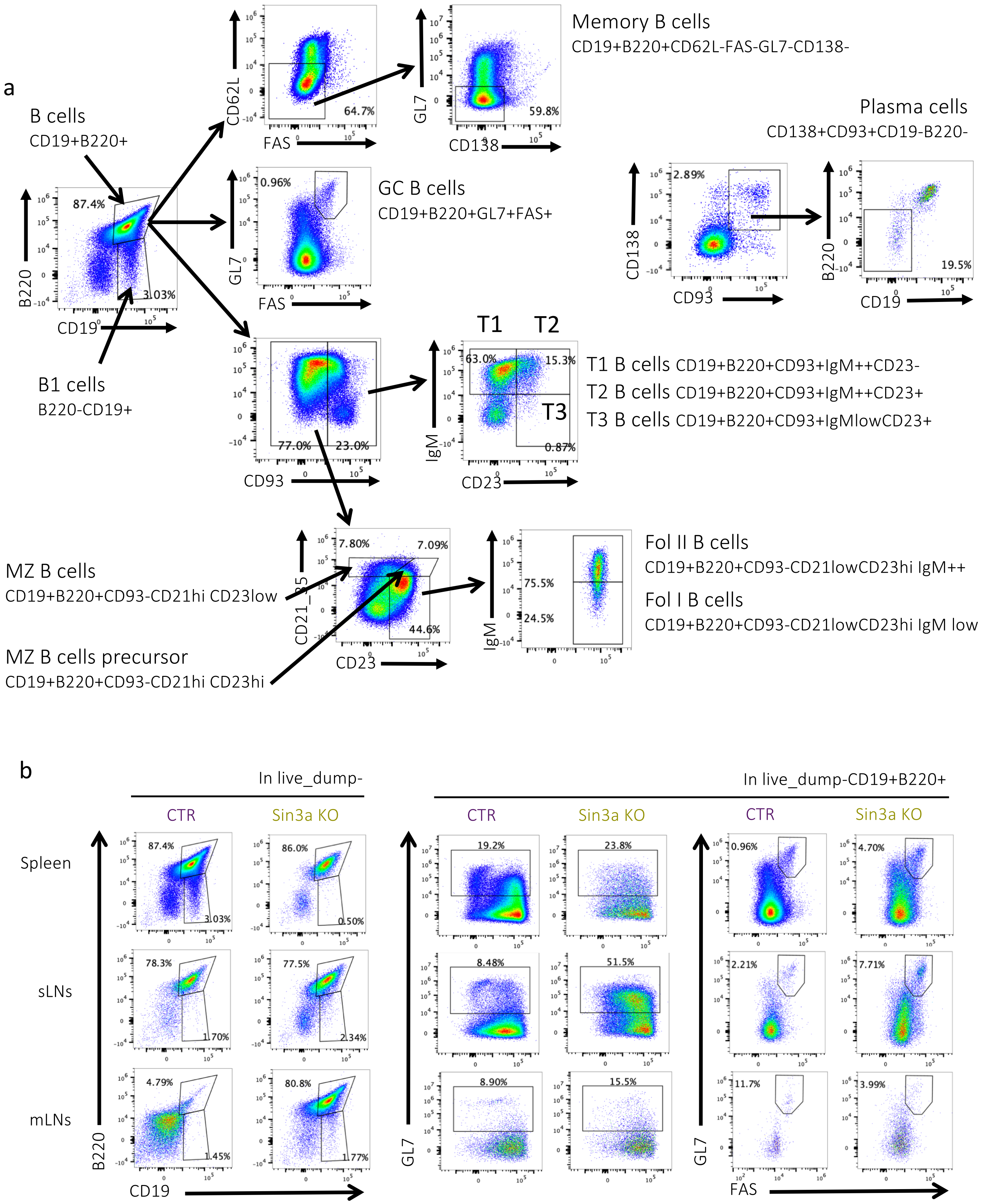
a
B cells
CD19+B220+
B1 cells
B220-CD19+
Memory B cells
CD19+B220+CD62L-FAS-GL7-CD138-
GC B cells
CD19+B220+GL7+FAS+
Plasma cells
CD138+CD93+CD19-B220-
T1 B cells CD19+B220+CD93+IgM++CD23-
T2 B cells CD19+B220+CD93+IgM++CD23+
T3 B cells CD19+B220+CD93+IgMlowCD23+
MZ B cells
CD19+B220+CD93-CD21hi CD23low
MZ B cells precursor
CD19+B220+CD93-CD21hi CD23hi
Fol II B cells
CD19+B220+CD93-CD21lowCD23hi IgM++
Fol I B cells
CD19+B220+CD93-CD21lowCD23hi IgM low
b
In live_dump-
In live_dump-CD19+B220+
CTR
Sin3a KO
Spleen
sLNs
mLNs
B220
CD19
GL7
FAS

Fig. 9

c

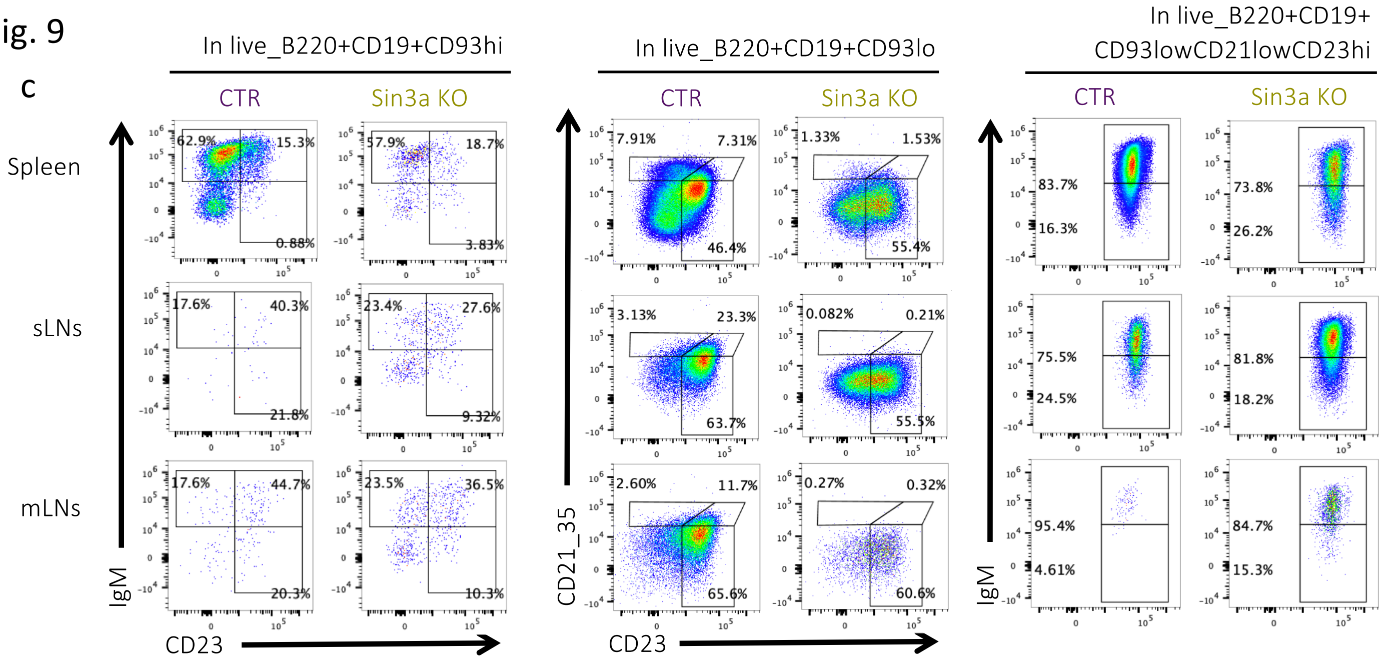
In live_B220+CD19+CD93hi
In live_B220+CD19+CD93lo
In live_B220+CD19+ CD93lowCD21lowCD23hi
CTR
Sin3a KO
Spleen
sLNs
mLNs
IgM
CD23
CD21_35
62.9%
15.3%
0.88%
57.9%
18.7%
3.83%
17.6%
40.3%
21.8%
23.4%
27.6%
9.32%
17.6%
44.7%
20.3%
23.5%
36.5%
10.3%
7.91%
7.31%
46.4%
1.33%
1.53%
55.4%
3.13%
23.3%
63.7%
0.082%
0.21%
55.5%
2.60%
11.7%
65.6%
0.27%
0.32%
60.6%
83.7%
16.3%
73.8%
26.2%
75.5%
24.5%
81.8%
18.2%
95.4%
4.61%
84.7%
15.3%

d

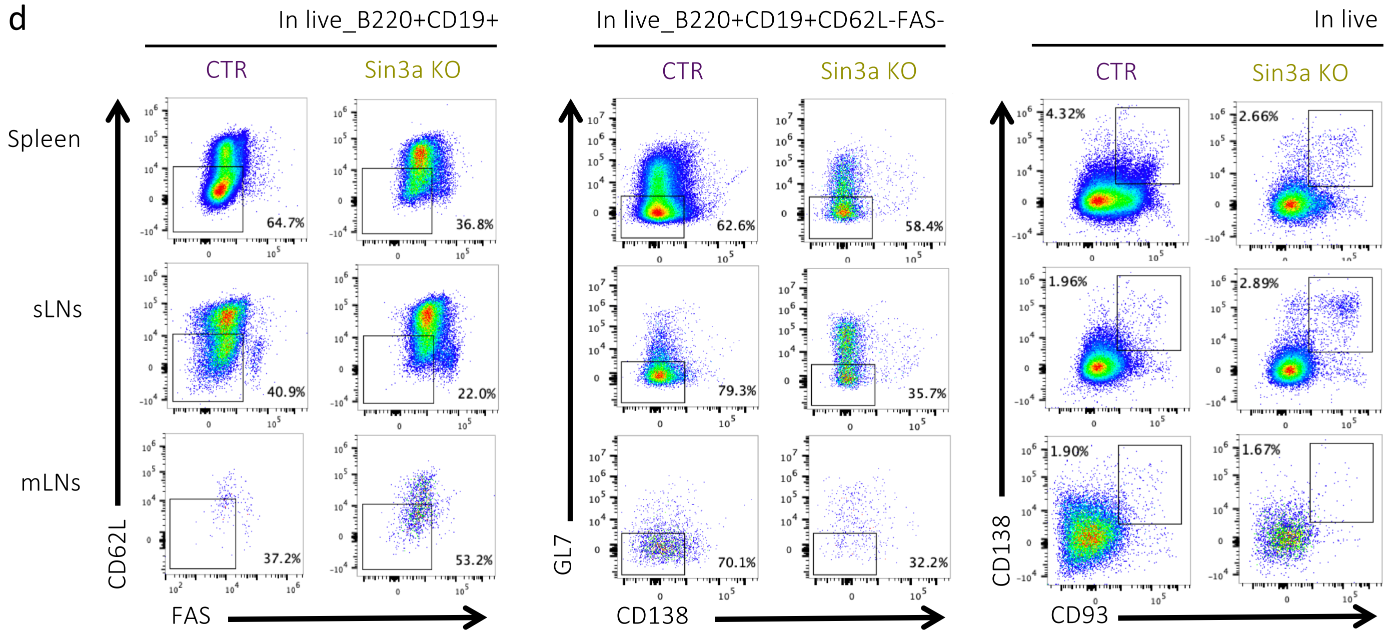
In live_B220+CD19+
In live_B220+CD19+CD62L-FAS-
In live
CTR
Sin3a KO
Spleen
sLNs
mLNs
CD62L
FAS
GL7
CD138
CD93
64.7%
36.8%
40.9%
22.0%
37.2%
53.2%
62.6%
58.4%
79.3%
35.7%
70.1%
32.2%
4.32%
2.66%
1.96%
2.89%
1.90%
1.67%

e

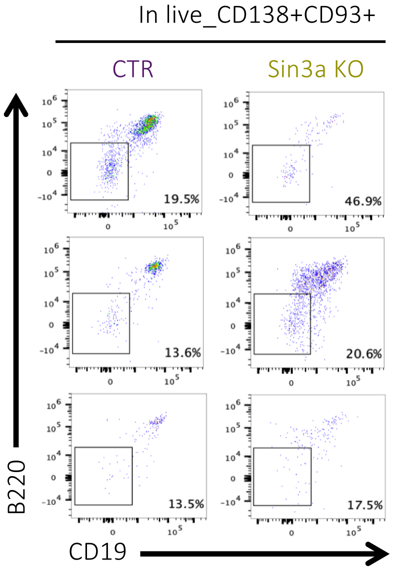
In live_CD138+CD93+
CTR
Sin3a KO
B220
CD19
19.5%
46.9%
13.6%
20.6%
13.5%
17.5%

Fig. 9

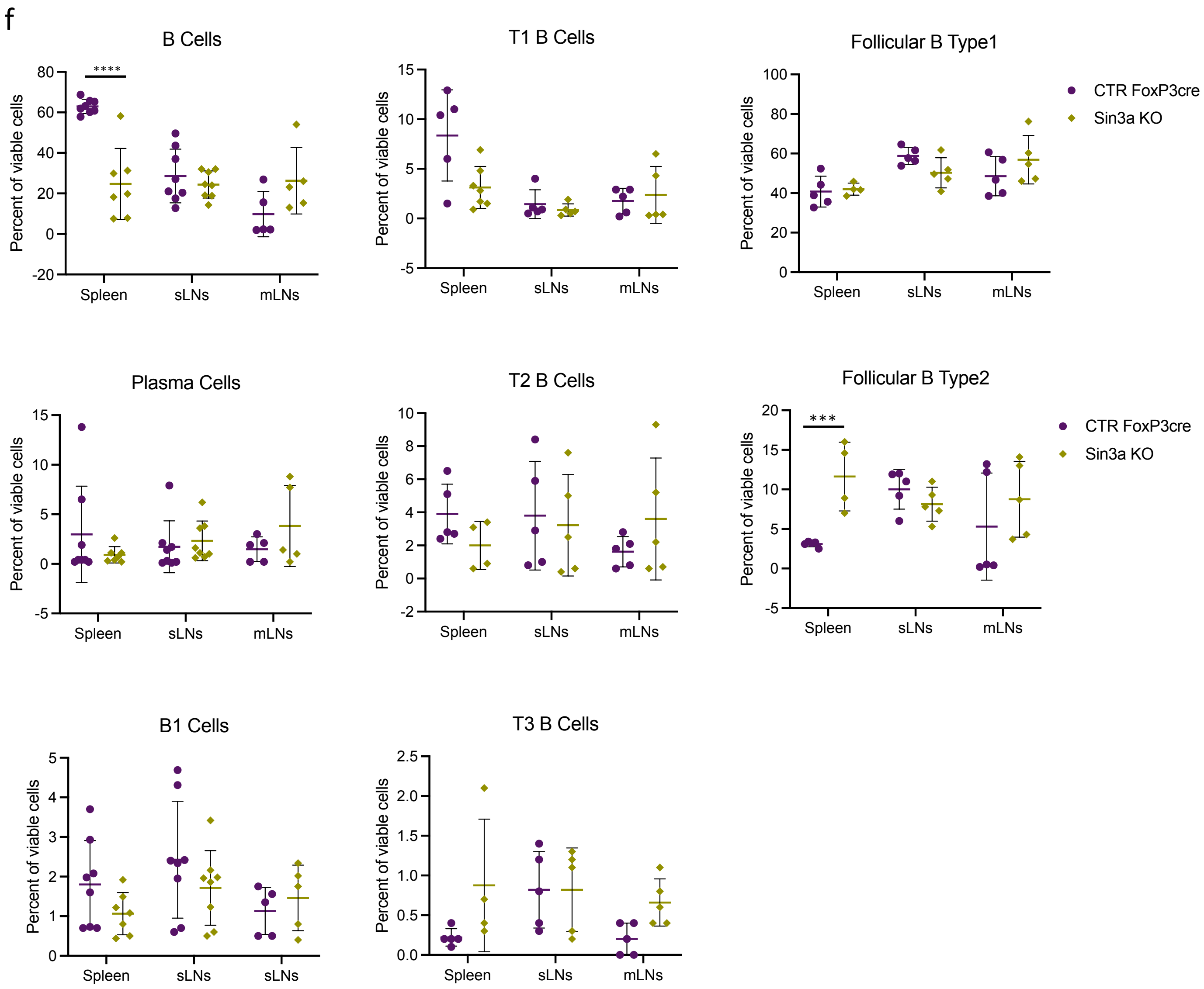

Supplementary Figure 9. The spleens, sLNs, and mLNs of Sin3a$^{-/-}$FoxP3^{Cre} mice were compared to FoxP3^{cre} controls by flow cytometry. B cell populations were determined according to the illustrated gating scheme (a). All samples were first gated for fsc ssc, singlets, and viable events and against a dump channel including NK1.1, CD8, CD4, and CD11b. Representative .fcs plots for B (CD19+B220+) and B1 (CD19+B220-) cells (b, left), GL7 activated within B cells (b, center), and Germinal Center B (CD19+B220+GL7+FAS+) cells (b, right). Representative .fcs images for IgM/CD23 within CD93++ B cells (c, left), CD21_35/CD23 within CD93low B cells (c, center), and IgM within follicular B cells (c, right). Representative .fcs plots of CD62L/FAS within B cells (d, left), GL7/CD138 within CD62L-FAS- B cells (d, center), and CD138/CD93 within live cells (d, right). Images of representative .fcs plots of CD19/B220 within CD138+CD93+ cells (e). B cell population percentages from individual markers were used to back calculate the population percentage within viable lymphocytes (f). and For this experiment 8 (4 males and 4 females) biological replicates per group were used.

Fig. 10

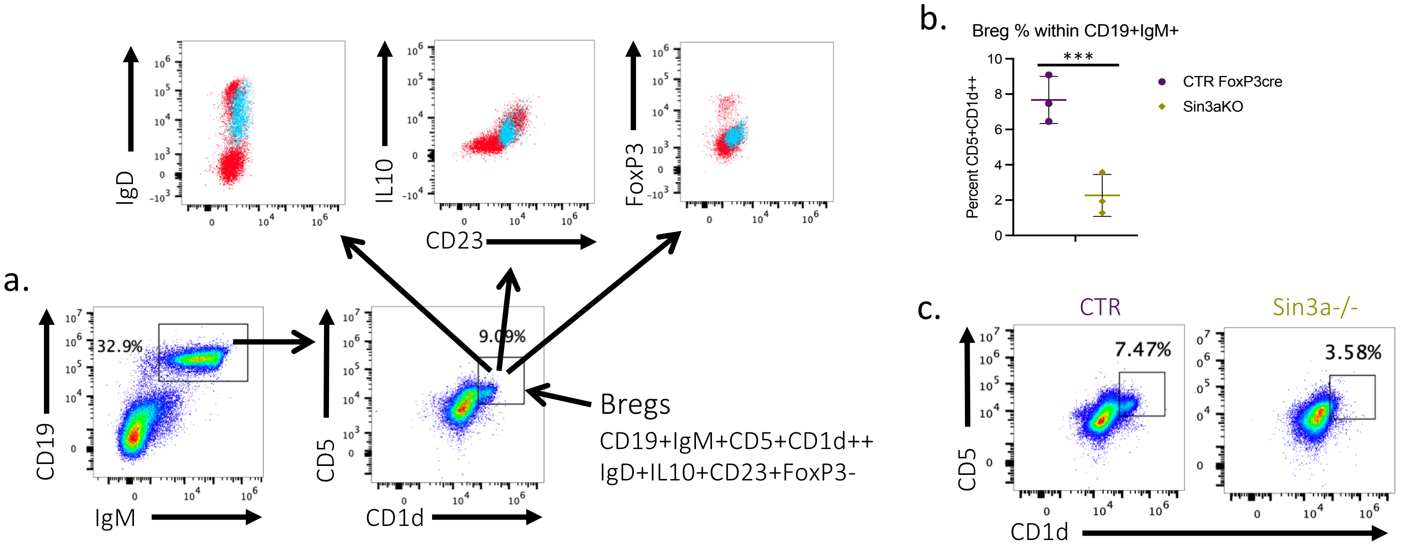

Supplementary Figure 10. Subcutaneous lymph nodes of Sin3a$^{-/-}$FoxP3^{Cre} mice were compared to FoxP3^{cre} controls by flow cytometry. The gating strategy used to identify B regulatory cells (a). All samples were first gated for fcs_ssc, singlets, and viable cells. Representative .fcs plots for PD1+CXCR5+ follicular cells (left), FoxP3+ Tfregs (center), and PD1+ in CD4+FoxP3- Teff cells (right) (b) from Sin3a-/-FoxP3cre mice and FoxP3cre controls. The percentage of PD1+ cells within CD4+ Teff (left) and Tregs (right). This experiment used 3 male biological replicates per group.

Fig. 11

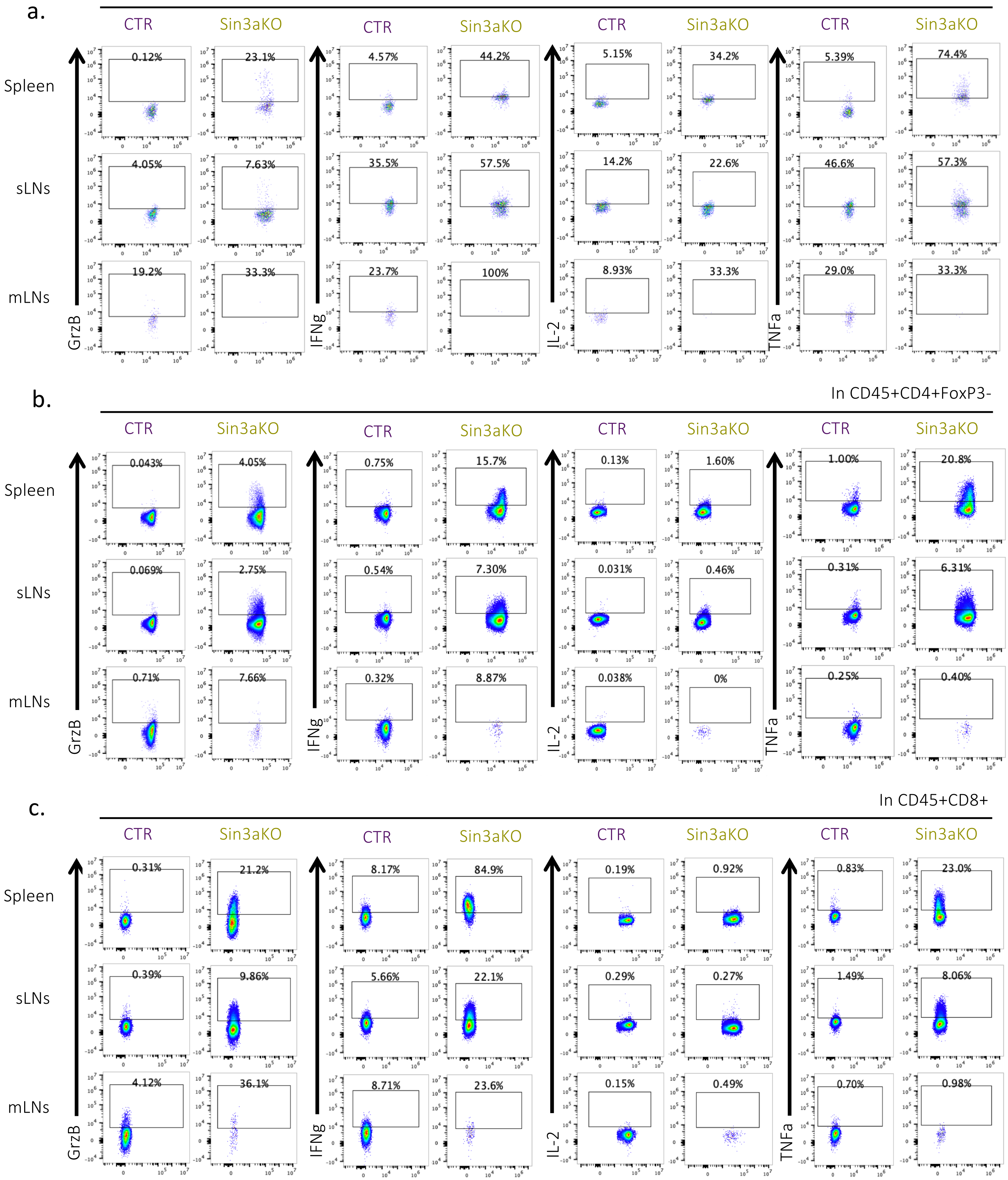

Supplementary Figure 11. Cells from spleens, sLNs, or mLNs of Sin3a$^{-/-}$FoxP3^{Cre} mice or controls were stimulated with for 48 hours, treated with PMA/Iono/Bref for 3 hours, then evaluated for cytokine production by flow cytometry. Representative .fcs plots for Granzyme B, IFNg, IL-2, and TNFa (from left to right), in Tregs (a), CD4+ Teffs (b), and CD8+ Teffs (c).

Fig. 12

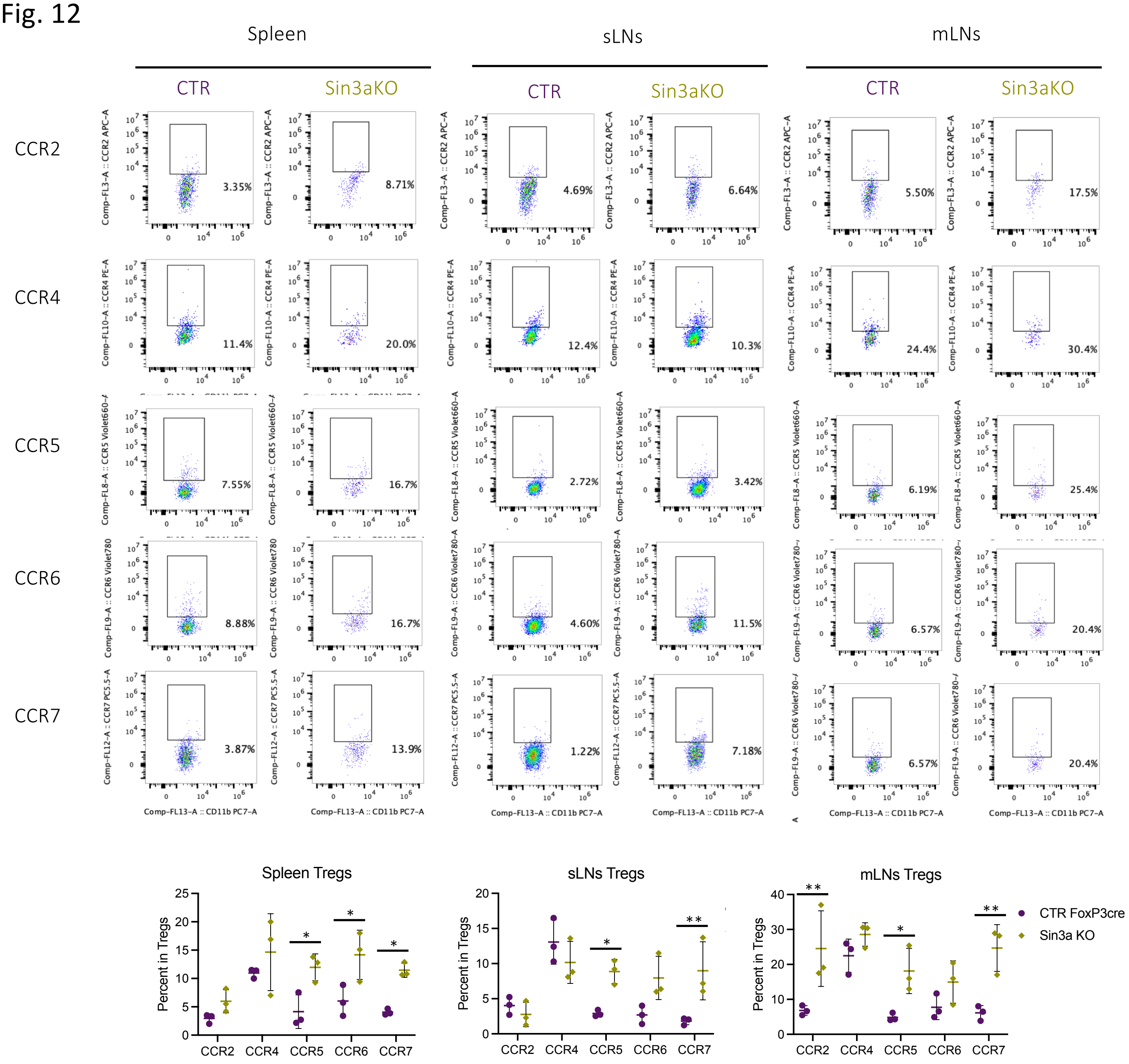

Supplementary Figure 12. Cells from spleens, sLNs, and mLNs of Sin3a$^{-/-}$FoxP3^{Cre} mice or FoxP3cre controls were stained for various CCRs and evaluated by flow cytometry. Representative .fcs plots for CCRs within CD4+FoxP3+ Tregs in Spleen (left), sLNs (center), and sMLs (right), with grouped data graphed below. This experiment was conducted with 3 (male) biological replicates per group.

Fig. 13

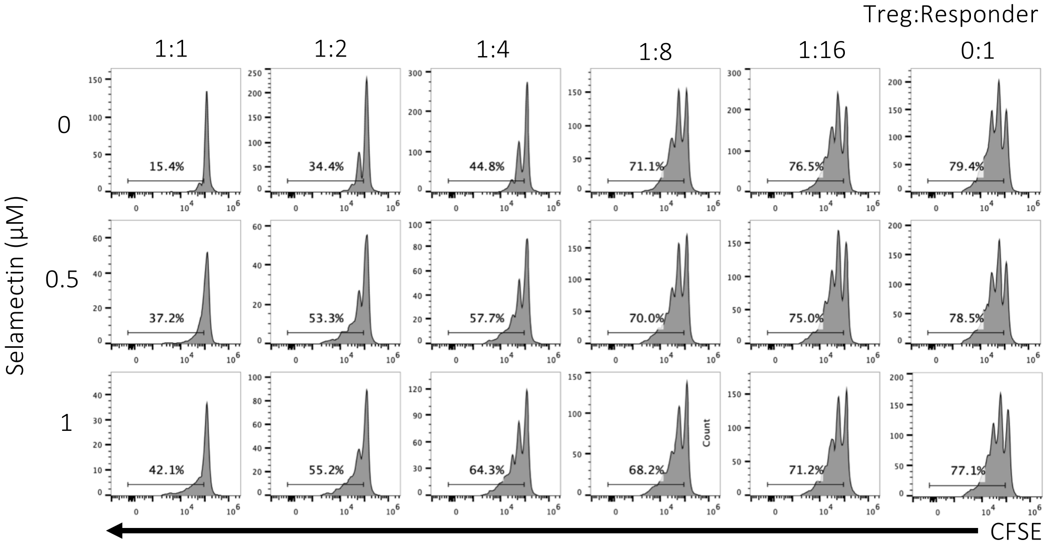

Supplementary Figure 13. Treg suppression assay performed with the Sin3-inhibitor, selemectin. WT CD4+CD25+ cells were serialy diluted together with CFSE-labeled splenic responder cells and incubated for 3 days with or without selemectin treatment, representative CFSE division histograms are included. This experiment was performed with 2 biological replicates and technical triplicates.

Fig. 14

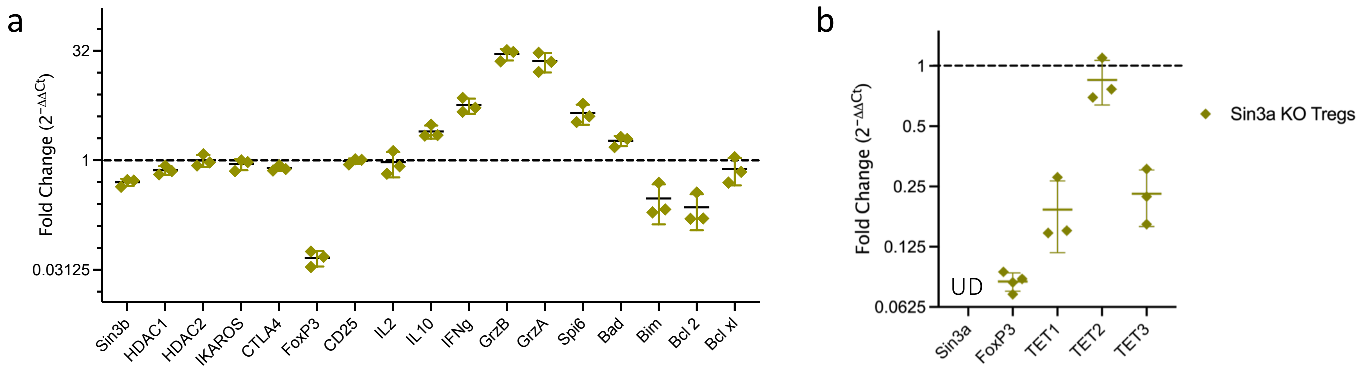

Supplementary Figure 14. RNA expression detected by RT-qPCR of CD4+CD25+ Tregs isolated by magnetic beads (a), or CD25+FoxP3+ Tregs isolated by FACS sorting (b) from Sin3a-/-FoxP3cre mice. Data were normalized to endogenous 18S and control target expression. Undetected Ct values are indicated by 'UD'.

Fig. 15

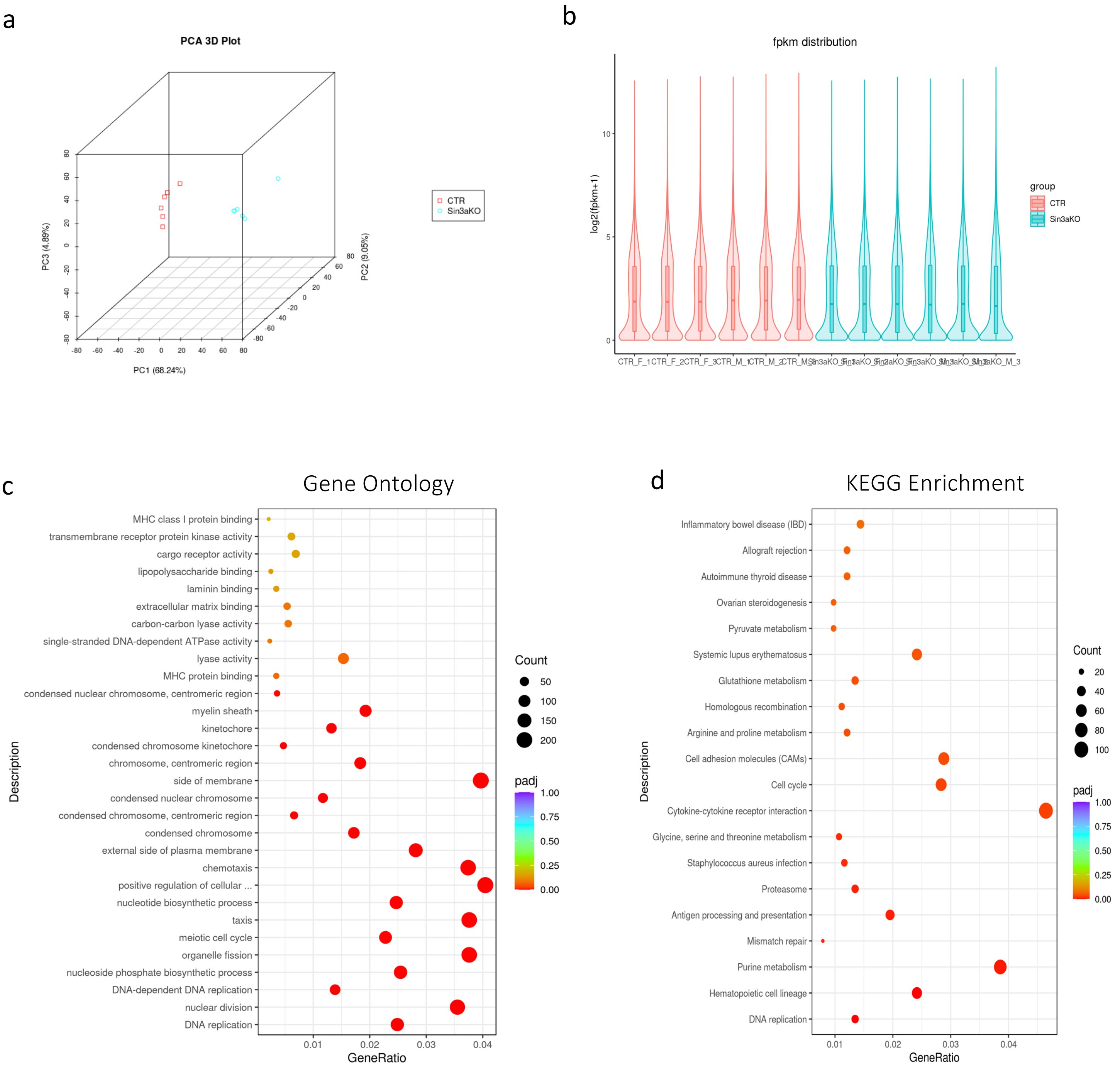
a
PCA 3D Plot
PC1 (68.24%)
PC2 (9.05%)
PC3 (4.89%)
CTR
Sin3aKO
b
fpkm distribution
log2(fpkm+1)
group
CTR
Sin3aKO
c
Gene Ontology
MHC class I protein binding
transmembrane receptor protein kinase activity
cargo receptor activity
lipopolysaccharide binding
laminin binding
extracellular matrix binding
carbon-carbon lyase activity
single-stranded DNA-dependent ATPase activity
lyase activity
MHC protein binding
condensed nuclear chromosome, centromeric region
myelin sheath
kinetochore
condensed chromosome kinetochore
chromosome, centromeric region
side of membrane
condensed nuclear chromosome
condensed chromosome, centromeric region
condensed chromosome
external side of plasma membrane
chemotaxis
positive regulation of cellular ...
nucleotide biosynthetic process
taxis
meiotic cell cycle
organelle fission
nucleoside phosphate biosynthetic process
DNA-dependent DNA replication
nuclear division
DNA replication
Description
GeneRatio
Count
padj
d
KEGG Enrichment
Inflammatory bowel disease (IBD)
Allograft rejection
Autoimmune thyroid disease
Ovarian steroidogenesis
Pyruvate metabolism
Systemic lupus erythematosus
Glutathione metabolism
Homologous recombination
Arginine and proline metabolism
Cell adhesion molecules (CAMs)
Cell cycle
Cytokine-cytokine receptor interaction
Glycine, serine and threonine metabolism
Staphylococcus aureus infection
Proteasome
Antigen processing and presentation
Mismatch repair
Purine metabolism
Hematopoietic cell lineage
DNA replication
Description
GeneRatio
Count
padj

Fig. 15

e

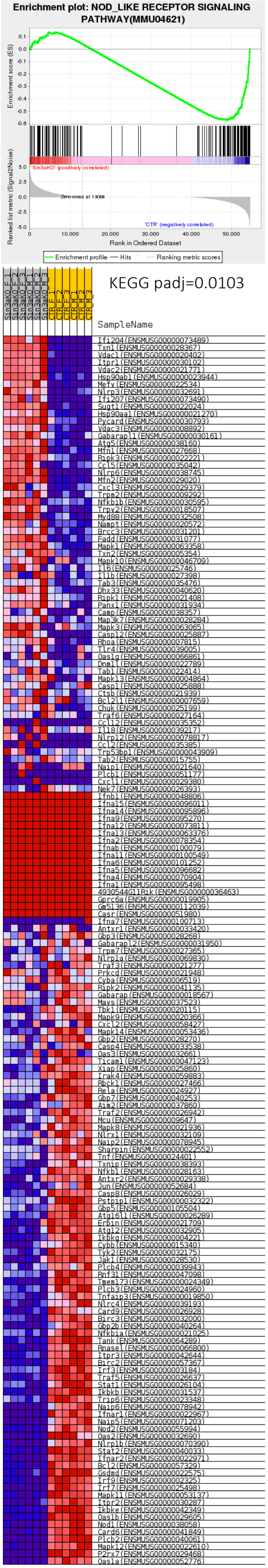

Enrichment plot: NOD_LIKE RECEPTOR SIGNALING PATHWAY(MMU04621)
KEGG padj=0.0103
SampleName

f

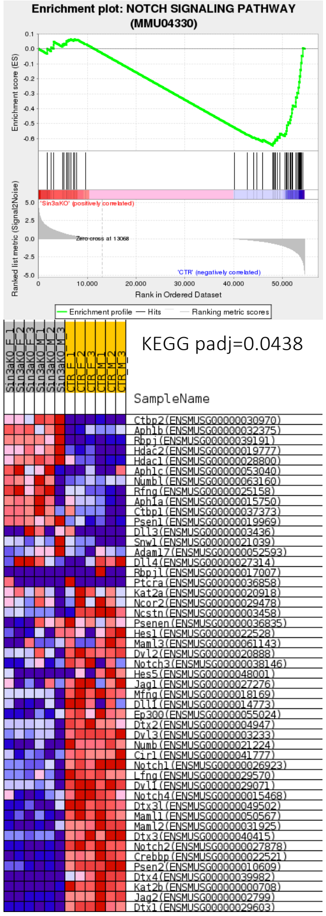

Enrichment plot: NOTCH SIGNALING PATHWAY (MMU04330)
KEGG padj=0.0438
SampleName

g

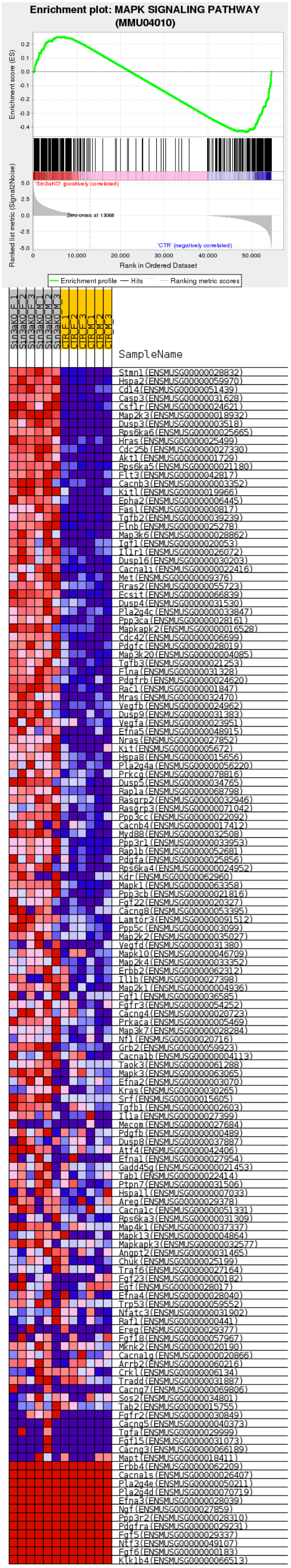

Enrichment plot: MAPK SIGNALING PATHWAY (MMU04010)
SampleName

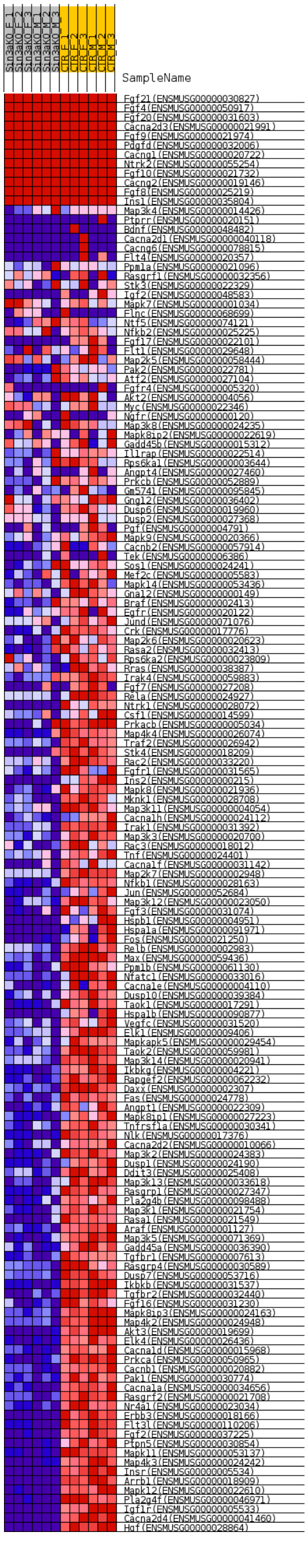

SampleName

Fig. 15

h

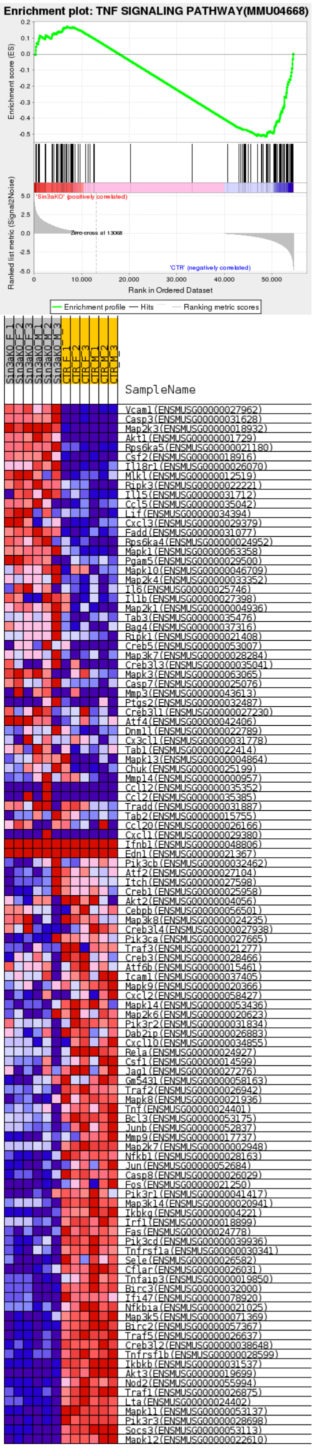
Enrichment plot: TNF SIGNALING PATHWAY(MMU04668)
Enrichment score (ES)
Ranked list metric (Signal2Noise)
'Sin3aKO' (positively correlated)
Zero cross at 13068
'CTR' (negatively correlated)
Rank in Ordered Dataset
Enrichment profile — Hits — Ranking metric scores
SampleName

i

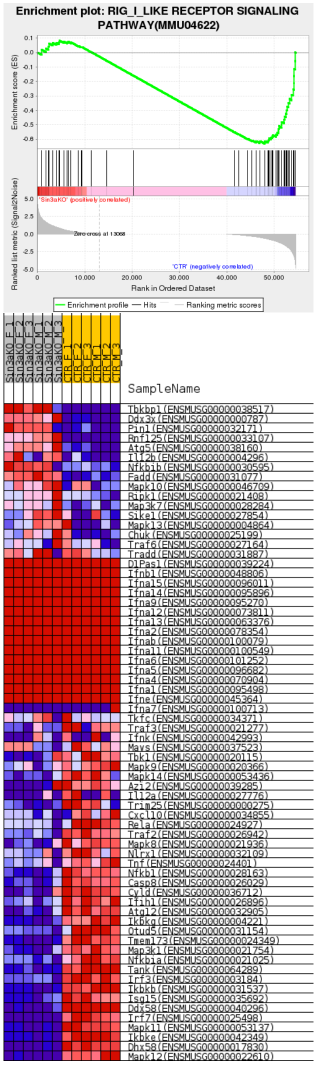
Enrichment plot: RIG_I_LIKE RECEPTOR SIGNALING PATHWAY(MMU04622)
Enrichment score (ES)
Ranked list metric (Signal2Noise)
'Sin3aKO' (positively correlated)
Zero cross at 13068
'CTR' (negatively correlated)
Rank in Ordered Dataset
Enrichment profile — Hits — Ranking metric scores
SampleName

j

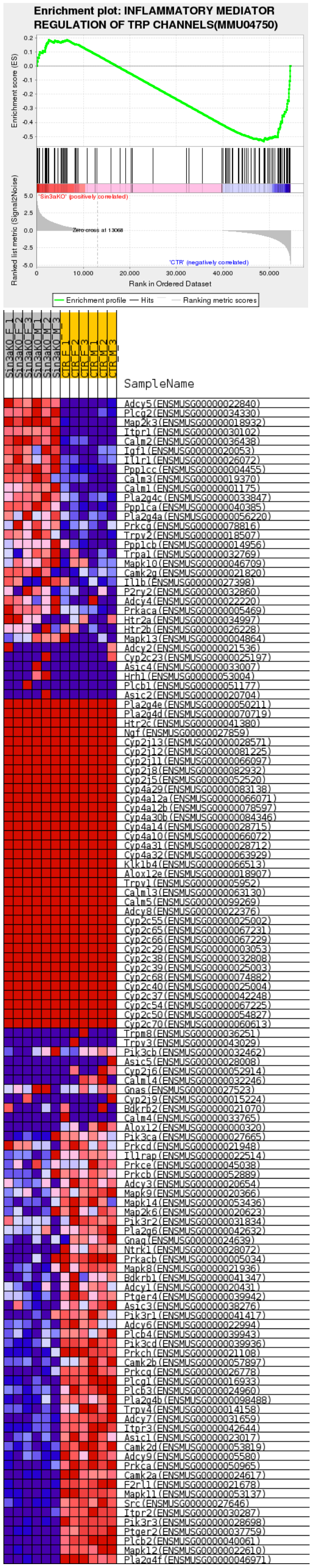
Enrichment plot: INFLAMMATORY MEDIATOR REGULATION OF TRP CHANNELS(MMU04750)
Enrichment score (ES)
Ranked list metric (Signal2Noise)
'Sin3aKO' (positively correlated)
Zero cross at 13068
'CTR' (negatively correlated)
Rank in Ordered Dataset
Enrichment profile — Hits — Ranking metric scores
SampleName

k

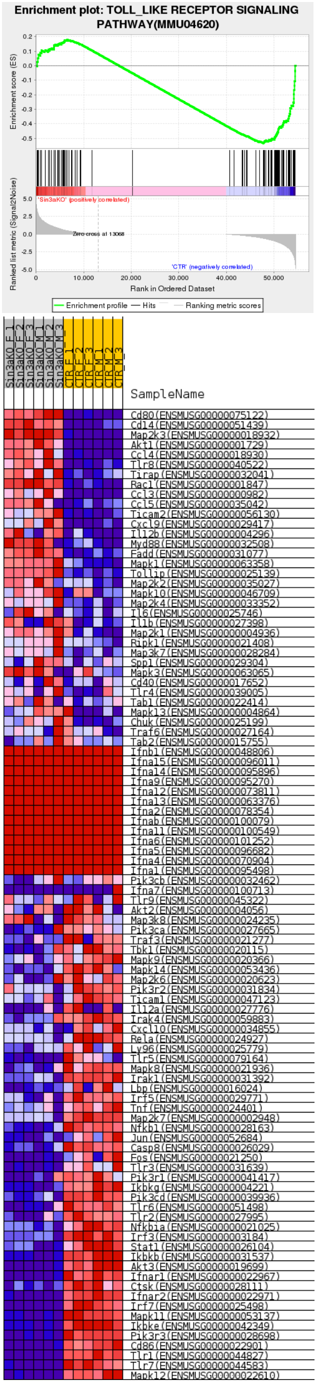
Enrichment plot: TOLL_LIKE RECEPTOR SIGNALING PATHWAY(MMU04620)
Enrichment score (ES)
Ranked list metric (Signal2Noise)
'Sin3aKO' (positively correlated)
Zero cross at 13068
'CTR' (negatively correlated)
Rank in Ordered Dataset
Enrichment profile — Hits — Ranking metric scores
SampleName

Fig. 15

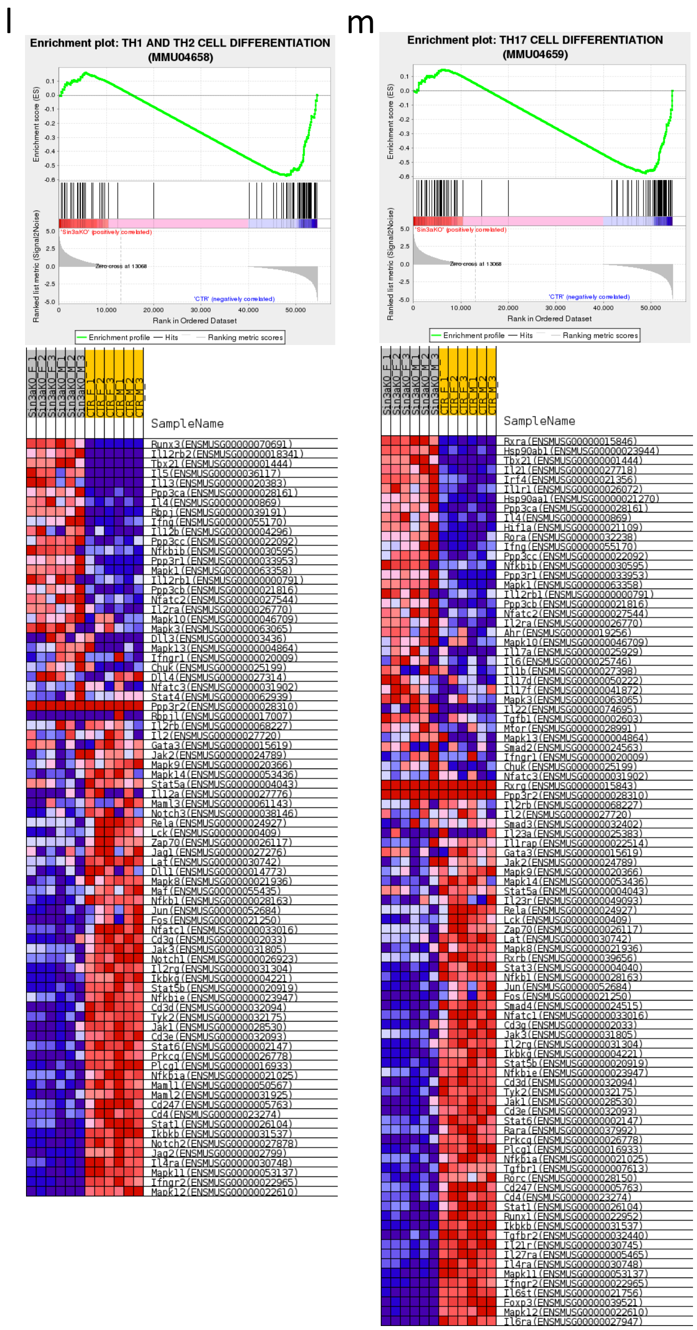

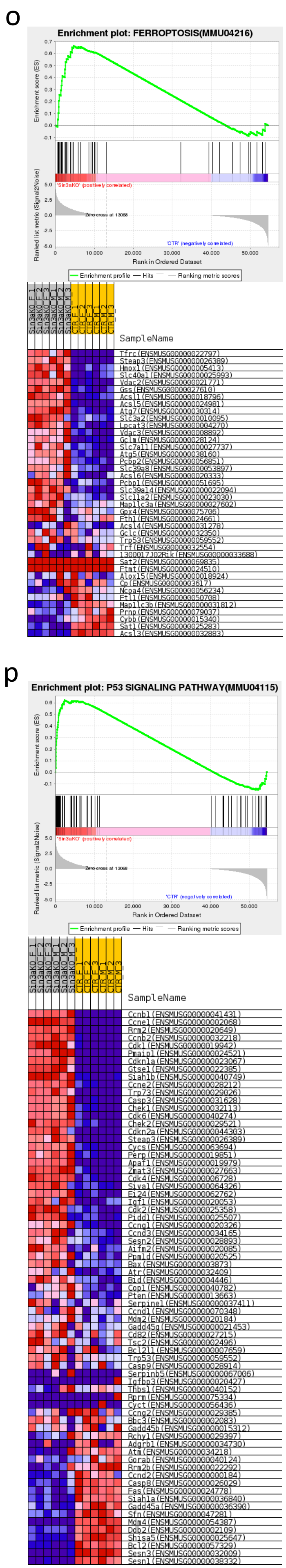

Supplementary Figure 15. RNAseq analysis of CD25+FoxP3+ Tregs from Sin3a-/- or FoxP3cre control mice. Sample similarity depicted by three-dimensional principal component analysis (a) and violin plot of fpmk distribution (b). Gene Ontology (GO) (c) and KEGG (d) enrichment analyses of differentially expressed genes (DEGs). Enrichment plots of DEGs with corresponding heat maps below showing NOD-like signaling pathway (e), NOTCH signaling pathway (f), MAPK signaling pathway (g), TNF signaling (h), RIG-I-like receptor signaling pathway (i), inflammatory mediator regulation of TRP channels (j), Toll-like receptor signaling pathway (k), TH1 and TH2 cell differentiation (l), TH17 cell differentiation (m), antigen processing and presentation (n), Ferroptosis (o), and the P53 signaling pathway (p).

Fig. 16
a.

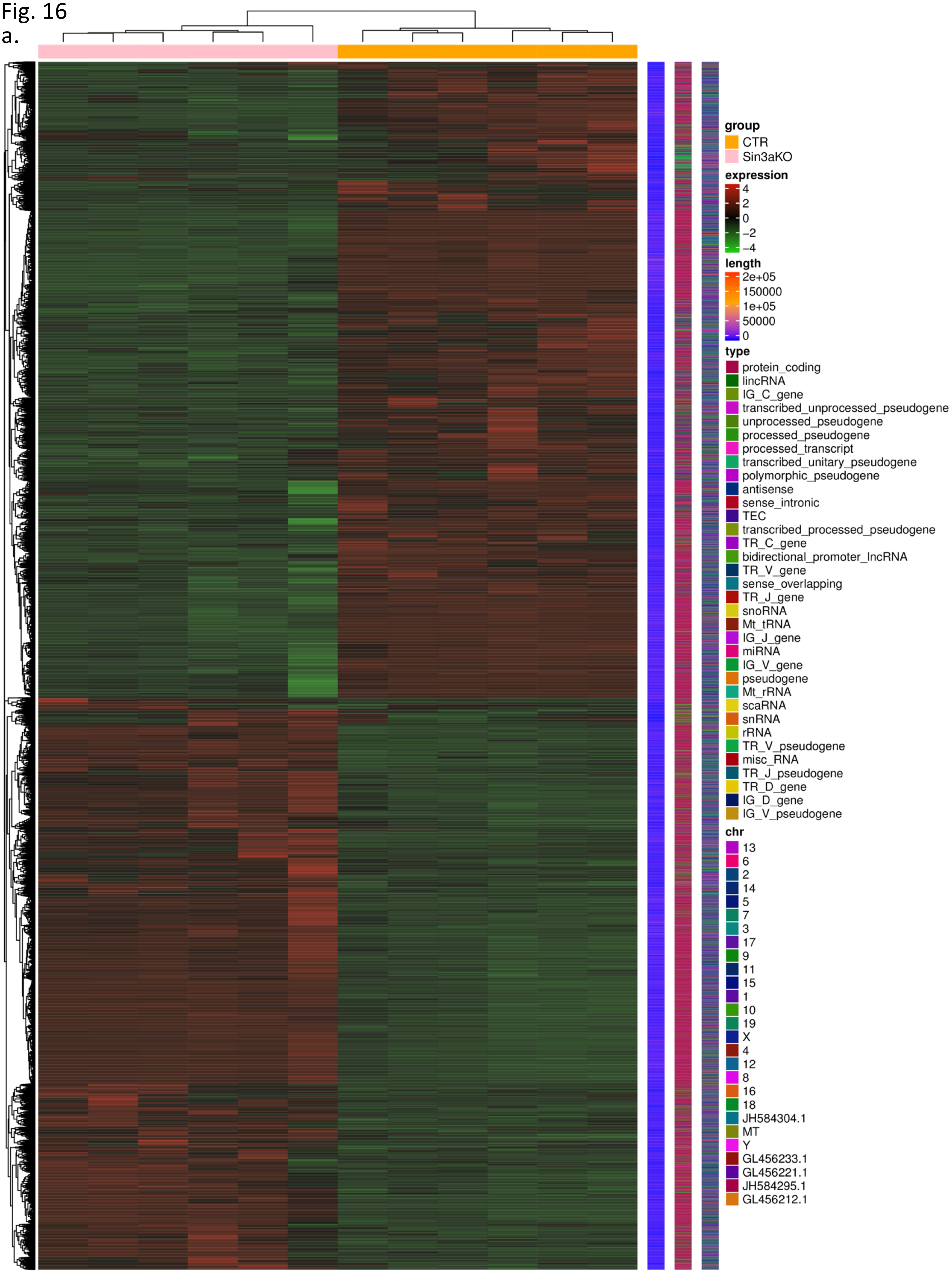
group
CTR
Sin3aKO
expression
4
2
0
−2
−4
length
2e+05
150000
1e+05
50000
0
type
protein_coding
lincRNA
IG_C_gene
transcribed_unprocessed_pseudogene
unprocessed_pseudogene
processed_pseudogene
processed_transcript
transcribed_unitary_pseudogene
polymorphic_pseudogene
antisense
sense_intronic
TEC
transcribed_processed_pseudogene
TR_C_gene
bidirectional_promoter_lncRNA
TR_V_gene
sense_overlapping
TR_J_gene
snoRNA
Mt_tRNA
IG_J_gene
miRNA
IG_V_gene
pseudogene
Mt_rRNA
scaRNA
snRNA
rRNA
TR_V_pseudogene
misc_RNA
TR_J_pseudogene
TR_D_gene
IG_D_gene
IG_V_pseudogene
chr
13
6
2
14
5
7
3
17
9
11
15
1
10
19
X
4
12
8
16
18
JH584304.1
MT
Y
GL456233.1
GL456221.1
JH584295.1
GL456212.1

Fig. 16

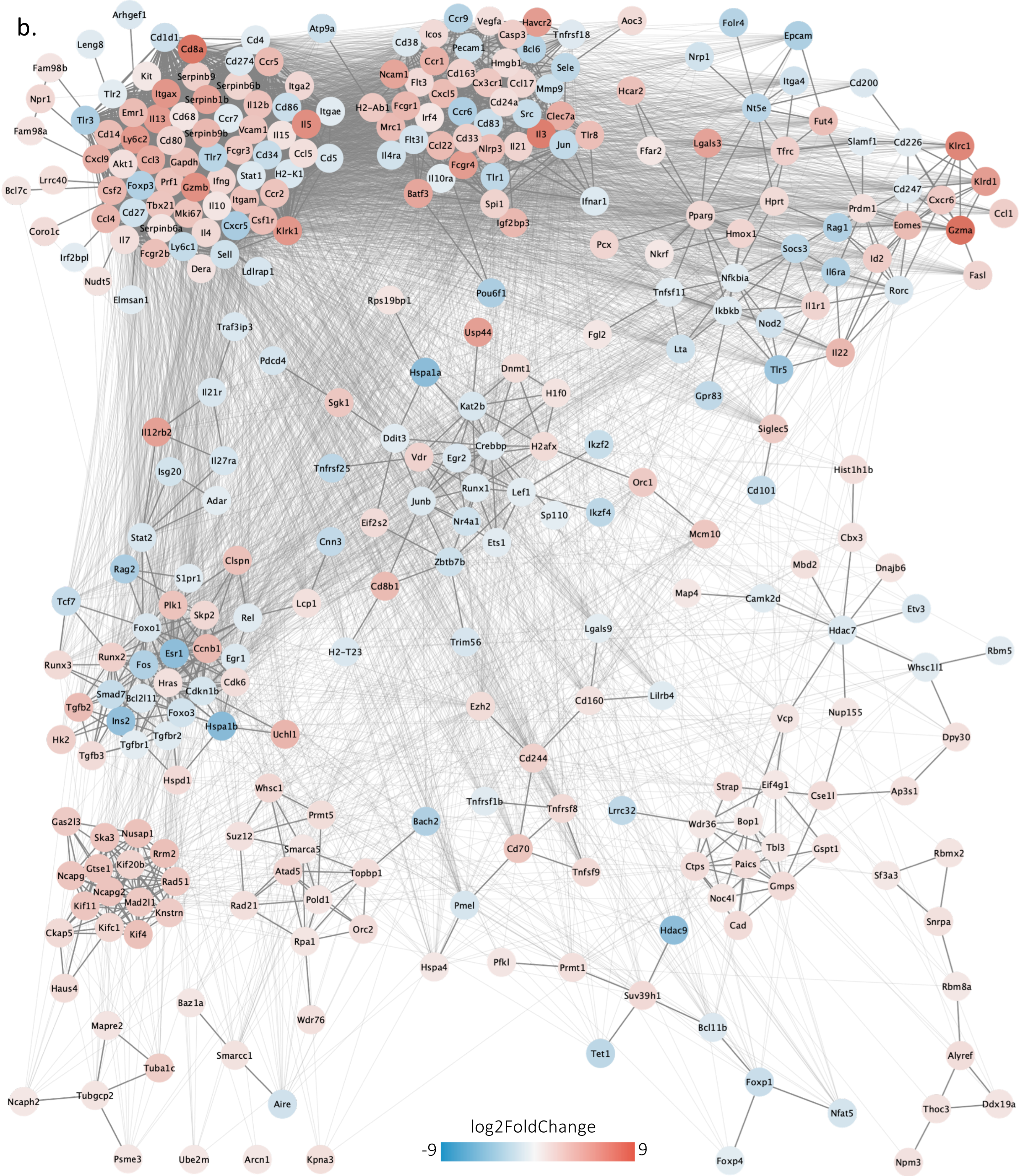

Supplementary Figure 16. RNAseq analysis of CD25+FoxP3+ Tregs from Sin3a-/- or FoxP3cre control mice. Z-scores calculated from fpmk values of DEGs shown by heat map with gene read length, product type, and chromosomal location in columns on the right (a). DEGs associated with FoxP3 within the STRING network are displayed as circular nodes colored on a scale according to their log2 fold change, light and dark lines representing protein association and interaction respectively and organized using Cytoscape MSC clustering (b).

Fig. 17

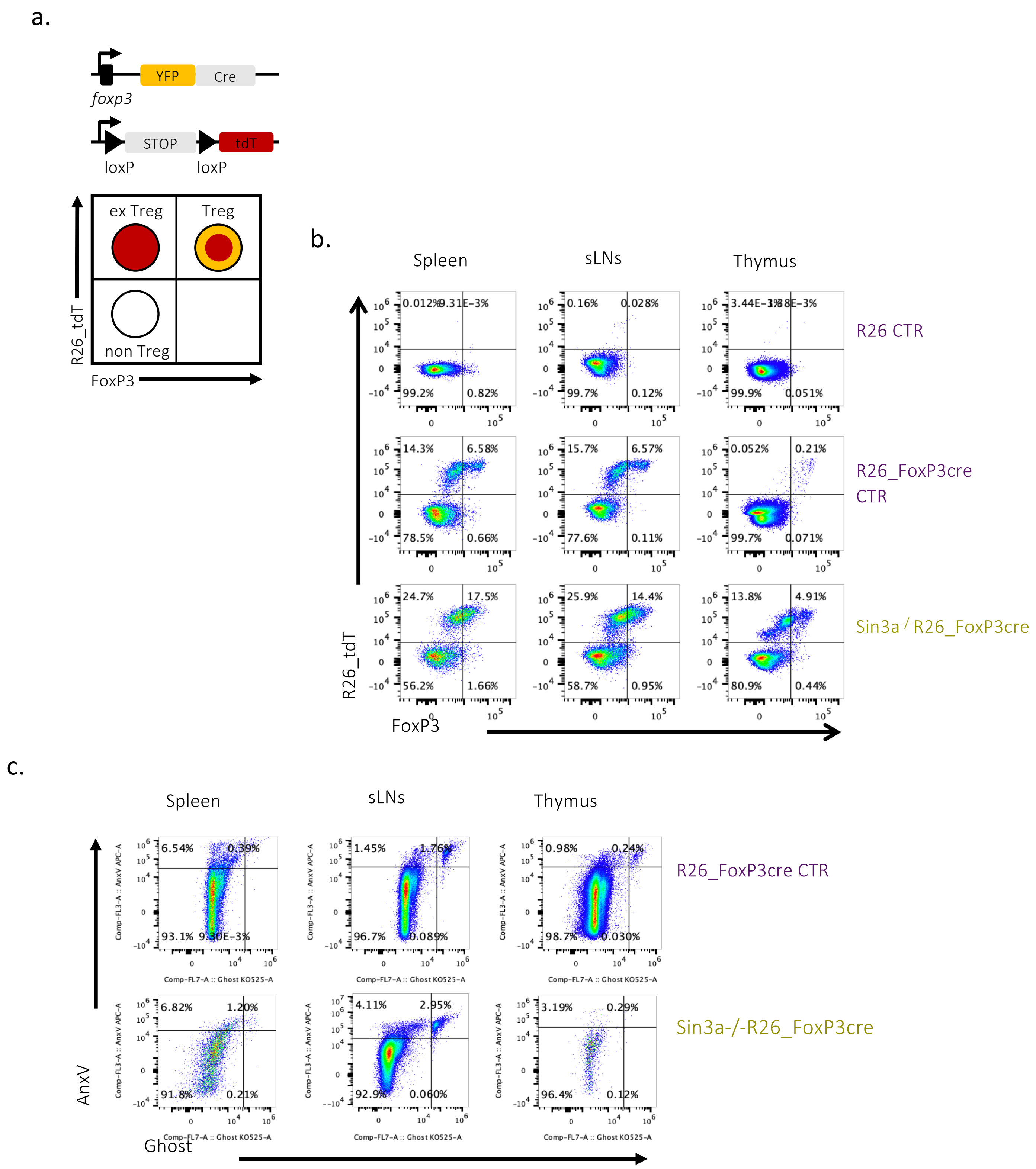

Supplementary Figure 17. Fate mapping Sin3a-/- in FoxP3 Tregs. Representative flow cytometry plots for R26tdT and FoxP3 within live_CD45+CD4+ cells in Sin3a$^{-/-}$, CTR R26, or R26_FoxP3cre mice (**a**) with a diagram of the genetics and FACS analysis of Treg fate mapping using the R26^{STOP}tdTomatoFoxp3^{YFPcre} model (**b**). R26+ cells were gated for AnxV+Ghost- (**c**) in cells from R26^{STOP}tdTomatoFoxp3^{YFPcre} lymphoid organs. Splenocytes were treated with or without uM DCI for 3 or 24 hours in TCM and 37C, in addition each sample was treated with PMA/Ion/Bref for 3 hours prior to being stained for flow cytometry analysis (e).

Fig. 18

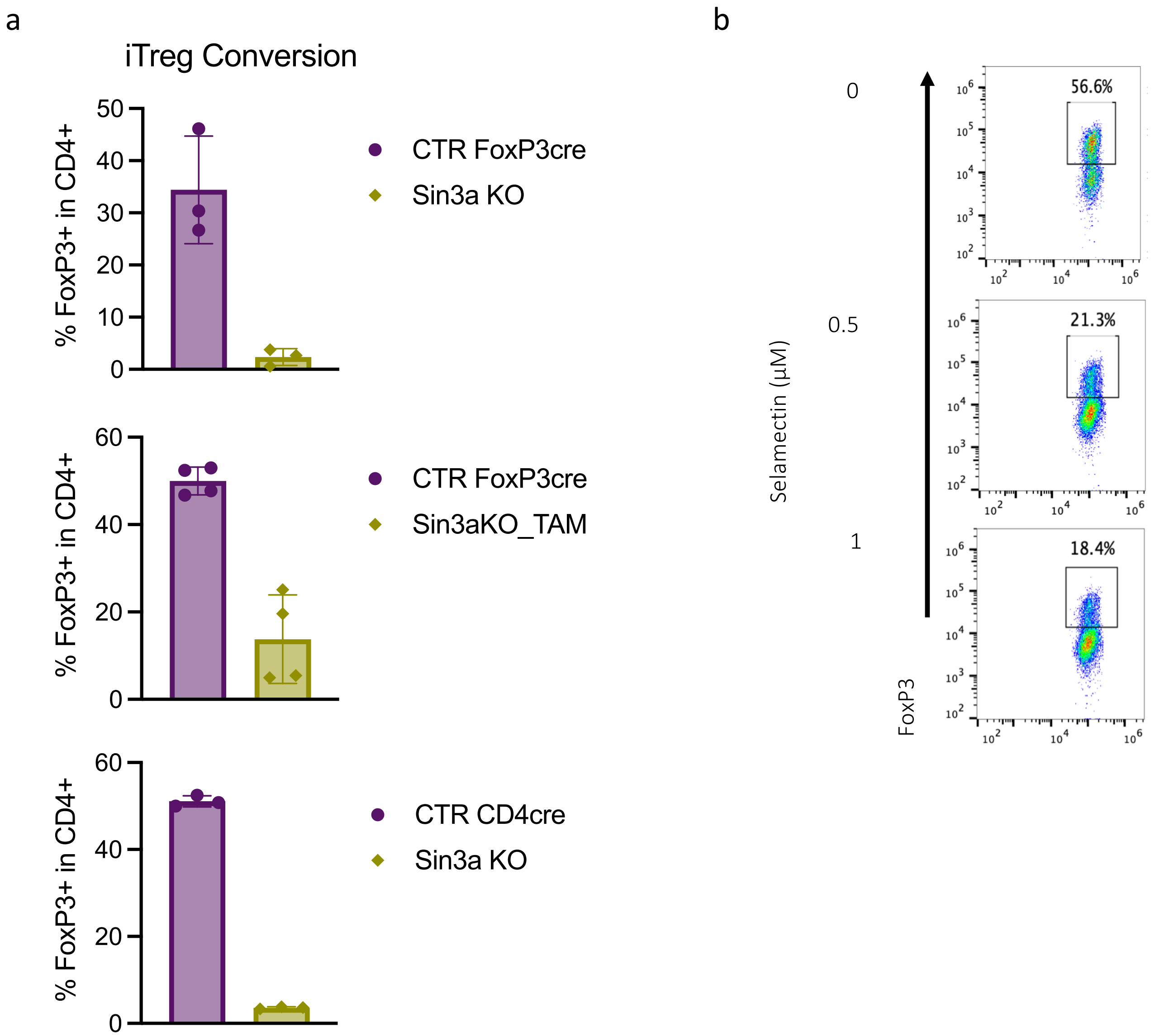

Supplementary Figure 18. iTreg conversion. CD4+CD25- cells stimulated with IL2, TGF-β, and CD3/28 beads for 3 days were then evaluated for FoxP3 production via flow cytometry. The percent of FoxP3 within CD4+ cells from Sin3a-/- within the FoxP3cre (a, top), TAM-induced FoxP3cre (a, center), or CD4cre (a, bottom). These experiments were performed with 3 biological replicates and technical triplicates per group. FoxP3 induction was also evaluated in stimulated control CD4+CD25- cells with or without selemectin treatment (b).

Fig. 19

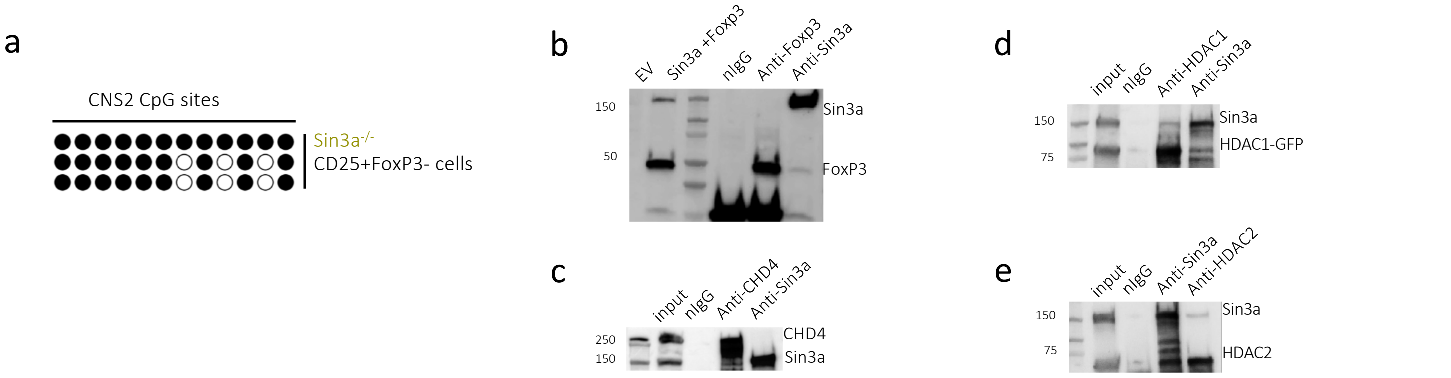

Supplementary Figure 19. CpG site methylation of the CNS2 promoter region of the Foxp3 gene in CD25+FoxP3- cells from Sin3a$^{-/-}$Foxp3^{YFPcre} mice (a) where empty circles represent non-methylated CpG and closed circles represent methylated CpG and each row depicts an individual sample. Sin3a and Foxp3 co-IP in transfected 293T cells (b-e).

Supplementary Table 1. Antibodies.

Target	Dye Conjugate	Clone	Provider
Ghost fixable viability dye	BV510	NA	TONBO biosciences
CD45	FITC	30-F11	BioLegend
CD45	AF700	30-F11	BioLegend
CD45	BV605	104	BioLegend
CD4	PE-CF594	RM4-5	BD Horizon
CD62L	PerCP-Cy5.5	MEL-14	BioLegend
CD25	APC	PC61	BioLegend
CD44	PE	IM7	BD Pharmingen
CD69	PE-CF594	H1.2F3	BD Biosciences
CD8a	BV605	53-6.7	BD Biosciences
CD8a	FITC	53-6.7	BD Pharmingen
CD4	FITC	H129.19	BD Pharmingen
NK1.1	FITC	M1/70	BioLegend
CD11b	FITC	M1/70	BioLegend
CD19	BV605	6D5	BioLegend
CD45R (B220)	BV785	RA3-6B2	BioLegend
IgM	PerCP-Cy5.5	II/41	Invitrogen
CD62L	PE	MEL-14	BioLegend
CD95 (FAS)	PE-CF594	Jo2	BD Horizon
GL7	APC	GL7	BioLegend
CD138	APC-R700	281-2	BD Horizon
CD93	PE/Cy7	AA4.1	BioLegend
CD23	APC Cy7	B3B4	BioLegend
CD21/35	ef450	7E9	BioLegend
CD19	FITC	6D5	BioLegend
CD19	PE	6D5	BioLegend
CD185 (CXCR5)	APC	L138D7	BioLegend
CD279 (PD-1)	PE/Cy7	29F.1A12	BioLegend
FoxP3	ef450	FJK-16s	Invitrogen
CD25	PE	NA	Miltenyi Biotec
GrzB	PE-CF594	GB11	BioLegend
IL-2	PE/Cy7	JES6-5H4	BioLegend
IFN-γ	APC	XMG12	BioLegend
TNF-α	PE	MP6-XT22	Invitrogen
IL-10	APC	JES516E3	BioLegend
IgD	BV650	11-26c.2a	BioLegend
CD1d	PE/Cy7	1B1	BioLegend
CD5	AF700	53-7.3	BioLegend
Ki67	PerCP-Cy5.5	B56	BD Biosciences
CD11b	PE/Cy7	M1/70	BioLegend
CCR2	APC	475301	R&D Biosystems
CCR4	PE	2G12	BioLegend
CCR5	BV650	C34-3448	BD Biosciences
CCR6	BV785	29-2L17	BioLegend
CCR7	PerCP/Cy5.5	4B12	BioLegend
Annexin V	AF647	NA	Invitrogen
β-actin	NA	D6A8	Cell Signaling
FoxP3	NA	ab450	Abcam
HDAC1	NA	ab7028	Abcam
HDAC2	NA	ab12169	Abcam
Sin3a	NA	JA94-31	Invitrogen

Supplementary Table 2. Primer sets used for RT-qPCR, PCR, or sequencing.

RT-qPCR		
Target	Dye	Product #
18S	FAM	REF 4318839
Sin3b	FAM	Mm01247119_m1
FoxP3	FAM	Mm00475162_m1
IFN-γ	FAM	Mm01168134_m1
IL-2	FAM	Mm00434256_m1
IL-10	FAM	Mm01288386_m1
GrzB	FAM	Mm00442837_m1
GrzA	FAM	Mm01304452_m1
CD25	FAM	Mm01340213_m1
CTLA4	FAM	Mm00486849_m1
HDAC1	FAM	Mm02745760_g1
HDAC2	FAM	Mm01193628_m1
IKAROS	FAM	Mm01187877_m1
Bad	FAM	Mm00432042_m1
Bim	FAM	Mm00437796_m1
Bcl2	FAM	Mm00477631_m1
Bcl-xl	FAM	Mm00437796_m1
Spi6/Serpinb9	FAM	Mm00777163_m1
TET1	FAM	Mm01169087_m1
TET2	FAM	Mm00524395_m1
TET3	FAM	Mm00805756_m1
Sin3a	FAM	REF 4331348 (Assay ID: APZTNRZ)

PCR/sequencing	
Primer	Sequence (5’-3’)
SIN3A CO primer F	AGCCAGCCCTGAGACTAGTGATAAAC
SIN3A CO primer REV	GGGGGAATGCTGTGTTTTAGGTATG
FoxP3_TSDR outer F	GGGTTTTGGGATATTAATATATATAGTAAG
FoxP3_TSDR outer R	CCACTATATTAACTAACCCATATAACTAA
FoxP3_TSDR inner F	TTGAGTTTTTGTTATTATAGTATTTGAAGAT
FoxP3_TSDR inner R	ACTAAAAACCTAAAAAACTAAACTAACCAA
M13 Forward (-21)	TGTAAAACGACGGCCAGT
M13 Reverse (-26)	CAGGAAACAGCTATGACC
